# The heterogeneous immune landscape between lung adenocarcinoma and squamous carcinoma revealed by single-cell RNA sequencing

**DOI:** 10.1038/s41392-022-01130-8

**Published:** 2022-08-26

**Authors:** Chengdi Wang, Qiuxiao Yu, Tingting Song, Zhoufeng Wang, Lujia Song, Ying Yang, Jun Shao, Jingwei Li, Yinyun Ni, Ningning Chao, Li Zhang, Weimin Li

**Affiliations:** 1 Department of Respiratory and Critical Care Medicine, Med-X Center for Manufacturing, Center of Precision Medicine, Precision Medicine Key Laboratory of Sichuan Province, Frontiers Science Center for Disease-related Molecular Network, West China Hospital, West China Medical School, Sichuan University, 610041 Chengdu, China; 2 National Cancer Center, National Clinical Research Center for Cancer, Cancer Hospital & Shenzhen Hospital, Chinese Academy of Medical Sciences and Peking Union Medical College, 518116 Shenzhen, China

**Keywords:** Lung cancer, Lung cancer

## Abstract

A thorough interrogation of the immune landscape is crucial for immunotherapy strategy selection and prediction of clinical responses in non-small-cell lung cancer (NSCLC) patients. Single-cell RNA sequencing (scRNA-seq) techniques have prompted the opportunity to dissect the distinct immune signatures between lung adenocarcinoma (LUAD) and lung squamous cell carcinoma (LUSC), the two major subtypes of NSCLC. Here, we performed scRNA-seq on 72,475 immune cells from 40 samples of tumor and matched adjacent normal tissues spanning 19 NSCLC patients, and drew a systematic immune cell transcriptome atlas. Joint analyses of the distinct cellular compositions, differentially expressed genes (DEGs), cell–cell interactions, pseudotime trajectory, transcriptomic factors and prognostic factors based on The Cancer Genome Atlas (TCGA), revealed the central roles of cytotoxic and effector T and NK cells and the distinct functional macrophages (Mφ) subtypes in the immune microenvironment heterogeneity between LUAD and LUSC. The dominant subtype of Mφ was *FABP4-*Mφ in LUAD and *SPP1-*Mφ in LUSC. Importantly, we identified a novel lymphocyte-related Mφ cluster, which we named *SELENOP-*Mφ, and further established its antitumor role in both types, especially in LUAD. Our comprehensive depiction of the immune heterogeneity and definition of Mφ clusters could help design personalized treatment for lung cancer patients in clinical practice.

## Introduction

Immune therapies have presented sustained clinical significance in non-small-cell lung cancer (NSCLC).^1^ However, due to intrinsic genomic and immunogenic heterogeneity, whether a specific form of immunotherapy is effective for different patients may be difficult to predict.^2^ As the major cause of cancer-related mortality, lung cancer accounts for 1.796 million deaths (18%) annually on a global scale.^3^ NSCLC constitutes over 85% of lung cancer cases;^4^ its two main subtypes, accounting for approximately 80% of NSCLC cases, are lung adenocarcinoma (LUAD) and lung squamous carcinoma (LUSC).^5,6^ To uncover the role of the distinct genetic profiles of lung cancer subtypes among patients, new research opportunities have arisen that might contribute to different therapeutic decisions.^7,8^

There are diverse predictors of response to therapy in the tumor microenvironment (TME), including genomic features, transcriptomic signatures and epigenetic modifications, which also contribute to the heterogeneity of clinical response across cancer subtypes and orchestrate either beneficial or adverse outcomes for tumor progression.^9,10^ As a crucial part of the TME, the immune microenvironment contains a comprehensive combination of immune cells including macrophages (Mφ), lymphocytes, monocytes and dendritic cells (DC), and immune checkpoint molecules including programmed cell death-1 (PD-1), programmed cell death ligand 1 (PD-L1), and cytotoxic T-lymphocyte antigen 4 (CTLA-4),^11^ many of which are regarded as putative biomarkers in clinical practice.

Considering the complexity of the tumor microenvironment, single-cell RNA sequencing (scRNA-seq), which reveals comprehensive transcriptome profiling at single-cell resolution with an unbiased catalog of cellular diversity, is a promising tool to extensively investigate the immune heterogeneity in the tumor microenvironment.^1,12^ Compared with bulk sequencing which renders averaging of the signals,^13^ scRNA-seq is uniquely poised to interrogate specific cellular subpopulations and states. However, existing studies have mainly focused on stromal cells and cancer cells in the TME, as exemplified by Lambrechts and co-workers elucidating the heterogeneous nature of stromal cells in the lung cancer TME.^12^ However, Guo and colleagues have characterized the T-cell landscape by single-cell sequencing.^1^ Yet, the dynamics and molecular features of the immune landscape in lung cancer at single-cell resolution remain largely uncharted, let alone the details of the distinct immune atlases between LUAD and LUSC.

To bridge this gap, in this study, we first sought to provide an immune cellular atlas for lung cancer with a comparison between LUAD and LUSC. We conducted scRNA-seq on 40 samples of matched tumor and adjacent normal tissues from 19 NSCLC patients (LUAD: *n* = 10; LUSC: *n* = 9), comprehensively characterized the transcriptomic features of immune cells, decoded dynamic changes in cell percentage, and identified the heterogeneity of cell subtypes and intercellular interactions between LUAD and LUSC. The current study could help explain the difference in the two major NSCLC subtypes through the insights into cellular composition, states and dynamics in the immune microenvironment, and paved the way for the development of future therapeutic targets in the TME for the lung cancer clinical workflow.

## Results

### High-resolution scRNA-seq revealed the immune landscape of LUAD and LUSC

**Fig. 1:**
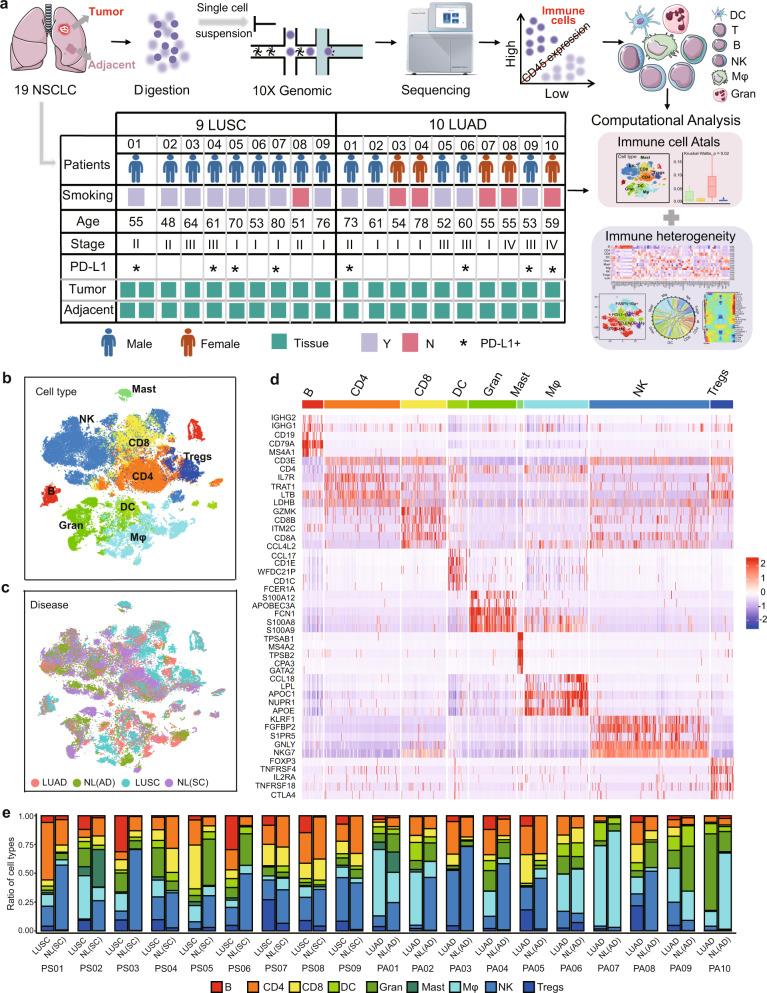
High-resolution immune cell-type landscape of LUAD and LUSC samples in both tumor and corresponding normal tissues. **a** Schematic diagram highlighting the workflow of the study design and analysis. **b** t-SNE plot showing the identified cell types of all immune cells. **c** t-SNE plot of 72,475 single immune cells, colored by disease subtypes. **d** The heatmap showing the expression of cell type-specific marker genes in different immune cells. Mφ (macrophages), DC (dendritic cells), B (B cells), CD8 (CD8+ T cells), Gran (granulocytes), Tregs (regulatory T cells), NK (natural killer cells), CD4 (CD4+ T cells), Mast (mast cells). **e** Fraction of cell types originating from each patient. NL: adjacent normal lung tissue; NL(AD): adjacent normal lung tissue of LUAD; NL(SC): adjacent normal lung tissue of LUSC

To characterize the immune landscape in NSCLC, a total of 40 samples of tumor and adjacent normal lung specimens (NL) from 19 NSCLC patients (10 LUAD and 9 LUSC) were resected and immediately processed to generate a single-cell suspension enriched for viable cells, and the isolated live cells were used directly. Subsequently, scRNA-seq was performed to investigate the immune heterogeneity between the two subtypes of NSCLC (Fig. 1a and Supplementary Fig. 1a). The clinical information and sequencing metrics of these samples are appended in Supplementary Table 1 and Supplementary Table 2, respectively. More technical details, including the quality control criteria and filtering steps, are presented in the Methods and Materials. Furthermore, the dissociation-related genes *FOS*, *FUN,* and *HIF1A*, were all expressed on the formalin-fixed paraffin-embedded (FFPE) tumor samples. Thus they were retained in the subsequent analysis (Supplementary Fig. 1b). CD45, as a pan-leukocyte marker, has been used to define the leukocytes by multiparametric flow cytometry.^14^ Thus, CD45+ cells among all obtained cells were identified as immune cells in our study and selected for subsequent analyses (Fig. 1a). In total, 72,475 immune cells, encompassing 17,277 unique genes, passed quality control, with 38,169 cells from tumor tissues and 34,306 from adjacent normal tissues, and none of these cells were patient- or disease-specific (Fig. 1c and Supplementary Fig. 1c).

All immune cells were sorted into 9 cell types according to the expression level of canonical marker genes as reported previously^1,12^ by using the dimensional reduction method of t-distributed stochastic neighbor embedding (t-SNE),^15^ including B cells, CD4+ T cells (CD4), CD8+ T cells (CD8), regulatory T cells (Tregs), macrophages (Mφ), dendritic cells (DC), granulocytes (Gran), mast cells and natural killer cells (NK) by well-recognized marker genes (Fig. 1b and Supplementary Fig. 1e), with an average of 3558 unique transcripts per cell type (Supplementary Fig. 1d). All cell types were further validated and refined by SingleR analysis.^16^ The common markers of each cell type are visualized as a heatmap (Fig. 1d) and the top 20 marker genes of each cell type are listed in Supplementary Table 3.

Next, we elucidated the fraction of immune cell types in different disease types and each patient (Fig. 1e and Supplementary Fig. 1f). Based on comparisons of NL and tumor tissues of all immune cells in each patient, B cells, Tregs, and mast cells were predominant in tumor tissues, while NK cells and Gran were prevalent in NL tissues, and each cell type included cells from more than one patient (Fig. 1e). The fraction of immune cell types in LUAD, NL(AD), LUSC, and NL(SC) tissues showed similar distributions (Fig. 1e), but we also found that some cell types varied significantly among these disease types, such as B cells, Tregs, and mast cells (Supplementary Fig. 1f). To avoid the influence of library size on the results, the frequency we calculated was the proportion of each cell type in different tumor types. In addition, mast cells, B cells, CD8 cells, and Tregs were also found mainly derived from LUSC, while only Mφ and DC were dominated by cells from LUAD (Fig. 1e and Supplementary Fig. 1f). Taken together, our scRNA-seq analyses dissected the heterogeneity of the immune landscape between LUAD and LUSC and showed that LUSC might obtain higher immune heterogeneity than LUAD.

### The significance of macrophages and lymphocytes contributed to the immune heterogeneity of lung cancer subtypes

Investigation of the key factors that induce the immune heterogeneity between LUAD and LUSC would provide a glimpse of the different responses of lung cancer subtypes to immunotherapy.^17^ First, compared with adjacent tissues, although the co-existence of some immune function-related genes, were co-expressed in the two main cancer subtypes, the *HLA-D* family, *NKG7*, *GNLY*, and immune-related gene *FCGR3A* were enriched in LUAD, whereas LUSC distinctly expressed prognosis-related genes, such as *IGHG3*, *IGHG4*, *IGHG1* and *IGKC* (Supplementary Fig. 2a). In addition, some genes presented cell type-specific expression, thus, we further compared the overlapping DEGs between both subtypes, and found that NK cells, Gran, and CD8 cells shared more common dysregulated genes between LUAD and LUSC, while DC, Tregs, CD4 cells, and Mφ had fewer overlapping genes (Supplementary Fig. 2b). These results indicated the commonalities and differences of immune signatures in the different lung cancer subtypes.

**Fig. 2:**
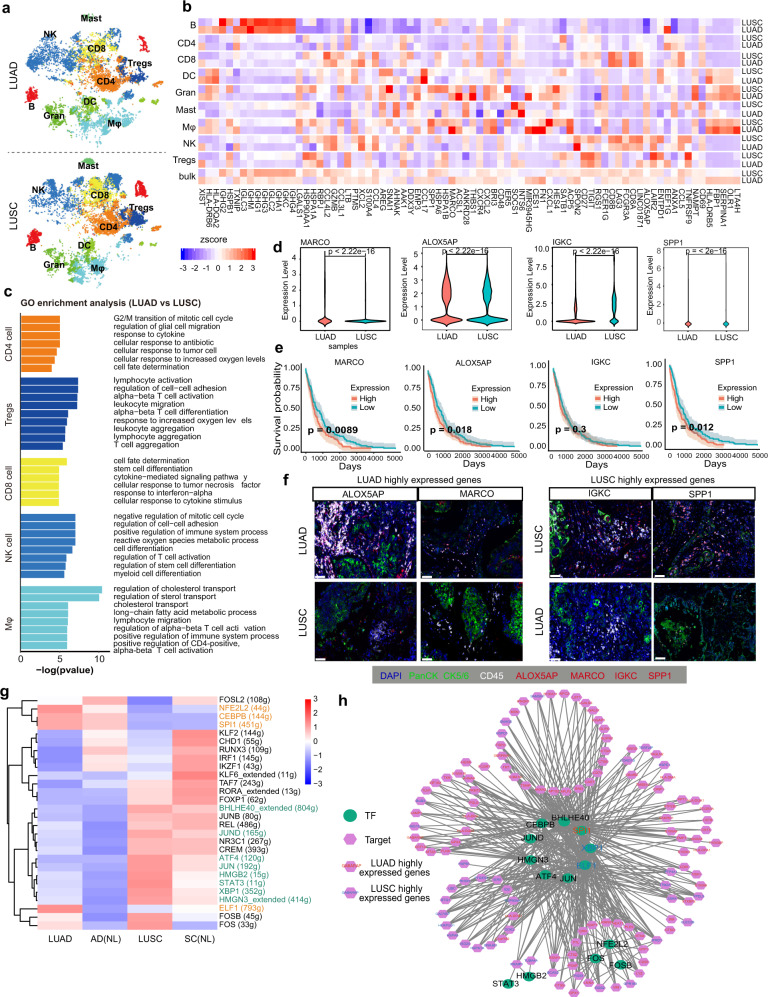
Distinct gene expression patterns of immune cells in the heterogeneity in LUAD and LUSC. **a** t-SNE plot showing the cell type distribution of LUAD and LUSC. **b** The heat map showing the expression of signature genes of each cell type varies between LUAD and LUSC (|logFC| ≥ 0.25 and adjusted *P*-value  ≤ 0.05). The color indicates the effect size. **c** Different colored bar plots showing differences in GO function pathways enriched per cell type by GSEA based on the DEGs between LUAD and LUSC. **d** Violin plots comparing the expression levels of *MARCO*, *ALOXAP5*, *IGKC,* and *SPP1* in all immune cells between LUAD and LUSC. The *P*-values by Wilcoxon tests are shown. **e** Overall survival curves of TCGA LUSC data. **f** Multicolor IHC staining in LUAD and LUSC tissues for verifying the expression of cancer subtype specific genes in immune cells (scale bar = 20 μm). **g** Heat map of gene expression regulation by transcription factors using SCENIC of all the immune cells for different sample origins. The yellow text represents the specific TFs in LUAD, and the green text represents the specific TFs in LUSC. **h** TF-gene regulation networks between different genes of all immune cells in LUAD and LUSC

To further unveil the role of these immune cells in their microenvironment, we then investigated the immune cell type composition and the DEGs in each cell type between LUAD and LUSC. The compositions of B cells and mast cells from different tissues of origin of treatment-naïve patients presented prominent differences between the two cancer subtypes (*P* < 0.05, Kruskal–Wallis tests) (Fig. 2a and Supplementary Fig. 2c), which also shared the fewest overlapping genes (Fig. Supplementary Fig. 2b). All immune cell types displayed cancer subtype-specific features and functions (Fig. 2b, c, and Supplementary Fig. 2d), and some immunoglobulin genes (*IGKC*, *IGHG1*, *IGHG3*, *IGHG4*, and *IGLC2*) were upregulated in each cell type of LUSC tissues compared with LUAD (Fig. 2b and Supplementary Fig. 2e), which were reported to gradually increase in T cells from normal tissues to the tumor and to metastasis in lung cancer.^18,19^ *XIST* was upregulated in LUAD, which promoted lung cancer cell growth. In addition, we noticed that the highly expressed DEGs such as *SPP1*, *CD8A,* and *CD8B* in LUSC were mostly expressed in lymphocytes and myeloid cells, while those highly expressed in LUAD were mostly enriched in myeloid cells, especially Mφ, as exemplified by *FN1*, *LTA4H*, *OLR1*, and *FBP1* (Fig. 2b). As shown in Fig. 2c and Supplementary Fig. 2d, the genes specifically expressed in lymphocytes, except for B cells, were mostly involved in T-cell activation, cytokine response and stem cell differentiation, while myeloid cells, including Mφ, DC, and Gran, expressed genes involved in lipid metabolism-related pathways and immune system regulation, thus culminating in the difference of the immune microenvironment between LUAD and LUSC.

Highly expressed genes in tumors also contributed significantly to the heterogeneity between LUAD and LUSC (Fig. 2d and Supplementary Fig. 2f). Among these genes, *MARCO*, a scavenger receptor on the cell surface of Mφ and *MARCO* + Mφ that enhances regulatory T (Treg) cell proliferation and *IL10* production while diminishes CD8 T-cell activities,^20^ was highly expressed in Mφ; *ALOX5AP* is a 5-lipoxygenase-activating protein that could specifically influence pulmonary function,^21^ and *HLA-DRB5,* belonging to the HLA class II molecule, was highly expressed in both Mφ and Tregs of LUAD; While *IGKC*, previously reported to be expressed in stroma-infiltrating plasma cells and to serve as a prognostic marker in NSCLC,^22^ was validated in our study to be highly expressed in all immune cell types of LUSC. *SPP1*, which mediates Mφ polarization and lung cancer evasion, was shown to be a specific marker in Mφ of LUSC (Fig. 2b).^23^ Furthermore, the high expression of *MARCO*, *ALOX5AP* and *SPP1* were all associated with a poor prognosis in LUSC (Fig. 2e). Immunofluorescence staining also showed the high expression of *ALOX5AP* and *MARCO* in immune cells in LUAD and *SPP1* and *IGKC* in LUSC (Fig. 2f).

To further dissect the immune heterogeneity of gene regulation, the single-cell regulatory network inference and clustering (SCENIC) analysis for DEGs was performed to assess the differences in the expression levels of transcription factors (TFs) between LUAD and LUSC.^24^ Further exploration showed that *NFE2L2*, *CEBPB*, *SPI1*, and *ELF1* were found specifically in LUAD and were mainly enriched in Mφ, while *BHLHE40*, *JUN*, *ATF4*, *STAT3*, and *XBP1* were merely activated in LUSC (Fig. 2g and Supplementary Fig. 2g). PPI network analyses of these TFs and targeted DEGs also revealed similar results. *BHLHE40*, *SPI1*, and *ELF1* shared many target DEGs from LUAD and LUSC (Fig. 2h). These results highlighted that the Mφ and lymphocytes drove the immune heterogeneity of lung cancer. Recent studies on NSCLC also showed that Mφ could induce epithelial-mesenchymal transition (EMT), shape the tumor microenvironment, promote tumor invasiveness, and enhance the Treg cell response and tumor immunity.^25^ Tregs could interact with Th17-like cells to balance the adaptive immune response to tumor antigens.^26^ Thus, Mφ and lymphocytes might play a potentially crucial role in the dynamics of the immune heterogeneity in cancer biology.

Together, we observed distinct immune cell components in the immune microenvironment between both subtypes of lung cancer and identified potential prognostic factors of *MARCO*, *ALOX5AP,* and *SPP1*. We further speculated that the Mφ and lymphocytes were the dominant immune cells that accounted for the differences.

### Cell–cell interaction analysis targeting the key cells with different levels of communication in LUAD and LUSC

**Fig. 3:**
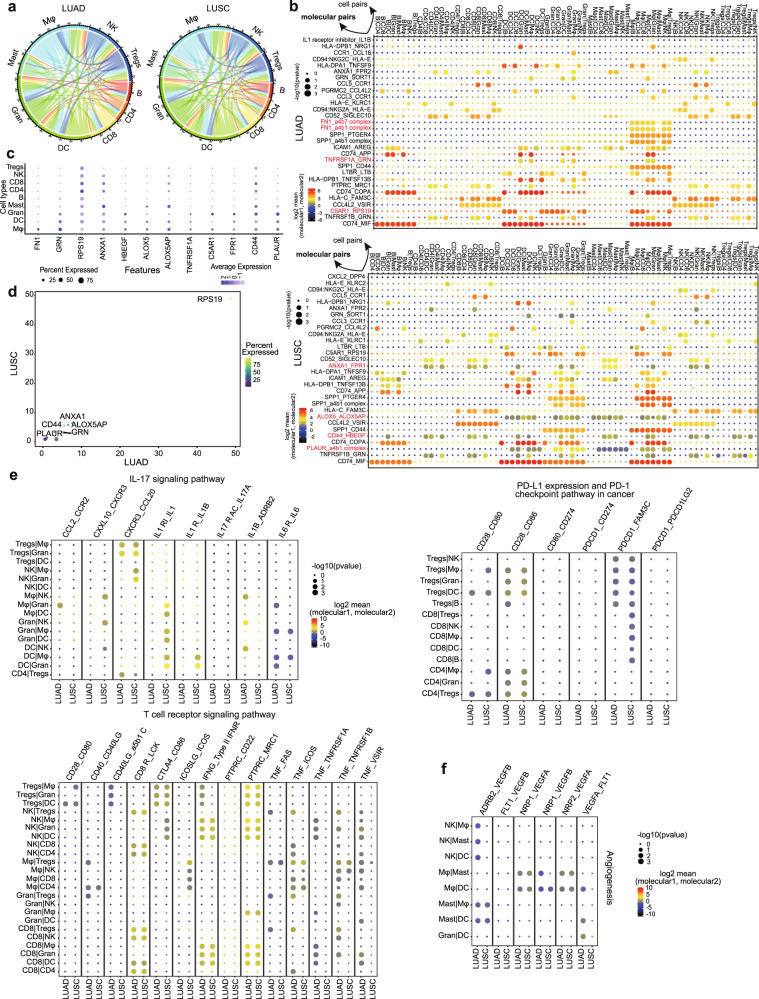
Ligand–receptor interactions between different immune cell types in LUAD and LUSC. **a** Chord plot summarizing interconnections between different immune cell types from LUAD and LUSC. Lines represent potential interconnections between cell types, with line thickness proportional to the number of ligand–receptor pairs expressed in the connected cell types. **b** The molecular interaction pairs of different immune cell type interaction pairs in LUAD and LUSC, and the molecule pair marker in red showing the LUAD and LUSC specific molecular pairs. **c** Dot plot showing the expression of the tumor subtype specific ligands and receptors in each cell type. The color and size indicate the effect size. **d** The expression of specific ligands and receptors in LUAD and LUSC. Bubble size represents the ratio of LUAD/LUSC based on the expression. **e**, **f** Dot plot showing the mean expression level and percentage of selected interaction pairs involved in IL-17, T-cell receptor, PD-L1 expression and the PD-1 checkpoint pathway and angiogenesis. The expression of each gene was considered separately for each sample source

TME is characterized by complex cell–cell interactions among different cell populations that ultimately modulate tumor growth and lead to local invasion/dismal metastasis. To interrogate putative cell–cell interaction heterogeneity in lung cancer subtypes, we deployed a set of immune-related ligand–receptor (L–R) pairs to gain insights into regulatory relationships among all immune cell types identified in our study.^27^ A total of 2612 L–R pairs were predicted to mediate interactions in all immune cells from tumor tissues. And in line with the DEG analysis, Mφ actively interacted with other cell types through a large number of interaction molecular pairs in both LUAD and LUSC (Fig. 3a). Mφ significantly interacted more strongly with cell types of DC, Gran, CD8 cells, B cells and Tregs in LUAD patients and with DC, Gran, NK cells in LUSC patients (Supplementary Fig. 3a). Mφ also harbored the largest numbers of ligands and receptors (Supplementary Fig. 3b). Correlation analyses between Mφ and all other cell types based on the expression data of the top 5 marker genes of each cell type from the TCGA dataset, with *P* < 0.001 in strongly interacting cell types, showcased that Mφ from LUSC had a stronger correlation with DC (*R*^2^: 0.80 vs. 0.75), Tregs (*R*^2^: 0.83 vs. 0.68) and CD4 cells (*R*^2^: 0.71 vs. 0.60) (Supplementary Fig. 3c, d) than Mφ from LUAD.

By comparing the attraction strengths of L–R pairs^27,28^ among all cell pairs, we further uncovered hundreds of specific molecular pairs mediating cell–cell interactions in these cell pairs. The majority of these L–R pairs were shared in NSCLC subtypes, but some distinct L–R pairs were also identified in LUAD and LUSC (Fig. 3b). In LUAD, *TNFRSF1A-GRN* and *C5AR1-RPS19* were enriched among DC, Gran, and Mφ interactions. These genes, except for *RPS19*, were all highly expressed in Gran or Mφ (Fig. 3c) and *GRN* was also found enriched in LUAD tissues (Fig. 3d). The *TNFRSF1A-GRN* pair could directly bind to disturb the *TNFα-TNFR* interaction,^29^ while the *C5AR1-RPS19* could promote tumor growth by facilitating the recruitment of tumor-infiltrating myeloid cells to tumors.^30^ In addition, the specific *FN1-a4b7* and *FN1-a4b1* receptor-ligand complexes indicated the existence of functional interactions between Mφ and all other immune cells (Fig. 3b and Supplementary Fig. 3e). Thus, we suspected that the level of *FN1* might be a significant regulatory factor in the immune microenvironment for LUAD patients. In LUSC, Gran cells expressed higher levels of *PLAUR*, which has been identified as a valuable immune signature with prognostic power in esophageal squamous cell carcinoma.^31^ And the ligand *a4b1* was found mainly in DC and Mφ (Fig. 3b and Supplementary Fig. 3f). *ALOX5-ALOX5AP* was involved in the interaction of Mφ, Gran, and DC. *CD44-HBEGF* and *ANXA1-FRR1*, both related to tumor progression, were also exclusive L–R pairs programming CD4, CD8, DC, Gran, and Mφ interactions (Fig. 3b and Supplementary Fig. 3f). These genes were all expressed in Gran, Mφ and mast cells. *CD44* and *ANXA1* were also expressed in CD4 and CD8 cells (Fig. 3c), and were especially highly expressed in LUAD (Fig. 3d).

To better characterize potential signaling crosstalk between DC, Gran and Mφ, we then subjected the tumor sections from LUAD and LUSC patients to perform multicolor immunohistochemistry (IHC) staining. In LUAD, multicolor IHC staining showed the physical juxtaposition of DC (CD1C^+^) and Mφ (CD68^+^) cells, and the molecular pair *TNFRSF1A-GRN* was also expressed in the two cell types, substantiating the interaction of the DC and Mφ on *TNFRSF1A-GRN* (Supplementary Fig. 3e). The analogous result was also observed for *FN1-a4b1* in DC and Mφ (Supplementary Fig. 3e). In LUSC, we observed the interactions of *ALOX5-ALOX5AP* and *CD44-HBEGF* molecular pairs in DC (CD1C^+^) and Gran (S100A8^+^) cells after multicolor IHC staining (Supplementary Fig. 3f).

Analyses of the biological functions revealed that interactions related to IL-17 signaling as well as PD-L1 expression and the PD-1 checkpoint pathway, including *CXCL10-CXCR3*, *CXCR3-CCL20*, *IL1* receptor-*IL1B*, *IL1* receptor inhibitor-*IL1*, *CD28-CD86,* and *PDCD1-FAM3C*, were more abundant in LUSC, while the interaction pairs in T-cell receptor signaling pathway such as *CD40-CD40LG*, *TNF-ICOS,* and *TNF-TNFRSF1B*, and those related to angiogenesis signaling, exemplified by *VEGFA-FLT1* and *ADRB2-VEGFB*, were more abundant in LUAD than in LUSC (Fig. 3e).

Taken together, immunomodulatory signaling interactions were more abundant in LUAD than LUSC. And those interactions in LUAD were more enriched in therapeutic and prognostic responses, indicating heterogeneity and plasticity of the tumor ecosystem that differed according to the tumor stages. And Mφ may play a role in the inflammation and infiltration during the progression of the tumor, which was consistent with previous studies.^32–34^

### Smoking reshaping the immune landscape of LUAD tissues

Tobacco smoking remains the most established cause of lung carcinogenesis, and the occurrence of LUSC is strongly associated with smoking, while the diagnoses of non-smoking patients have increased drastically in LUAD recent years, especially among women.^7,35^ Thus, we further examined the effects of smoking as a risk factor on the immune microenvironment of lung adenocarcinoma and found that patients with a smoking history had more DC, Mφ and mast cells, while those with a non-smoking history enriched had more Gran, CD4 and CD8 cells (Supplementary Fig. 4a–c). In regard to DEGs, the oncogenic gene *XIST*, the HIF-2α target gene *SCGB3A1*, and tumor necrosis factor (TNF)-targeted gene *HSPA6* were highly expressed in cells from the non-smoking group, while *DDX3Y*, *SCGB3A2,* and *CCL18* were overexpressed in the cells from the patients with a smoking history, particularly in Mφ (Supplementary Fig. 4d). *CCL18* was previously reported to be released from tumor-associated Mφ in breast cancer, promoting angiogenesis and tumor progression,^36^ and we deepened the understanding of this factor in lung cancer.

Functional analyses of DEGs between the smoking and non-smoking groups showed that these genes were mostly from lymphocytes, participating in interleukin-related signaling pathways. In contrast, the genes from myeloid cells were related to T-cell activation, differentiation and cytokine pathways (Supplementary Fig. 4e). GSEA also showed that the genes enriched in the Notch, cytokine and Fc_epsilon_RI signaling pathways were abundant in the smoking group (Supplementary Fig. 4f), while the genes upregulated in non-smoking group could activate *EGFR* tyrosine kinase inhibitor (EGFR-TKI) resistance, ErbB (contributing to resistance to radiation and chemotherapy in cancer), PPAR, Wnt pathways and ECM-receptor interactions (a known pathway contributing to the non-smoking lung cancer) (Supplementary Fig. 4g).

Therefore, we speculated that the immune heterogeneity cast a more significant impact on tumor progression and drug resistance in non-smoking patients than in smoking patients. Mφ and lymphocytes were the cellular bases of such difference.

### Cytotoxic and effector dominant T and NK cells in the immune heterogeneity of distinct lung cancer subtypes

T cells play a central role in immune responses to cancer.^37^ Currently in clinical practice, PD-1/PD-L1 status and CD8 + TILs have been suggested as appropriate measurements to predict different clinical outcomes and inform immunotherapeutic strategies.^38–40^

**Fig. 4:**
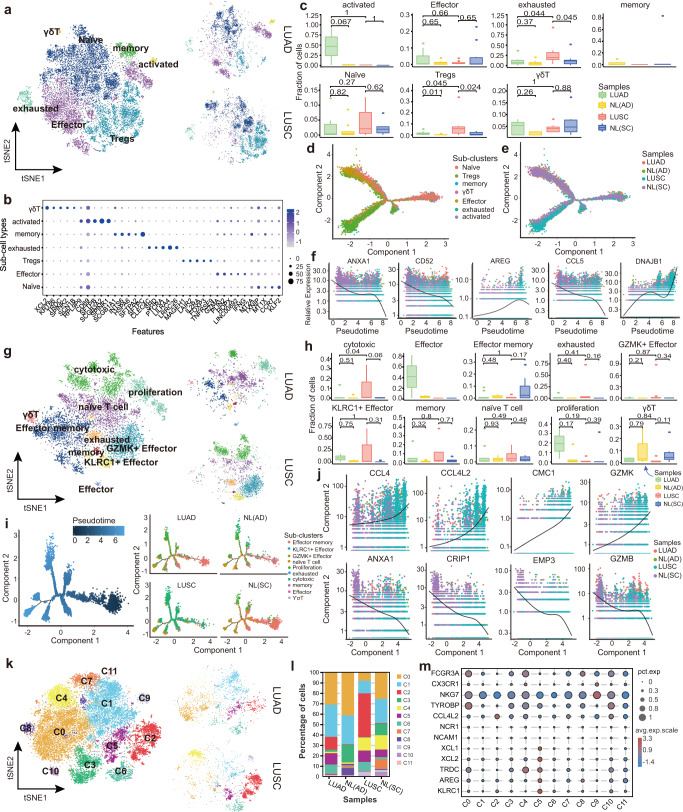
Re-clustering and developmental trajectory of T and NK cells in LUAD and LUSC. **a** Clustering of 15,853 CD4+ T cells from all samples. Each dot corresponds to a single cell, colored according to cell type. **b** Dot plot showing the expression of top 5 marker genes in each CD4 sub-cluster. The color and size indicate the effect size. **c** Percentages of each immune cell type in LUAD, LUSC, NL(SC), and NL(AD). *Y*-axis: Average percentages of samples across the four groups. Groups are shown in different colors. Each bar plot represents one subtype. Error bars represent ± SEM. All differences with *P* < 0.05 are indicated; Kruskal–Wallis rank test was used for analysis. **d**, **e** Developmental trajectory of CD4+ T cells inferred by monocle, colored by cell subtype and sample group. **f** Representative gene expression levels of different marker genes. The size of each dot represents relative expression levels. **g** Clustering of 8735 CD8+ T cells from all samples. Each dot corresponds to a single cell, colored according to cell type. **h** Percentages of each immune cell type in LUAD, LUSC, NL(SC), and NL(AD). *Y*-axis: Average percent of samples across the four groups. Groups are shown in different colors. Each bar plot represents one subtype. Error bars represent ± SEM. All differences with *P* < 0.05 are indicated; Kruskal–Wallis rank test was used for analysis. **i** Developmental trajectory of CD8+ T cells inferred by monocle, colored by pseudotime and cell subtype split by sample group. **j** Representative gene expression levels of different marker genes. The size of each dot represents the relative expression levels. **k** Clustering of 20,584 NK cells from all samples. Each dot corresponds to a single cell, colored according to cell type. **l** Average proportion of each NK subtype between LUAD, LUSC, NL(SC), and NL(AD). **m** Dot plot showing the expression of classic marker genes in each subtype

We identified seven distinct cell subtypes by reclustering 15,853 CD4+ T cells (Fig. 4a, b, Supplementary Fig. 5a–d and Supplementary Table 4) including naïve (*CCR7*^+^ *SELL*^+^), Effector (*IFNG*^+^ *CD44*^+^), Tregs (*IL2RA*^+^ *FOXP3*^+^), exhausted (*PDCD1*^+^ *LAG3*^+^), memory (*S100A4*^+^ *CD44*^+^), activated (*FOS*^+^ *JUN*^+^) and γδT (*TRGC1*^+^ *TRDC*^+^) cells. Specifically, 9,603 (60.58%) CD4+ T cells were obtained from tumor tissues, of which 24.21% were from LUAD and 36.37% were from LUSC tissues (Supplementary Fig. 5a). The distribution of CD4+ T cell subtypes showed that the tumor tissues, compared to adjacent tissues, exhibited an enrichment of Tregs cells and a decline of memory cells. Furthermore, LUAD, compared with LUSC, had more Effector and activated cells as well as fewer Tregs cells (Fig. 4b, c and Supplementary Fig. 5a). Exhausted, Tregs and Effector cells obtained a higher number of DEGs, revealing their important role of them in the tumor immune environment (Supplementary Fig. 5b).

Notably, activated cells, the most enriched subtype in LUAD (Supplementary Fig. 5a), expressed high levels of epithelial cell markers, such as *SFTPB* and *SCGB3A2*, indicating anti-inflammatory features (Fig. 4b). Further development trajectory analyses revealed a similar trajectory between different tumor subtypes and was divided into 3 paths: from the naïve or Effector cells to Tregs, memory and exhausted cells, which was consistent with the previous studies^41,42^ (Fig. 4d, e and Supplementary Fig. 5c, d). Intriguingly, the naïve cells were also expansive at the end state with exhausted cells, which may be due to the higher expression levels of proliferative genes such as *MKI67* (Supplementary Fig. 5c, d). We also found the high expression of *ANXA1*, *CD52* and *CCL5* as well as the depleted expression of *AREG* and *DNAJB1* at the beginning of the developmental trajectory (Fig. 4f).

For CD8+ T cells, ten subtypes were identified by subclustering of 8735 CD8+ T cells^43^ (Fig. 4g, Supplementary Fig. 5e, f and Supplementary Table 5): navïe T cells (*CCR7*^+^ *SELL*^+^), Effector memory (*CD44*^+^ *S100A4*^+^ *ANXA1*^+^), memory (*S100A4*^+^ *CD44*^+^), Effector (*IFNG*^+^ *CD44*^+^), *GZMK*+ Effector (*GZMK*^+^ *CXCR6*^+^), *KLRC1*+ Effector (*KLRC1*^+^ *KLRD1*^+^), cytotoxic (*NKG7*^+^ *GZMA*^+^ *GZMB*^+^), exhausted (*PDCD1*^+^ *HAVCR2*^+^), proliferation (*MKI67*^+^ *STMN1*^+^), and γδT (*TRGC2*^+^ *TRDC*+). The exhausted CD8+ T cells also were characterized by the immune-checkpoint molecules (*PDCD1*, *HAVCR2*, *BTLA*, *TIGIT*, and *CTLA4*), and T cell exhaustion-associated transcription factors *TOX* (Supplementary Table 5). Subsequently, we found that the NL tissues obtained more Effector memory and γδT cells, while tumor tissues had more memory and navïe T cells (Supplementary Fig. 5e). The fractions of CD8+ T cells in two lung cancer subtypes were comparable with that in each other (Fig. 1e), but the Effector cells were enriched in LUAD, and cytotoxic, exhausted, *GZMK*+ Effector as well as *KLRC1*+ Effector cells were abundant in LUSC (Fig. 4h and Supplementary Fig. 5e). The developmental trajectory of CD8+ T cells suggested a multiple branched structures (Fig. 4i, j and Supplementary Fig. 5g): naïve T cells were the root, to diverse end states of a branch, including exhausted, cytotoxic, proliferation, *GZMK*+ Effector and *KLRC1*+ Effector cells, while the Effector memory cells were also arranged at the trajectory start state. Interestingly, the naïve T cells could also be an end state, which was similar to the CD4+ T cells. Furthermore, the trajectory of CD8+ T cells in LUAD was markedly different from that in LUSC, with the proliferation at the end state in LUAD, and cytotoxic, GZMK+ Effector cells in LUSC (Fig. 4i). We also found that during developmental trajectory, *CCL4*, *CCL4L2*, *GZMK*, and *CMC1* were more abundant at the end state while the expression levels of *ANXA1*, *CRIP1*, *EMP3*, and *GZMB* were reduced (Fig. 4j), among which *CCL4* was regarded as a crucial proinflammatory cytokine in lung.^44^

Reclustering 20,584 NK cells revealed 12 subclusters (Fig. 4k–m and Supplementary Fig. 5h): C0-C11, and a distinct sub-cluster composition was observed between LUAD and LUSC. The percentage of each cell subcluster revealed that C0 and C1 cells were enriched in LUAD tissues, while C2 and C4 cells were abundant in LUSC tissues (Fig. 4l). C0 cells and C4 cells both highly expressed *FCGR3A* (CD16), while C0 cells were also characterized by the expression of the cytotoxic marker *TYPOBP* and C4 cells expressed more *TRDC*. Furthermore, the proinflammatory factor *CCL4L2* was more enriched in C2 cells than C1 cells (Fig. 4m). We also noted that the tumor enriched C5 cells were characterized by the expression of resting NK cells markers, such as *AREG*, *XCL1,* and *KLRC1*, while the C9 cells, which expressed the *CX3CR1* and *NKG7*, were abundant in normal tissues.

Taken together, reclustering of the lymphocytes showed distinct cell compositions the two lung cancer subtypes, and the effector, cytotoxic cells contributed to their specific immune response against tumor.

### Distinctive macrophages sub-cluster compositions promoting lung cancer phenotypes

**Fig. 5:**
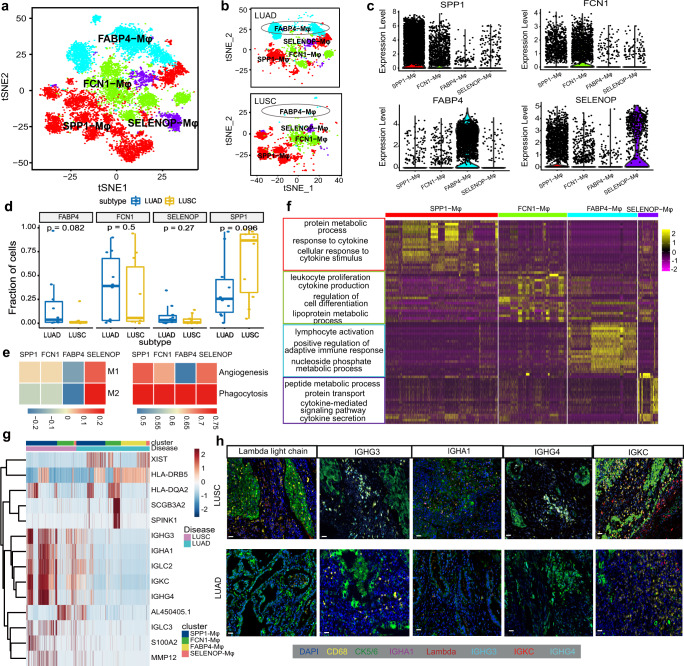
Re-clustering and gene expression patterns of Mφ in LUAD and LUSC. **a** Clustering of 11,016 Mφs from all samples. Each dot corresponds to a single cell, colored according to cell type. **b** t-SNE of Mφ subtype in LUAD (top) and LUSC (bottom). **c** Violin plots of specific marker genes in each Mφ subcluster. **d** Box plot showing the differential expression of *FABP4*, *FCN1*, *SPP1,* and *SELENOP* in LUAD and LUSC. The P values by Wilcoxon tests are shown. **e** Differences in functional activities scored each Mφ subtype by ssGSEA based on the marker genes of M1, M2, angiogenesis and phagocytosis. **f** Heatmap of specific differentially expressed genes of each Mφ subtype and the GO annotation of these genes. **g** Heat map showing the different tumor-related molecules in various Mφ subtypes between LUAD and LUSC. **h** Multicolor IHC staining for verifying the high expression of antibody transcripts in Mφ from LUSC compared with LUAD. The scale bar represents 20 μm

The important role of Mφ in NSCLC development has been established, but the distinct functions they harbored between LUAD and LUSC remain largely uncharted. A previous study indicated that Mφ were further divided into three major groups, namely FABP4^hi^, SPP1^hi^, and FCN1^hi^, and played diverse roles in fibrotic lower lobes in idiopathic pulmonary fibrosis (IPF).^45^ Here, to extensively investigate the heterogeneity of Mφ between the two NSCLC subtypes, 11,016 Mφ were reclustered into four discrete subpopulations based on signature genes, which were defined as *FABP4*, *FCN1*, *SPP1,* and *SELENOP-*Mφ (Fig. 5a–c). However, the expression of the each marker was not exclusively in a unique cluster, as shown in the violin plot (Fig. 5c). Specifically, we observed that 1407 *FCN1* (19.5%), 2873 *SPP1* (39.9%), 2562 *FABP4* (35.6%) and 364 *SELENOP* (5%) Mφ from LUAD tissues, while LUSC samples contained 1381 *FCN1* (36.2%), 1698 *SPP1* (44.6%), 278 *FABP4* (7.3%), and 453 *SELENOP* (11.9%) Mφ. Notably, *FABP4-*Mφ mainly existed in LUAD, and *SPP1-*Mφ were more enriched in LUSC (Fig. 5b, d).

Notably, elevated expression levels of production of immunoglobulin-related genes *IGHG4*, *IGKC*, *IGLC2*, *IGHG3,* and *PLAU* were detected in the *SPP1-*Mφ cluster, suggesting the proinflammatory and anti-tumor functions of this cluster in lung cancer (Supplementary Fig. 6a). While the binding-related molecules, including *S100A8*, *ANXA1*, *VCAN*, *EMP1* and *AREG*, were primarily expressed in the *FCN1-*Mφ cluster (Supplementary Fig. 6a). And the *FABP4-*Mφ enriched the genes closely associated with fatty acids and obesity, including *FABP4*, *CES1*, *HPGD* and *IGFBP2*, which was consistent with a previous study^46^ (Supplementary Fig. 6a). Furthermore, the novel cluster of Mφ we identified, which was named as *SELENOP-*Mφ, highly expressed *SELENOP*. And *SELENOP* has been shown to contribute to the local antioxidant capabilities, thus protecting against inflammatory tumorigenesis.^47^ In our study, the *SELENOP-*Mφ cluster also highly expressed *FOLR2, IL32,* *CD3D* and *LTC4S*, indicating their intimate correlation with lymphocyte-related function (Supplementary Fig. 6a).

Survival analysis of each macrophage subcluster-specific markers based on the transcriptomic data from the TCGA database (513 LUAD and 501 LUSC) showed that increased *AREG, IGHA1, MMP14, PLAU, S100A8* and decreased *HPGD* were inversely correlated with the overall survival of LUSC patients (Supplementary Fig. 6b). Among these genes, *MMP14, PLAU,* and *IGHA1* were specifically upregulated in the *SPP1-*Mφ, suggesting the role of this cluster in the LUSC tumorigenesis (Supplementary Fig. 6a). The increased levels of *SELENOP* and *FOLR2*, markers of the *SELENOP-*Mφ cluster, may help explain the good prognosis of LUAD patients (Supplementary Fig. 6a, c). Hence, we speculated that the *SELENOP-*Mφ cluster might play an antitumor role in LUAD.

To deeply dissect the role of each Mφ subcluster in the development of lung cancer, we interrogated the functions of these cell types by the single sample Gene Set Enrichment Analysis (ssGSEA) score based on the signatures of M1, M2, angiogenesis and phagocytosis.^48^ The LUAD enriched subclusters *FABP4-*Mφ and *FCN1-*Mφ were closely associated with phagocytosis, which is important in immune responses, while the dominant cluster in LUSC, *SPP1-*Mφ exhibited an enrichment of genes related to angiogenesis. Interestingly, all functional types had a high enrichment score in our newly identified cluster *SELENOP-*Mφ, indicating the importance of this cluster in tumor progression (Fig. 5e). The GO analysis also found that the participation of the *SPP1-*Mφ cluster in protein metabolism and cytokine-related pathways. By comparison, leukocyte proliferation, cell differentiation and lipoprotein metabolism were activated in the *FCN1*-Mφ, and lymphocyte activation and nucleoside phosphate metabolism were activated in the *FABP4*-Mφ cluster. In addition, the *SELENOP*-Mφ participated in peptide metabolism, protein transport and cytokine secretion (Fig. 5f). Further analysis of DEGs between LUAD and LUSC showed that the *SCGB3A2* and *SPINK1*, which were overexpressed in Mφ of smoking patients from LUAD (Supplementary Fig. 4d), were augmented in *FCN1*-Mφ (Fig. 5g and Supplementary Fig. 6d). Interestingly, by compared with LUAD, some antibody transcripts, such as *IGHG3, IGHA1, IGLC2, IGKC, IGHG4,* and *IGLC3*, were highly expressed in the *SPP1*-Mφ from LUSC (Fig. 5g, and Supplementary Fig. 6d, e), and immunofluorescent staining also showed that these genes were highly expressed in Mφ in LUSC tissue (Fig. 5h). Therefore, we concluded that Mφ subclusters showcased diverse functional states between LUAD and LUSC.

Together, we have extensively explored Mφ and discovered a novel subcluster, *SELENOP*-Mφ, in addition to three other reported subclusters, which each played a distinct role in LUAD and LUSC.

### Developmental trajectory defined distinct states of Mφ associated with the tumor development

**Fig. 6:**
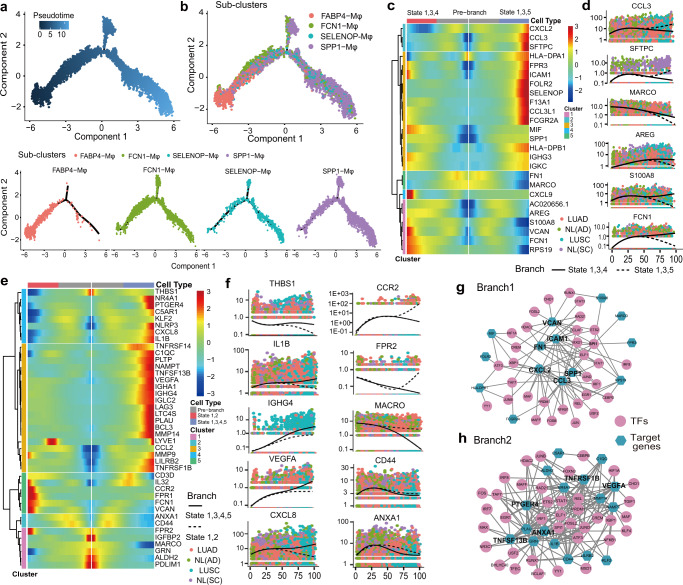
The trajectory analysis of Mφ in LUAD and LUSC revealed distinct features. **a**, **b** Differentiation trajectory of Mφ in all lung samples, with each point colored by pseudotime (**a**) and Mφ subtype (**b**) (bottom) which was divided into two branches. **c**, **d** Pseudo-heatmap showing the various genes in the differentiation process of Mφ in branch 1, which was clustered into five clusters, and a scatter distribution plot showing the expression variation of some specific genes in each state during the pseudotime. The fit curves representing the gene expression of two states, and color coded for cancer type. **e**, **f** Pseudo-heatmap showing the various genes involved in the differentiation process of Mφ in branch 2, which was clustered into five clusters, and the scatter distribution plot showed the expression variation of some specific genes in each state during the pseudotime. The fit curves represent the gene expression of two states, and color coded for cancer types. **g**, **h** Ligand–receptor connections network among TFs and their target genes in branch 1 (**g**) and branch 2 (**h**)

To discern how changes in macrophage subtype-specific gene expression contribute to the tumor immune heterogeneity between LUAD and LUSC. We applied Monocle2 to reconstruct the pseudotemporal trajectory inference of all acquired macrophages (Fig. 6a, b and Supplementary Fig. 7a). The Mφ trajectory yielded five developmental hierarchies (State 1–5) where the *FABP4*-Mφ cluster was located at the starting point of cell evolution on this map (Fig. 6a, b, Supplementary Fig. 7a, b), and suggested a binary branched structure (Fig. 6b): *FABP4*-Mφ as the root, *SPP1*-Mφ at the end state of branch 1, and *SPP1*-Mφ and *FCN1*-Mφ clusters at the end state of branch 2. Notably, the novel subcluster *SELENOP*-Mφ existed throughout the developmental trajectory and accumulated mainly at the end of branch 2 (Fig. 6b and Supplementary Fig. 7a). The developmental trajectory of Mφ in LUAD and LUSC showed obvious differences at the start state, suggesting the importance of *FABP4*-Mφ in the progression of tumorigenesis (Fig. 6b and Supplementary Fig. 7b). We also identified 399 differentially expressed genes that exhibited dynamic expression during the pseudotime of branch 1, which was a subset of those in branch 2 (Supplementary Table 6). And in the State 2, which was the terminal state of branch 2, enriched more cells from NL(AD) than NL(SC) tissues (Supplementary Fig. 7a, b), suggesting that branch 2 may contribute more to the slightly divergent developmental trajectories of Mφ in the adjacent tissues of the two NSCLC subtypes.

Previous studies have indicated that the Mφ promote cancer initiation and malignant progression, and are more correlated with LUSC than LUAD.^12,49^ In this study, we found that some chemokine-related molecules, including *CXCL2*, *CCL3,* and *CCL3L1*, were concentrated in states 1, 3, and 5, which enriched *SPP1*-Mφ and were basically a half-half mixture of LUAD and LUSC at branch 1 (Fig. 6c, d and Supplementary Fig. 7b). In branch 2, some immune checkpoint factors, such as *TNFSF13B, TNFRSF1B,* and *TNFRSF14* were upregulated in state 1, 3, 4 and 5 (Fig. 6e). Additionally, the genes specifically enriched in LUSC included the immune function-related molecules of *MMP9* and *MMP14*, immunoglobulin response checkpoints of *IGHG4*, *IGLC2* and *IGHA1*, tumor angiogenesis molecules of *CXCL8* and *VEGFA*, and the myeloid suppression cell molecules of *THBS1* and *IL1B* (Fig. 6e, f), while in LUAD, some chemokine-related molecules, including *CCR2, CCL2,* and *FPR2*, were increased as well as the T-cell activation factor *CD44* and the T cell activation inhibitor *ANXA1* were highly expressed during the developmental trajectory (Fig. 6c, e, f). Taken together, these results suggested that the differentiation trajectory of Mφ conducted multiple immune functions in the progression of both NSCLC subtypes, but was more evident in LUSC.

Single-cell regulatory network inference and clustering (SCENIC) analysis was performed to assess the differences in the expression levels of transcription factors (TFs) in the process of Mφ differentiation.^24^ The number of TF target genes is listed in Supplementary Table 7. PPI network analysis in branch 1 and branch 2 revealed that the TF target genes enriched in branch 1 were *CXCL2*, *CCL3*, *SPP1,* and *ICAM1*, while those enriched in branch 2 included *TNFRSF1B*, *TNFSF13B*, and *VEGFA* (Fig. 6g, h). Upon further analysis, although LUAD and LUSC shared TFs, they also exhibited exclusive TFs (Supplementary Fig. 7c). For instance, *SPI1* was found in both LUAD and LUSC, while *IRF1* in LUAD and *IRF7* in LUSC were specifically functioned, both of which might serve as a single biomarker for predicting prognosis and metastasis in NSCLC^50^ (Supplementary Fig. 7c).

Collectively, we substantiated that Mφ had a more crucial role in the progression of LUSC, and speculated that the immune microenvironment in LUSC might be more complex than that in LUAD.

### Unique roles of Mφ subclusters in the immune heterogeneity of lung cancer subtypes revealed by extensive analyses of cell–cell interactions

**Fig. 7:**
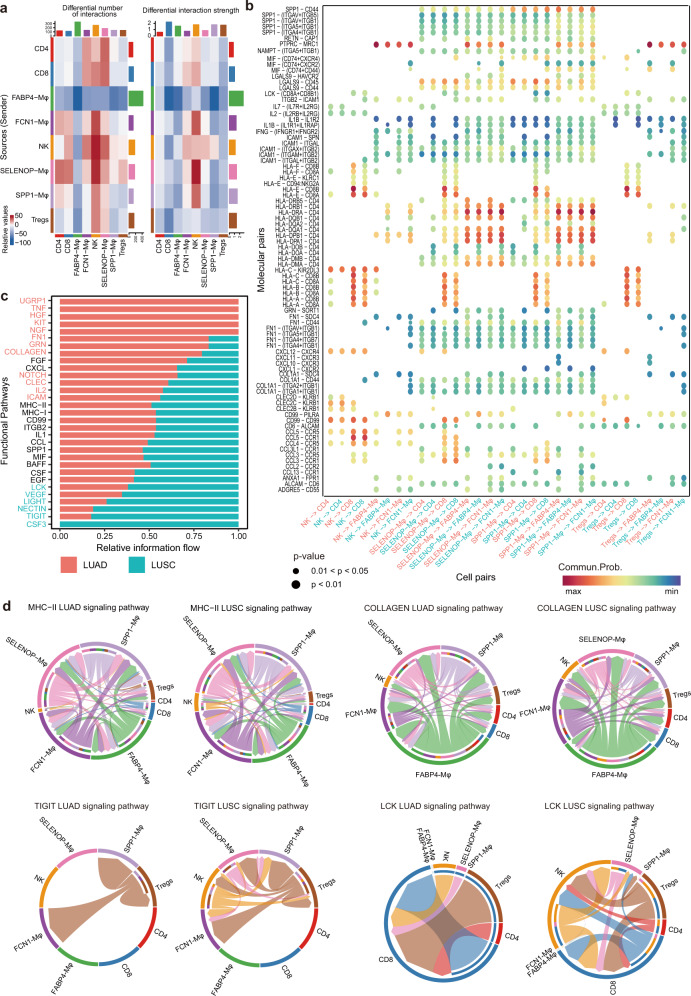
Differential communication patterns between Mφ subclusters and lymphocytes in LUAD and LUSC. **a** Heatmap showing the differential number and strength of interactions between Mφ subtypes and lymphocytes in LUAD and LUSC. The color represents the communication probability. **b** Selected significant ligand–receptor pairs that contribute to the signaling sending between cell types from LUAD and LUSC. The dot color and size represent the calculated communication probability and *p* values. The *p* values are computed from one-sided permutation test. **c** Selected significant signaling pathways were ranked based on their differences in overall information flow within the inferred networks between LUAD and LUSC. The top signaling pathways colored red are more enriched in LUAD, the middle ones colored black are equally enriched in LUAD and LUSC, and the bottom ones colored green are more enriched in LUSC. **d** Chord plot showing inferred intercellular communication network of MHC-II, Collagen, TIGIT and LCK signaling in LUAD and LUSC. Inner and outer bars showing autocrine and paracrine signaling to Mφ subtypes and lymphocytes, respectively. Bar sizes are proportional to the number of cells in each cell group and line width represents the communication probability

Having established that Mφ subclusters played different functions in NSCLC subtypes, and lymphocytes also were identified to contribute to the heterogeneity between LUAD and LUSC, we further investigated the interaction of each Mφ subcluster with lymphocytes. Multiple differential cell interaction pairs were predicted in the two lung cancer subtypes by the R package CellChat^51^ (Fig. 7a and Supplementary Fig. 8a). In LUAD, the interactions between *FABP4*-Mφ and *SELENOP*-Mφ, *SPP1*-Mφ, CD4 cells, CD8 cells, Tregs and NK cells were stronger than those in LUSC, while the *SELENOP*-Mφ and *FCN1*-Mφ obtained enhanced interactions with CD4 and CD8 cells in LUSC (Supplementary Fig. 8a).

Regarding the molecular interaction pairs, more and stronger overexpressed ligands and receptors were identified in LUAD than LUSC (Supplementary Fig. 8b). Chemokine-related pairs, such as *CCL3*-*CCR1*, *CCL3*-*CCR5,* and *CCL5*-*CCR1*, were more activated between *SELENOP*-Mφ/*SPP1*-Mφ and CD8 cells in LUSC. By contrast, the *FN1-*related molecular pairs, including *FN1*-*CD44* (a marker of cancer stem cells) and *FN1*-(*ITGAV* + *ITGB1*), were prompted between *SELENOP*-Mφ/*SPP1*-Mφ and *FABP4*-Mφ/*FCN1*-Mφ/CD4/CD8 cells in LUAD (Fig. 7b). Notably, the human leukocyte antigen genes of *HLA-A/B/C/E* were more closely interacted with the cytotoxic T lymphocyte (CTL) signature *CD8B* of these cell pairs in LUAD, but with *CD8A* in LUSC (Fig. 7b).

Then, we predicted the differential signaling pathways and projected them onto a two-dimensional manifold according to their functional similarity (Fig. 7c and Supplementary Fig. 8c). The various pathways were clustered into 4 groups: Cluster1 to Cluster4, and the signaling pathways belonging to Cluster1 were mainly from LUAD, such as UGRP1, TNF, and HGF. We also identified some signaling pathways enriched in LUSC, including CSF3 (activation of inflammation), TIGIT and VEGF (Fig. 7c and Supplementary Fig. 8c). The MHC-II signaling pathway, which is a predictive biomarker for sintilimab plus chemotherapy in the first-line treatment of locally advanced or metastatic NSCLC, was activated in the Mφ subtype communication with NK cells in LUSC, and with Tregs in LUAD (Fig. 7d). And the SPP1 pathway showed greater cell interaction between the *SPP1*-Mφ cluster and NK cells in LUSC than in LUAD (Supplementary Fig. 8d). The COLLAGEN and the metastasis-related signaling pathway FN1 in LUAD, activated the communication between *SPP1*-Mφ and *FCN1*-Mφ/*FABP4*-Mφ/*SELENOP*-Mφ cluster (Fig. 7d and Supplementary Fig. 8d). The distinct pathways in LUSC, such as TIGIT and cell proliferation related LCK, enhanced the interaction between NK cells and *SELENOP*-Mφ, *SPP1*-Mφ clusters (Fig. 7d). Interestingly, some incoming signaling pathways were found to be specific to one subtype of lung cancer, as exemplified by HGF (a new highlight in the treatments of lung cancer), KIT (activating the JAK/STAT and PLC/PKC signaling pathways) in LUAD, and the CSF3 and CDH5 in LUSC (Supplementary Fig. 8e).

As a result, we hypothesized that Mφ might interact with T cells through ligand–receptor pairs, which are crucial in the tumor progression and the formation of immune heterogeneity between LUAD and LUSC. The connections of the *FABP4*-Mφ cluster and CD8, CD4 cells were more prevalent in LUAD, while interactions of *SPP1*-Mφ, *FCN1*-Mφ clusters and CD8, NK cells were enriched in LUSC. *SELENOP*-Mφ, as a novel cluster, played key roles in both LUAD and LUSC, as revealed by our cell–cell interaction analyses.

## Discussion

In the present study, we have depicted a large compendium of a high-resolution single-cell immune landscape in NSCLC, and identified significant differences in the immune microenvironmental signatures between LUAD and LUSC. We revealed that Mφ and lymphocytes contributed significantly to the immune heterogeneity between two major subtypes of NSCLC patients. These findings can serve as a valuable reference for further explorations to obtain detailed biological insights and to develop new immune checkpoints or therapeutic targets.

Further exploration of lymphocytes demonstrated the key roles of cytotoxic and effector T and NK cells in the immune landscape of NSCLC subtypes. Reclustering Mφ identified four distinct functional subclusters, and uncovered their specific cellular interactions with lymphocytes in the two tumor subtypes. Importantly, we identified a novel lymphocyte-related subcluster named *SELENOP*-Mφ which highly expressed *FOLR2*, *IL32,* *CD3D, and LTC4S*. Through survival analyses based on established TCGA dataset, we hypothesized that the *SELENOP*-Mφ cluster might play an antitumor role in LUAD.

In this study, Mφ cells, with a very high number of DEGs, had the second fewest genes shared in LUAD and LUSC, playing a crucial role in a comprehensive portrait of the immune heterogeneity in cancer biology. Generally, Mφ in the two NSCLC subtypes showed similar but slightly divergent developmental trajectories in tumor progression. It was plausible that Mφ conducted more immune functions in the progression of LUSC compared than in LUAD. Furthermore, among the Mφ subtypes, the dominant subclusters were *FABP4*-Mφ in LUAD and *SPP1*-Mφ in LUSC. *FABP4*, previously was reported in breast cancer to be a functional marker in macrophages^52^, and highly expressed *SPP1* in macrophages would contribute to lung fibrosis.^45^ Our current study supplemented the roles of these two biomarkers in macrophages in shaping the heterogeneity between LUAD and LUSC.

Mφ function essentially in shaping the tumor microenvironment (TME), tumor immunity and response to immunotherapy, which makes them a valuable target for cancer treatment.^25,50^ Our study presented a heterogeneous nature of the Mφ in the lung cancer setting, and identified that the Mφ might have divergent effects according to their different subsets, either pro- or anti-tumor, which cast doubt on the M1/M2 Mφ polarization system. Mφ phenotypes can be more complex, the potential use of which as potential biomarkers or treatment targets should be further explored. In survival analyses, enriched *ARG, IGHA1, MMP14, PLAU, S100A8* and diminished *HPGD* in LUSC, as well as decreased *SELENOP* and *FOLR2* in LUAD were all significantly correlated with a worse survival in NSCLC patients.

Moreover, we found that the function of each cluster in Mφ was regulated by unique TFs in LUAD and LUSC patients, and the majority of TFs were significantly activated in *SPP1*-Mφ and *FABP4*-Mφ clusters (Fig. 6e). According to previous researches, TFs, along with mRNAs, miRNAs, and proteins, constitute the interaction and regulatory mechanisms of NSCLC progression. TFs of *FOXM1* and *MYBL2* were overexpressed in tumors and were associated with the dysregulation of the cell cycle and enhancement of cell proliferation in NSCLC.^53,54^ To conclude, we supported the regulatory roles of TFs and specified the influence on two Mφ clusters.

In our study, we clarified the role of a specific Mφ cluster: the *SPP1*-Mφ cluster. We identified that this cluster highly expressed immunoglobulin-related genes, such as *IGHG4, IGKC, IGLC2, IGHG3,* and *PLAU*, as well as tumorigenesis-related genes like *MMP14, PLAU,* and *IGHA1*. In the cell–cell interaction analyses, *MIF-TNFRSF14*, *SPP1*-CD44, and *SPP1*-*PTGER4* were all identified in the *SPP1*-Mφ cluster. The role of the *SPP1*-Mφ cluster was established in both LUAD and LUSC patients, especially in the LUSC cohort. According to previous studies, the *SPP1* + TAMs are closely related to cancer-associated endothelial cells and fibroblasts, thus modulating the tumor microenvironment. This role has been investigated in lung cancer, colorectal cancer, and breast cancer.^25,55^ The current study further illuminated the distinct impact of the *SPP1*-Mφ cluster in LUSC, with larger fractions and more active interactions with other immune cells.

Our study still has certain limitations worth noting. First, the current techniques cannot distinguish the tumor tissues from the tumor microenvironment, and resections conducted with the human eye can mistake the boundaries between the two types of components. In this case, the identification and statistical analyses can be inaccurate. Second, we did not perform in-depth in vivo and in vitro experimental validations of our findings. Therefore, the actual clinical value of our results entails further explorations. Third, the relationships between different Mφ subtypes and the potential linkages between the Mφ and B cells were not fully investigated. Finally, as a retrospective study, the intrinsic bias was inevitable.

The initial objective of our study was to examine the immune cells in both tumors and adjacent tissues and to decode the differences between LUAD and LUSC. In our study, high-resolution single-cell RNA sequencing, differentially expressed genes (DEGs), pseudotime, transcription factor, reclustering and SCENIC analyses were the relevant tools and measurements that we adopted to detect the various dimensions of gene expression variances. We determined the roles of Mφ, described the properties of different Mφ subtypes, found genetic points that might serve as treatment targets, and explored the prognostic factors.

In the era of precision medicine, immunotherapy might be a revolutionary treatment approach for malignant diseases like NSCLC. Thus, assaying the immune environment of tumors and depicting various subtypes of immune responses has become imperative.^56^ Our study has provided a comprehensive depiction of the NSCLC immune landscape at single-cell resolution, and identified inter-subtype differences in the immune microenvironmental signatures. Future studies are required to clinically and experimentally validate our findings in evaluating the immune cell populations and their prognostic value in patients.

## Methods and materials

### Sample collection and patient characteristics

With approval from the Ethics Committee of West China Hospital, Sichuan University, we collected tumor and adjacent normal tissues from 19 pathologically diagnosed NSCLC patients (10 LUAD and 9 LUSC) during surgical resections, and rapidly digested the tissues to obtain single-cell suspensions. All patients were diagnosed with primary lung tumors and untreated. Their ages varied from 48 to 80, with a median of 57. The stages of these patients were determined by the 8th TNM Classification. Clinical characteristics including age, sex, smoking status, pathological subtype and stage are listed in Supplementary Table 1.

### Preparation of single-cell suspensions

Freshly obtained resected tissues were rinsed with Hanks’ Balanced Salt Solution (HBSS) after the operation, subsequently shredded on ice to smaller pieces with collagenase I/IV in HBSS, and incubated for 30 min at 37 °C with manual shaking every 10 min. The digested tissues were then passed through a 70-μm nylon mesh filter, and the cell suspension were centrifuged at 500*g* for 5 min at 4 °C. After removing the supernatant, the pelted cells were suspended in red blood lysis buffer, and next resuspended in buffer (0.04%BSA + PBS) after being washed with HBSS. Cell suspensions, after depleting dead cells through flow cytometry, were directly processed for single-cell RNA-seq, following the manufacturer’s instructions. Alternatively, cell suspensions were frozen in 20% Dimethyl Sulfoxide (DMSO) and Fetal Bovine Serum (FBS). The cDNA library was constructed within 24 h.

### Single-cell RNA sequencing

We mixed a single-cell suspension with 0.4% trypan blue dye at a ratio of 9:1. Cells were counted using a Countess^®^II Automated Cell Counter. The proportion of living cells was calculated and should exceed 90% for quality control, and the proper concentration of cells should be no less than 1000 cells/μL. We adopted short-read long sequencing and microfluidic techniques to simultaneously analyze the transcriptome expression profile of 500–10,000 cells in each sample. PCR amplification was performed using cDNA as the template. First, the cDNA enzyme was broken into fragments of approximately 200–300 bp, and mixed with the sequencing joint P5 as well as the sequencing primer R1, which was the conventional process in traditional second-generation sequencing. Finally, PCR amplification was performed. In this way, we constructed a standard sequencing library.

The double-ended sequencing mode on the Illumina sequencing platform was utilized to conduct our high-throughput sequencing of constructed libraries. At the Read1 end, information regarding the 16 bp barcode and the 10 bp UMI was used to quantify the cell number and expression level. At the Read2 end, the cDNA fragment served as the reference in genomic alignment to determine the gene to which the mRNA corresponded to. Libraries were sequenced on the Illumina NovaSeq 6000 platform at West China Hospital, Sichuan University, Chengdu. On average, each sample generated 200 Gb of raw data and a total of 3.9 T of data were available.

### Alignment and quantification

We obtained 40 samples from 19 individuals, and the raw gene expression matrices were generated using CellRanger (version 3.0.1). Information was processed in R (version 3.6.0) using the Seurat R package (version 2.3.4). We further selected high-quality cells to be preserved, with the following criteria: 1) the number of genes identified in a single cell ranged from 200 to 8000; 2) the total number of UMI in a single cell was less than 50,000; 3) the proportion of mitochondrial gene expression in a single cell, the indicator of apoptosis-related cell condition, was less than 25%. For the potential dissociation-related genes, such as FOS and FUN, we first conducted immunohistochemistry on the formalin-fixed paraffin-embedded (FFPE) tumor samples for these genes. If they were not expressed on FFPE, we removed these genes in subsequent analysis.

### Visualization

In Seurat, t-SNE (t-distributed stochastic neighbor embedding), the nonlinear dimension reduction method, was used to map high-dimensional cellular data into a two-dimensional space, bringing together cells with similar expression patterns and further separating cells with different expression patterns further apart. The differences between the cells were thus made more comprehensible. Subsequently, we used SingleR to make annotations for each cell type. SingleR identified cell types based on similarities in expression patterns between the cells to be identified and the reference cells.

### Cell–cell interaction analysis

We inputted the single-cell gene expression matrix and analyzed the ligand–receptor information contained in the CellPhoneDB software. We only considered receptors and ligands expressed by cell clusters that were above a percentage of the user-specified threshold (the default was 10%). The abundance was the indicator used to evaluate the number of expressed ligand–receptor pairs, allowing for a preliminary assessment of the communication between cells. To identify biological associations, we used CellPhoneDB software to conduct pair comparisons between all cell types in the dataset and to target the significantly enriched ligand–receptor pairs in each pair of cells. Furthermore, the cell interaction network map was constructed to illustrate the regulatory relationship between cells. Moreover, we also used the NicheNet software to identify the influential ligands in the interacting receptor signaling cells. The weighted network of ligand-target gene regulation was depicted, which we referred to score the regulatory potential of the ligands in the receptor signaling cells.

To unravel the differential communication of cell types between LUAD and LUSC, we inputted the single-cell gene expression matrix and analyzed based on the ligand–receptor information contained in CellChat software with the default parameters, which modelled the communication probability and identified significant communications. To identify functional pathway changes, we also delineated signaling changes across LUAD and LUSC.

### Transcription factor analysis

Based on the single-cell RNA-seq results, we used the SCENIC software to infer the regulatory network of transcription factors. We regarded each regulatory network as a regulon. Through the analysis of the regulon activity in each cell type, we determined the differences in the regulatory activity of transcription factors among different cell types.

We adopted GENIE3 to target the important genes with significant numbers, used Rcistarget to determine regulons based on the StarGet dataset, and utilized AUCell to quantify the activity of regulons using the AUC value. Different regulons may contain the same target genes, through the common target genes. We connected all transcription factors and used Cytoscape to illustrate the regulatory network between transcription factors and target genes.

The Spearman correlations between TFs and target genes in each module were calculated, and each module was divided into two sub-modules, either activation or inhibition, according to the positive and negative values of correlation.

### Pseudotime analysis

Pseudotime analysis, also called cell trajectory analysis is commonly used to predict the evolutionary trajectory of cell subtypes and apoptosis pathways, or to infer the differentiation trajectory of stem cells during the disease progression. In the current study, we performed pseudotime analysis by using Monocle 2, an analysis tool based on the expression patterns of key genes. According to the pseudotime value, Monocle modelled the gene expression level as a smooth and nonlinear pseudo time function to show the gene expression changes with time. FDR < 1e−5 was regarded as significantly different.

### Survival and correlation analyses

We used TCGA data to evaluate the prognostic value of cell-cluster specific genes and the correlation of inter-cell clusters. We downloaded the transcriptomic expression and clinical data of 1014 NSCLC patients (513 LUAD and 501 LUSC) from the TCGA database by the R package TCGAbiolinks. For survival analysis, all patients with LUAD or LUSC were divided into two groups (high expression and low expression) based on the mean value of the specific gene in these samples, then modelled by the survival package and visualization. For correlation analysis, the mean values of the top 5 marker genes from the LUAD or LUSC dataset in each cell type were represented as the expression value of the cell type in the public dataset. Then, we conducted the correlation analysis between macrophages and other cell types by the ggstatsplot package. A *P*-value < 0.01 was regarded as significantly different.

### The Opal multiplex immunohistochemistry, mIHC

The Opal multiplex IHC staining method based on tyramine signal amplification (TSA) technology allows the detection of more than seven different markers on the same tissue slice using different colored dyes combined with spectral imaging and quantitative analysis software, therefore, the abundant in situ tissue information can be accurately presented. In this study, the Opal Polaris 7-color Manual IHC Kit was used for the multistaining of 4 µm sections of clinical paraffin tissue after dewaxing and rehydration with xylene and gradient alcohol solution. Note that only double-distilled water and freshly prepared wash buffer working solution should be used for tissue washing throughout the experiment; and the samples should not be exposed to tap water. The staining process of Opal technology is similar to the normal immunohistochemical staining, except that only one antibody can be incubated in each round of staining, and an antibody elution step is added to remove the noncovalently bound antibody from the antigen by microwave elution, but retain the TSA fluorescence signal covalently bound to the antigen surface, thus achieving direct antigen labeling without antibody interference. In this experiment, 4 kinds of antibodies needed to be stained on one tissue, so 4 times incubations of primary antibody, secondary antibody, and TSA signal amplification incubation and 5 times of antigen repairs were needed. The 4th incubation was followed by the last antigen repair, then DAPI staining and Fluoromount-G sealing. Specific detailed experimental steps can be referred to the kit instructions and adjusted as appropriate. The equipment used to implement spectral imaging is a multispectral tissue imaging system (PerkinElmer Vectra^®^), and the software used for pathology results analysis was Phenochart 1.0 and PerkinElmer inForm.

The antibodies used in the experiments were: CD1C (ORIGENE, TA505411S, mouse), CD68 (Biolegend, 916104, mouse), S100A8 (proteintech, 15792-1-AP, rabbit), ALOX5 (5 Lipoxygenase/5-LO, abcam, ab169755, rabbit), ALOX5AP (ATLAS ANTIBODIES, HPA026592, rabbit), CD44 (Affinity, DF6392, rabbit), HBEGF (Santa, sc-365182, mouse), TNF-R1 (Santa, sc-8436, mouse), GAN (ORIGENE, TA807351S, mouse), hlntegrin α4β1 (R&D, MAB10603, human), FN1 (Fibronectin, abcam, ab2413, rabbit), CK5/6 (Millpore, MAB1620,mouse), PanCK (abcam, ab7753, mouse), CD45 (abcam, ab40763, rabbit), IGHA1 (ProMab, 31070, mouse), Lambda Light chain (abcam, ab124719, rabbit), IGKC (abcam, ab134929, rabbit), IGHG3 (abcam, ab193172, rabbit), IGHG4 (HUABIO, ET1609-56, rabbit), SPP1 (abcam, ab214050, rabbit), MARCO (abcam, ab231046, rabbit), and ALOX5AP (ATLAS ANTIBODIES, HPA026592, rabbit).

### Immunohistochemistry (IHC)

Tissues were immediately fixed in 10% neutral formalin solution for 48–72 h after surgery. The dehydration instrument completes the graded dehydration and immerses the tissues in wax. The 4 μm sections were baked in a 67 °C oven for more than 4 h after the paraffin embedding, and then deparaffinized in xylene and graded ethanol in distilled water. Immunohistochemistry uses a two-step method. Antigen retrieval was performed with a microwave in a water bath with Tris-EDTA solution for 2 * 8 min. Then, endogenous peroxides were blocked in 3% H2O2 for 15 min at room temperature, Subsequently, the corresponding primary antibody working solution was incubated at 4 °C overnight, and the secondary antibody, goat anti-rabbit IgG (Dako, Shanghai, China) is incubated at 37 °C for 65 min, the sections were incubated with DAB working solution for 3 min. Finally, hematoxylin was used to counterstain the cell nuclei, and the sections were dehydrated, cleared and fixed with neutral gum. The immunohistochemical pictures were collected by an Olympus IX83 microscope. The antibodies used in this study were: c-Jun (Abcam, ab32137, rabbit), c-Fos (Abcam, ab222699, rabbit), HIF-1 (Abcam, ab51608, rabbit).

### Statistical analyses

All statistical analyses were performed using R (version 3.6.0), and two-tailed *p* values were used to evaluate the statistical significance. In our study, *p* values were calculated through Bonferroni correction, and |log2FC| ≥ 0.25, *P* < 0.05 was considered statistically significant.

In the Seurat package, the rank-sum test was used to analyze the differential gene expression in different cell subpopulations. We then used a barrier model-based analysis of single-cell transcriptomics (MAAST) to examine the significance of the differential genes and corrected the significant *p* values with multiple tests.

To test whether there were significant differences between LUSC and LUAD, LUSC and LUSC_normal, LUAD and LUAD_normal in the fraction of each cell type in our study. First, we calculated each cell type fraction in each sample. Then, the Kruskal–Wallis test, which is a one-way ANOVA with the observations replaced by ranks, was used to detect the significance of the differences in cell fractions between the above three groups, with *P* < 0.05 considered statistically significant.

### GSEA analyses

By using GO function and KEGG pathway significant enrichment analysis, we analyzed the biological functions of the differentially expressed genes. Through quantification of the significant enrichment in pathways, the major biochemical metabolic and signal transduction pathways involved in differentially expressed genes were identified. To predict the functional features of Mφ subclusters, we also used the single sample gene set enrichment analysis (ssGSEA) analysis approach to score the functional signatures, which were downloaded from previous studies.

## Supplementary information

Supplementary Materials

## Data Availability

All single-cell sequencing raw data of this study are available in Science Data Bank (ScienceDB, https://www.scidb.cn/en) by visiting 10.57760/sciencedb.02028. The other resources used in this study are available from the corresponding authors upon reasonable request.
