# Supplementary material for: The heterogeneous immune landscape between lung adenocarcinoma and squamous carcinoma revealed by single-cell RNA sequencing
Source: Signal Transduct Target Ther. 2022 Aug 26;7:289. doi: 10.1038/s41392-022-01130-8 (PMC9411197; doi:10.1038/s41392-022-01130-8)
Supplement: Supplementary file 1 — Supplementary Materials [file 41392_2022_1130_MOESM1_ESM.docx]

Supplementary Materials for

**The heterogeneous immune landscape between lung adenocarcinoma and squamous carcinoma revealed by single-cell RNA sequencing**

Chengdi Wang^1^, Qiuxiao Yu^2^, Tingting Song^1^, Zhoufeng Wang^1^, Lujia Song^1^,

Ying Yang^1^, Jun Shao^1^, Jingwei Li^1^, Yinyun Ni^1^, Ningning Chao^1^, Li Zhang^1^, Weimin Li^1^

These authors contributed equally: Chengdi Wang, Qiuxiao Yu, Tingting Song

**Correspondence**:

Chengdi Wang (chengdi_wang@scu.edu.cn); Weimin Li ([weimi003@scu.edu.cn](mailto:weimi003@scu.edu.cn)); Li Zhang ([zhangli7375@scu.edu.cn](mailto:zhangli7375@scu.edu.cn))

**This PDF file includes:**

Figures. S1 to S8

Tables S1 to S7

**
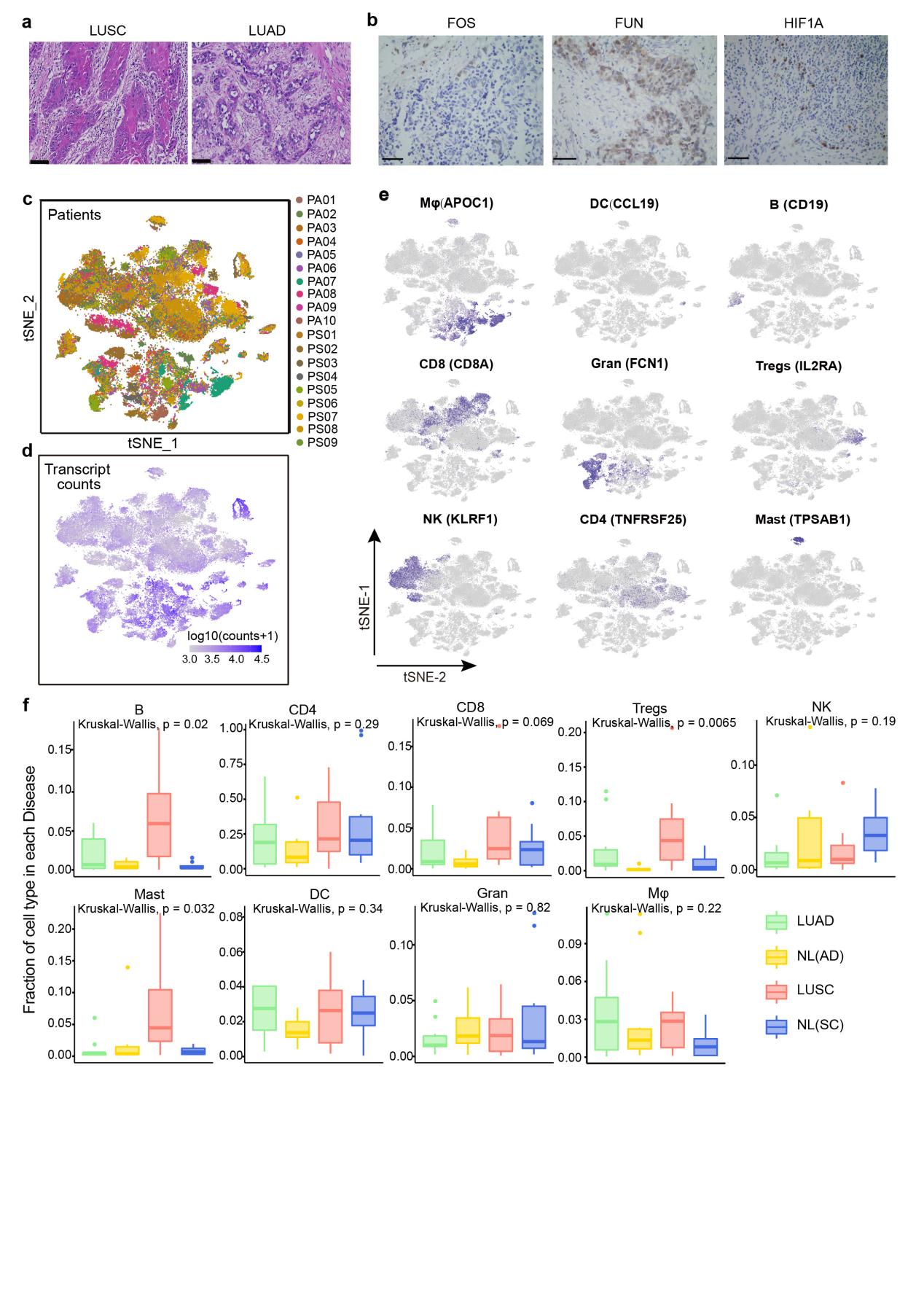
**

**Figure. S1. High-resolution immune cell-type landscape of LUAD and LUSC in both tumor and corresponding normal tissues.**

**a** Hematoxylin and eosin stain (HE) of LUAD and LUSC (scale bar = 100 μm). **b** Immunohistochemistry (IHC) showing the expression of dissociation-related genes (*FOS, FUN, HIF1A*) on the formalin-fixed/paraffin-embedded（FFPE）tumor samples (scale bar = 20 μm). **c** Cell distribution of all samples, colored by patients. **d** Distribution of transcript counts in all cells. **e** t-SNE plot showing distinct DEGs in various immune cell types. **f** Bar-plot showing the temporal alterations of each immune cell type based on the fraction of cells in each disease type. *P*-value was calculated by Kruskal-Wallis test.


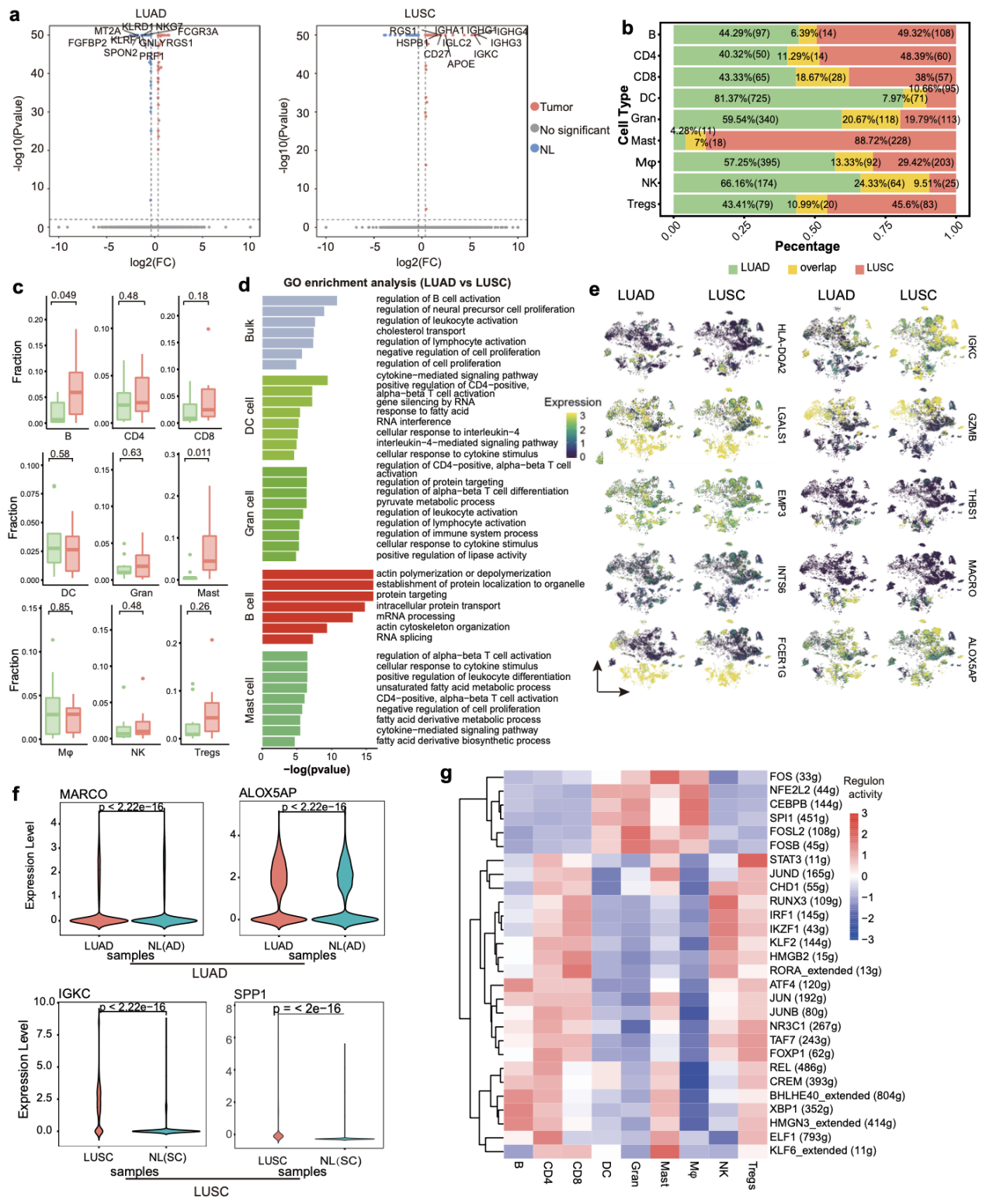


**Figure. S2. Distinct gene expression patterns of immune cells showing the heterogeneity between LUAD and LUSC.**

**a** Volcano plot showing DEGs in all immune cells between LUAD and LUSC (|logFC|≥0.25 and adjusted *P*-value ≤0.05 ). Top 10 genes were labeled on the plot. Points colored in blue represent the genes specific in tumor samples, while those in red represent the genes specific in adjacent tissues. **b** Bar plot showing up-regulated genes specific in LUAD, specific in LUSC and overlapped in both types across immune cells. Up-regulation were defined by comparing LUAD or LUSC tumors with their paired adjacent normal tissues. **c** Distinct fractions of each immune cell type between LUAD and LUSC, with the y axis representing the fraction of cells. Groups are shown in different colors. Each bar plot represents one subtype. Error bars represent ± Standard Error of Mean (SEM). All differences with *P* < 0.05 identified by one-sided Kruskal-Wallis rank test are regarded as significant. **d** Differently colored bar plot showing differences in GO function pathways enriched across cell types by GSEA based on the DEGs between LUAD and LUSC. **e** t-SNE plot of DEGs in LUAD and LUSC. **f** Violin plots comparing the expression of *MARCO, ALOXAP5, IGKC* and *SPP1* in all immune cells between tumor and normal (NL) tissues. *P*-values by Wilcoxon tests are shown. **g** Heatmap of gene expression regulation by transcription factors across cell types by SCENIC.

**
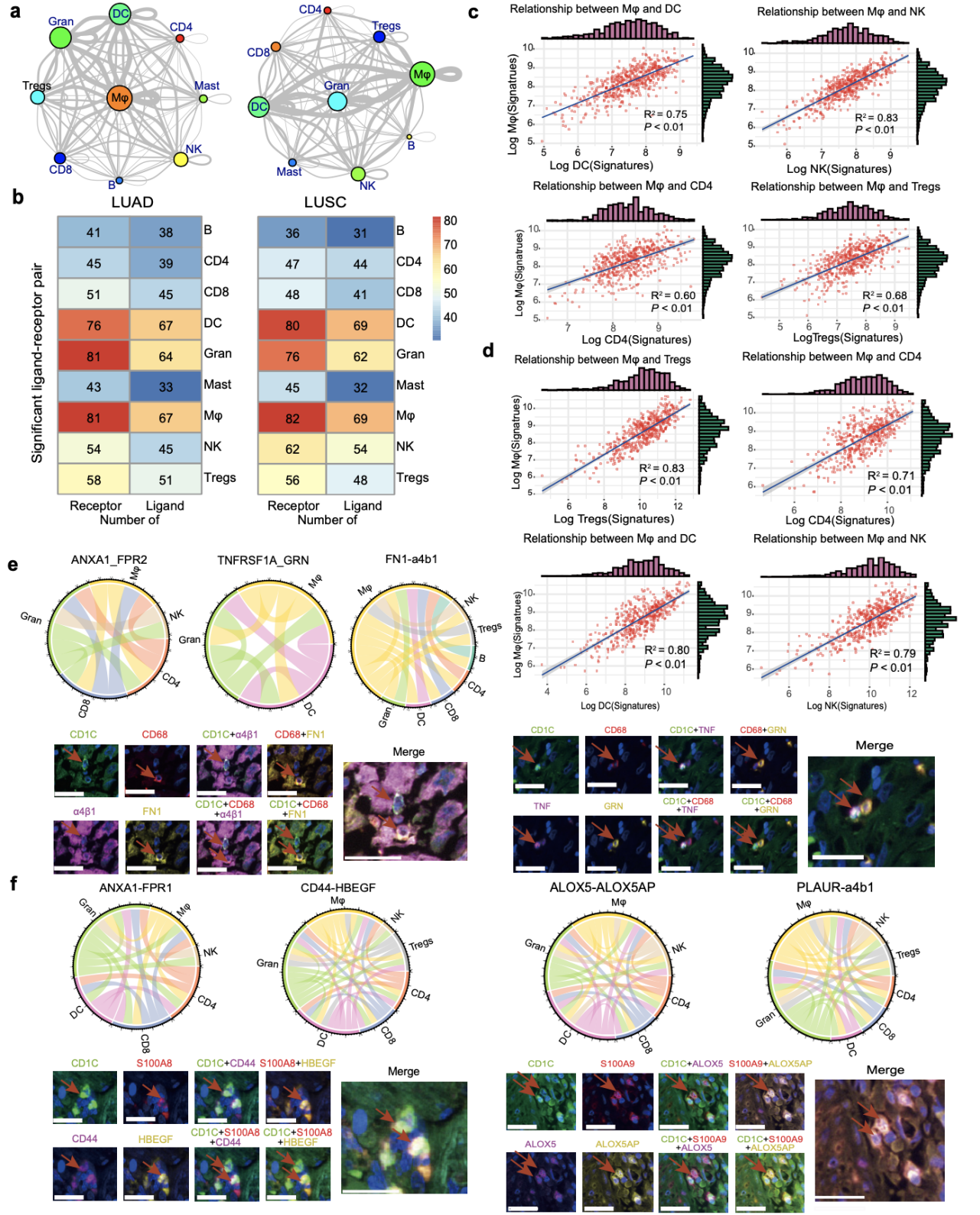
**

**Figure. S3. Ligand-receptor interactions among different immune cell types between LUAD and LUSC.**

**a** Network plots summarizing interconnections among different immune cell types from LUAD and LUSC. Lines represent potential interconnections between cell types, with line thickness proportional to the number of ligand-receptor pairs expressed in the connected cell types. **b** Numbers of ligand-receptor pairs among the various immune cells in LUAD and LUSC. **c,d** Correlations of macrophages and other immune cell types in LUAD (**c**) and LUSC (**d**) based on the transcriptome data from TCGA database. **e,f** Chord plot showing the predicted interactions mediated by *TNFRSF1A-GRN, FN1-a4b7, ANXA1-FRP2* and *ALOX5-ALOX5AP , ANXA1-FRP1, CD44-HBEGF, PLAUR-a4b1* molecular pairs in LUAD (**e**) and LUSC (**f**), and the multicolor IHC staining showed the interactions of *TNFRSF1A-GRN, FN1-a4b1* in DC and macrophages of LUAD and *ALOX5-ALOX5AP* and *CD44-HBEGF* in DC and Gran cells of LUSC (scale bar = 20 μm).

**
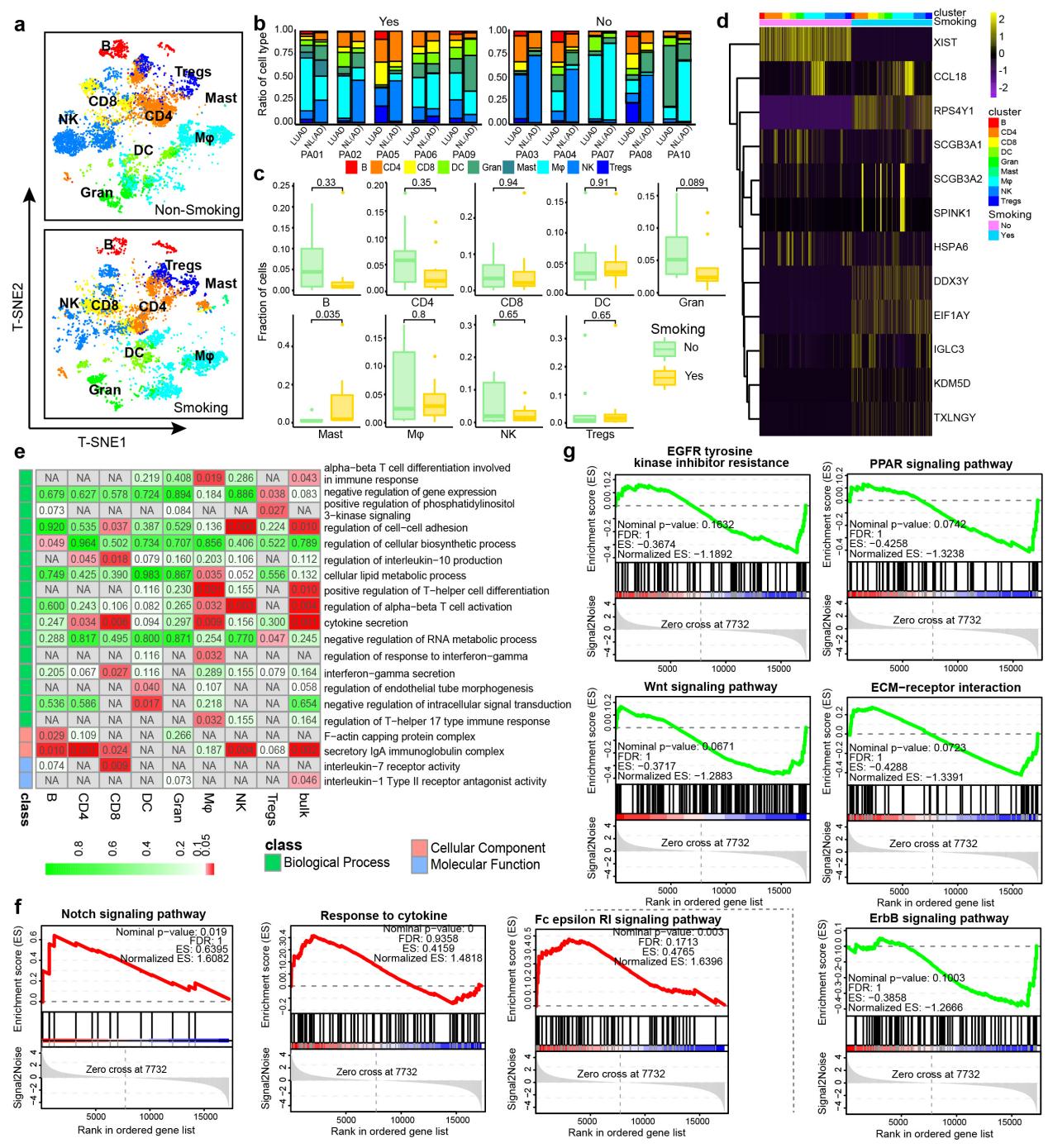
**

**Figure. S4. Distinct immune microenvironment of LUAD between smoking and non-smoking patients.**

**a** t-SNE plot showing the cell-type distribution of smoking samples and non-smoking samples from LUAD tissues. **b** Fractions of cell types originating from each patient. **c** Percentages of each immune cell type in smoking and non-smoking samples. The y axis represents average percentage of samples between the two groups. Groups are shown in different colors. Each bar plot represents one subtype. Error bars represent ± SEM. All differences with *P* < 0.05 identified by one-sided Kruskal-Wallis rank test are regarded as significant. **d** Heat map showing DEGs of all immune cells between smoking and non-smoking samples. **e** GO enrichment pathways of DEGs across each cell types between smoking and non-smoking groups. **f,g** GSEA enrichment scores (ES) of diverse pathway activities in all immune cells between smoking (**f**) and non-smoking (**g**) groups.

**
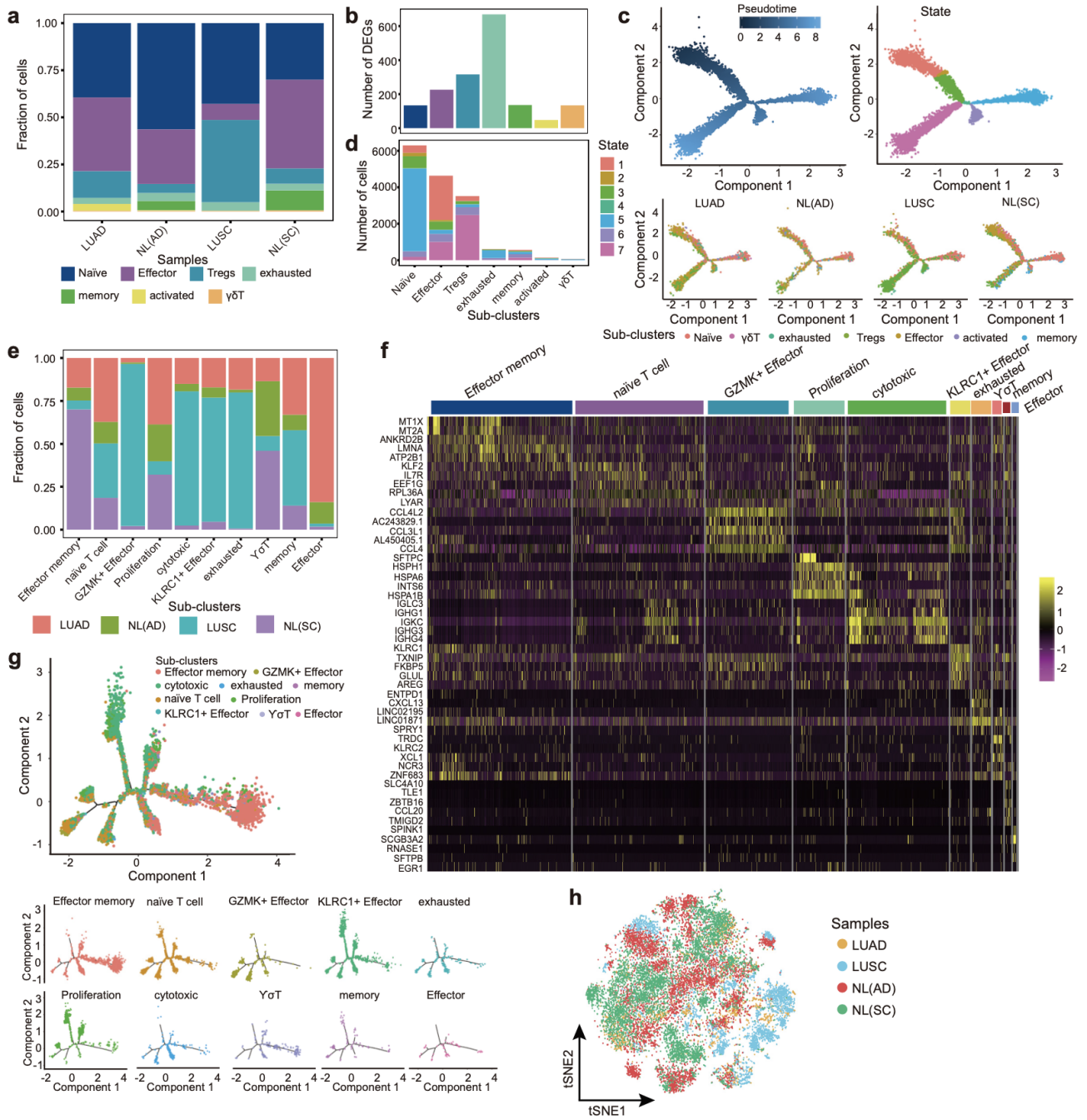
**

**Figure. S5. Re-clustering and developmental trajectory of T and NK cells of different sample origins.**

**a** Average proportion of each CD4 subtype among LUAD, LUSC, NL(SC) and NL(AD). **b** Numbers of DEGs of each subtype between LUAD and LUSC patients. **c** Development trajectory of CD4+ T cells inferred by Monocle 2, colored by pseudotime, state, or sample type. **d** Bar plot showing numbers of cells in different states. **e** Fractions of cell clusters. Color represents sample origin. The x axis represents different cell types, and the y axis shows the composition of sample origins. **f** Heatmap plot showing diverse DEGs among cell types. **g** Development trajectories of CD 8+ T cells inferred by Monocle 2, colored and distinguished by subtype. **h** t-SNE plot showing distribution of each NK subtype cells from all samples. Each dot indicates a single cell, colored according to sample type.

**
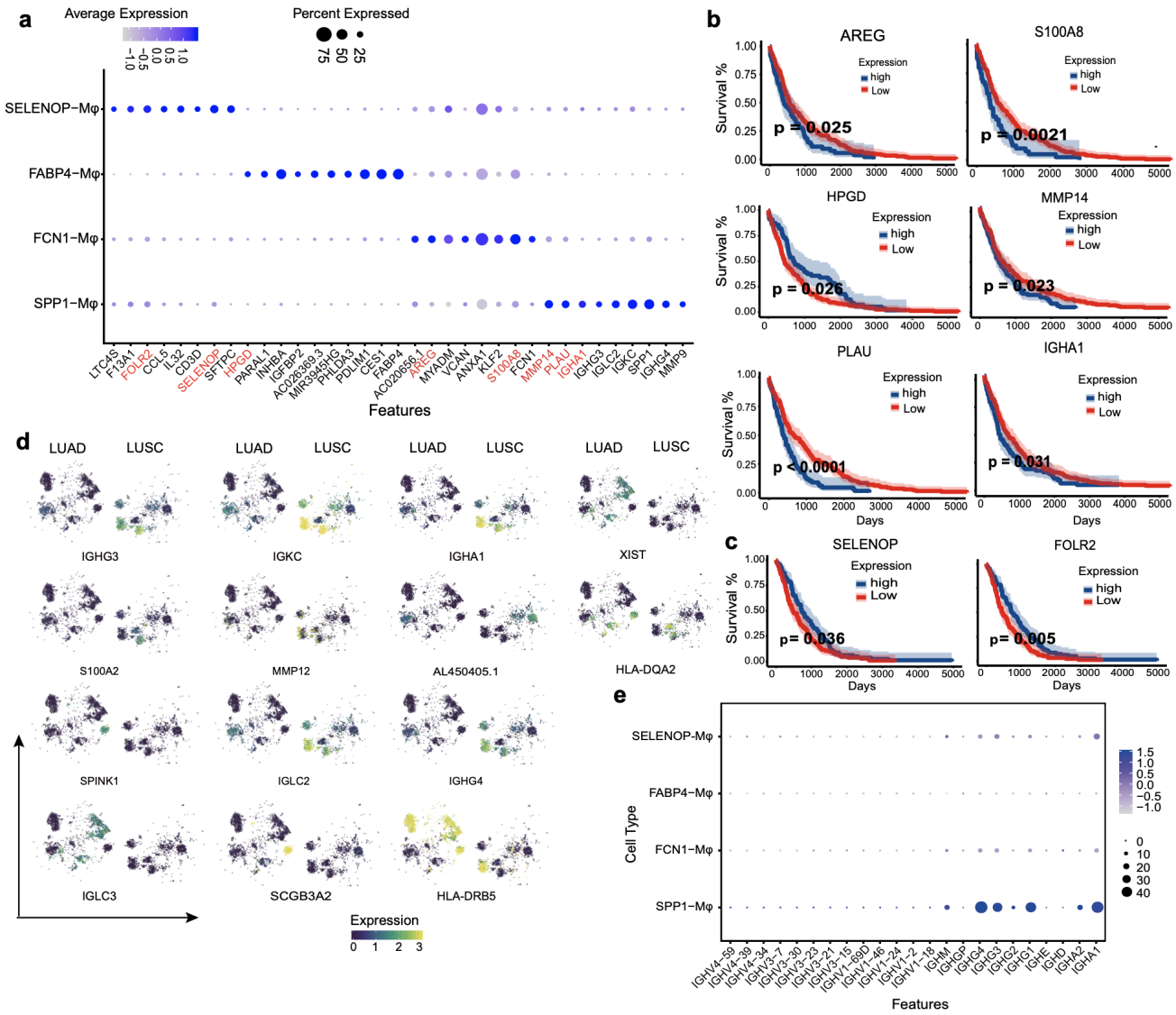
**

**Figure. S6. Re-clustering and gene expression patterns of macrophages in LUAD and LUSC.**

**a** Dot plot showing the expression of tumor-related genes in different macrophage subtypes. **b,c** Survival analyses of marker genes of macrophage subtypes in LUAD (b) and LUSC (c) based on TCGA database. **d** t-SNE showing the expression distribution of marker genes in LUAD and LUSC. **e** Dot-plot showing expression patterns of antibody transcripts among macrophages sub-clusters.


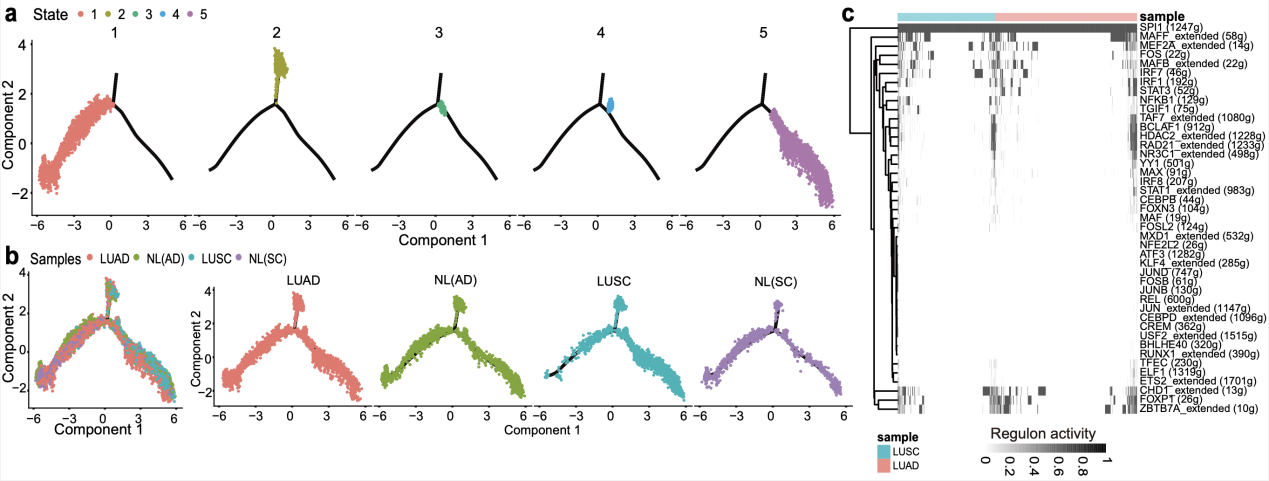


**Figure. S7. The trajectory analyses of macrophages in LUAD and LUSC revealed distinct features.**

**a,b** Pseudotime analyses of macrophages, colored by cell states (**a**) and sample origins (**b**). **c** Heatmap showing regulation heterogeneity of TF genes among macrophages in LUAD and LUSC by SCENIC.


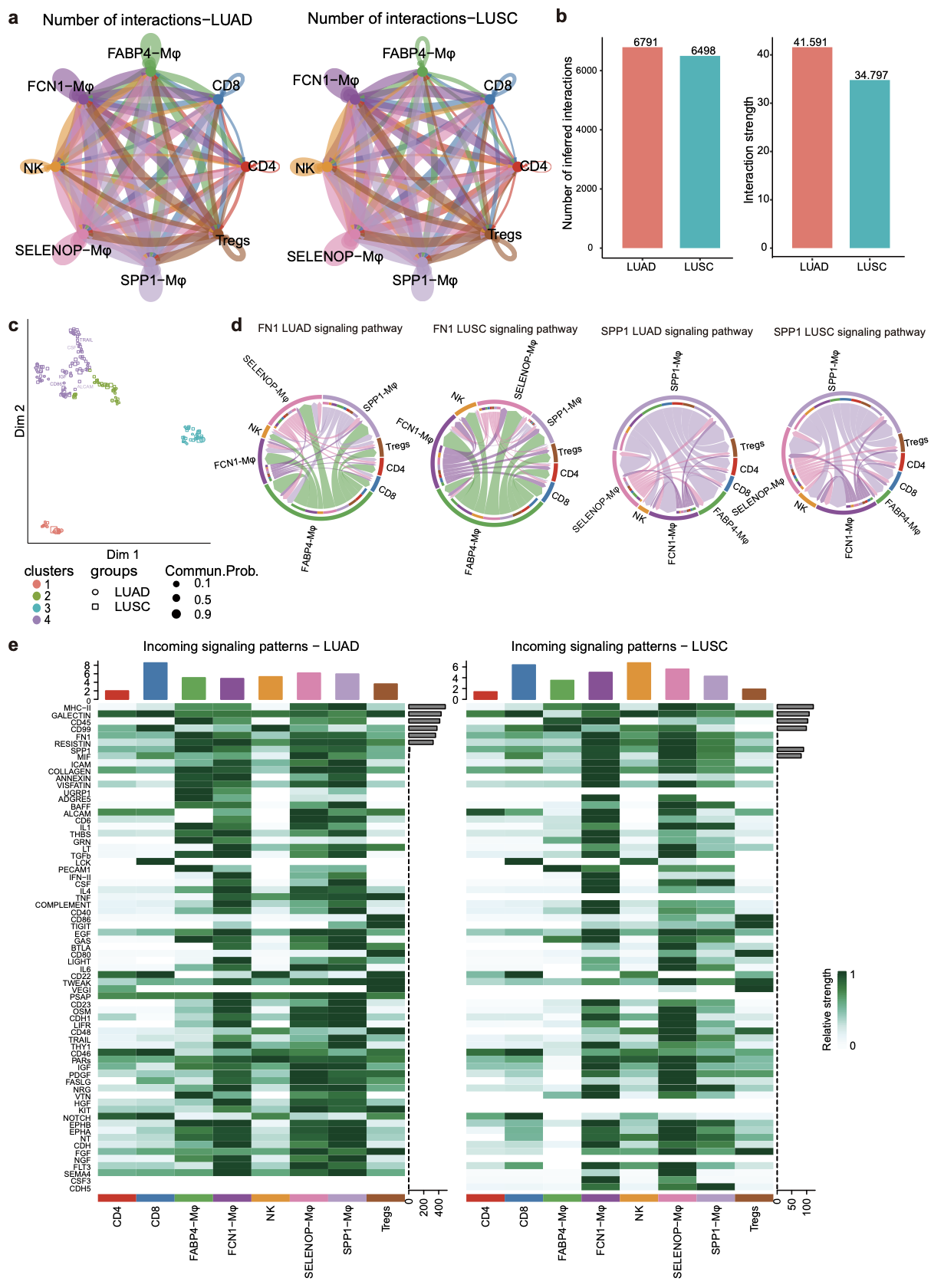


**Figure. S8. Distinct communication patterns between macrophages and lymphocytes in LUAD and LUSC.**

**a** Network plot summarizing interconnections between macrophages subtypes and lymphocytes from LUAD and LUSC. Lines represent potential interconnections between cell types, with line thickness proportional to the number of ligand-receptor pairs expressed in the connected cell types. **b** Bar plot showing the number and strength of inferred interactions in all these cells of LUAD and LUSC. **c** A two-dimensional manifold of differential signaling pathways between LUAD and LUSC according to their functional similarity. Each dot represents the communication network of one signaling pathway. Dot size is proportional to the overall communication probability. Different colors represent different groups of signaling pathways. **d** Chord plot showing inferred intercellular communication network of *FN1* and *SPP1* signaling in LUAD and LUSC. Inner and outer bars indicate autocrine and paracrine signaling to macrophages subtypes and lymphocytes, respectively. Bar size is proportional to the number of cells in each cell group and line width represents the communication probability. **e** Heat map showing the significant incoming signaling pathways of LUAD and LUSC among macrophages subtypes and lymphocytes. Color represents the communication probability.

**Table S1. The clinical characteristics of patients in our study**

| **Sample ID** | **Patient ID** | **Disease** | **Age** | **Sex** | **Smoking Status** | **Stage** | **EGFR** | **KRAS** | **ALK** | **ROS-1** | **PD-L1** |
| --- | --- | --- | --- | --- | --- | --- | --- | --- | --- | --- | --- |
| **AK3274** | **PS01** | **LUSC** | **55** | **Male** | **Yes** | **II** | **unknown** |  | **-** | **-** | **70%** |
| **AK3714** | **PS01** | **NL(SC)** | **55** | **Male** |  |  |  |  |  |  |  |
| **AK3716** | **PS01** | **NL(SC)** | **55** | **Male** | **Yes** | **II** | **unknown** |  | **-** | **-** | **70%** |
| **AK4295** | **PS01** | **LUSC** | **55** | **Male** |  |  |  |  |  |  |  |
| **2018jz4** | **PS02** | **LUSC** | **48** | **Male** | **Yes** | **II** | **unknown** |  | **unknown** | **unknown** | **-** |
| **2018jz5** | **PS02** | **NL(SC)** | **48** | **Male** |  |  |  |  |  |  |  |
| **2018jz36** | **PS03** | **NL(SC)** | **64** | **Male** | **Yes** | **III** | **19Del** |  | **-** | **-** | **-** |
| **2018jz37** | **PS03** | **LUSC** | **64** | **Male** |  |  |  |  |  |  |  |
| **2018jz38** | **PS04** | **LUSC** | **61** | **Male** | **Yes** | **III** | **unknown** |  | **-** | **-** | **5%** |
| **2018jz39** | **PS04** | **NL(SC)** | **61** | **Male** |  |  |  |  |  |  |  |
| **2018jz11** | **PS05** | **LUSC** | **70** | **Male** | **Yes** | **I** | **unknown** |  | **unknown** | **unknown** | **70%** |
| **2018jz14** | **PS05** | **NL(SC)** | **70** | **Male** |  |  |  |  |  |  |  |
| **2018jz46** | **PS06** | **LUSC** | **53** | **Male** | **Yes** | **I** | **unknown** |  | **-** | **-** | **-** |
| **2018jz47** | **PS06** | **NL(SC)** | **53** | **Male** |  |  |  |  |  |  |  |
| **AK653** | **PS07** | **NL(SC)** | **80** | **Male** | **Yes** | **I** | **unknown** |  | **-** | **-** | **40%** |
| **AK658** | **PS07** | **LUSC** | **80** | **Male** |  |  |  |  |  |  |  |
| **AK2834** | **PS08** | **LUSC** | **51** | **Male** | **No** | **II** | **unknown** |  | **-** | **-** | **-** |
| **AK831** | **PS08** | **NL(SC)** | **51** | **Male** |  |  |  |  |  |  |  |
| **2018jz12** | **PS09** | **LUSC** | **76** | **Male** | **Yes** | **I** | **unknown** |  | **-** | **-** | **-** |
| **2018jz15** | **PS09** | **NL(SC)** | **76** | **Male** |  |  |  |  |  |  |  |
| **2018jz1** | **PA01** | **LUAD** | **73** | **Male** | **Yes** | **II** | **unknown** |  | **-** | **-** | **15%** |
| **2018jz2** | **PA01** | **NL(AD)** | **73** | **Male** |  | **I** |  |  |  |  |  |
| **2018jz54** | **PA02** | **LUAD** | **61** | **Male** | **Yes** | **I** | **unknown** |  | **-** | **+** | **-** |
| **2018jz63** | **PA02** | **NL(AD)** | **61** | **Male** |  |  |  |  |  |  |  |
| **2018jz18** | **PA03** | **NL(AD)** | **54** | **Female** | **No** | **I** | **unknown** |  | **-** | **-** | **-** |
| **2018jz19** | **PA03** | **LUAD** | **54** | **Female** |  |  |  |  |  |  |  |
| **2018jz33** | **PA04** | **LUAD** | **78** | **Female** | **No** | **I** | **unknown** |  | **-** | **-** | **-** |
| **2018jz34** | **PA04** | **NL(AD)** | **79** | **Female** |  |  |  |  |  |  |  |
| **2018jz40** | **PA05** | **NL(AD)** | **52** | **Male** | **Yes** | **III** | **unknown** |  | **-** | **-** | **-** |
| **2018jz41** | **PA05** | **LUAD** | **52** | **Male** |  |  |  |  |  |  |  |
| **AK4436** | **PA06** | **NL(AD)** | **60** | **Male** | **Yes** | **III** | **-** | **-** | **-** | **-** | **90%** |
| **AK4556** | **PA06** | **LUAD** | **60** | **Male** |  |  |  |  |  |  |  |
| **AK649** | **PA07** | **NL(AD)** | **55** | **Female** | **No** | **I** | **unknown** |  | **-** | **+** | **-** |
| **AK654** | **PA07** | **LUAD** | **55** | **Female** |  |  |  |  |  |  |  |
| **2018jz7** | **PA08** | **LUAD** | **49** | **Female** | **No** | **IV** | **unknown** |  | **-** | **+** | **-** |
| **2018jz8** | **PA08** | **NL(AD)** | **49** | **Female** |  |  |  |  |  |  |  |
| **2018jz21** | **PA09** | **LUAD** | **53** | **Male** | **Yes** | **III** | **-** | **+** | **-** | **-** | **80%** |
| **2018jz22** | **PA09** | **NL(AD)** | **53** | **Male** |  |  |  |  |  |  |  |
| **2018jz25** | **PA10** | **NL(AD)** | **59** | **Female** | **No** | **IV** | **-** | **+** | **-** | **-** | **5%** |
| **2018jz26** | **PA10** | **LUAD** | **59** | **Female** |  |  |  |  |  |  |  |

**Table S2. The sequencing metrics for each sample**

| **Sample**  **ID** | **Patient ID** | **Estimated Number of Cells** | **Mean Reads per Cell** | **Median Genes per Cell** | **Total Genes Detected** | **Median UMI Counts per Cell** | **Number of Reads** | **Q30 Bases in Barcode** | **Q30 Bases in RNA Read** | **Q30 Bases in UMI** |
| --- | --- | --- | --- | --- | --- | --- | --- | --- | --- | --- |
| AK3274 | PS01 | 5444 | 7950 | 1396 | 22933 | 3675 | 43282580 | 0.971 | 0.864 | 0.978 |
| AK3714 | PS01 | 427 | 108312 | 784 | 15987 | 1699 | 46249531 | 0.965 | 0.864 | 0.966 |
| AK3716 | PS01 | 425 | 123519 | 798 | 16070 | 1730 | 52495660 | 0.965 | 0.864 | 0.966 |
| AK4295 | PS01 | 833 | 59973 | 751 | 17227 | 1827 | 49957699 | 0.965 | 0.866 | 0.965 |
| 2018jz4 | PS02 | 2617 | 111664 | 1114 | 23322 | 3087 | 292226410 | 0.964 | 0.907 | 0.962 |
| 2018jz5 | PS02 | 4816 | 50577 | 794 | 24407 | 1879 | 243580801 | 0.963 | 0.907 | 0.962 |
| 2018jz36 | PS03 | 1966 | 72554 | 834 | 18829 | 1958 | 142641483 | 0.963 | 0.91 | 0.961 |
| 2018jz37 | PS03 | 4503 | 4503 | 1125 | 25968 | 3599 | 287613871 | 0.962 | 0.908 | 0.961 |
| 2018jz38 | PS04 | 2802 | 89595 | 1286 | 23894 | 3748 | 251045458 | 0.964 | 0.91 | 0.962 |
| 2018jz39 | PS04 | 1386 | 139288 | 1000 | 19311 | 2434 | 193054214 | 0.963 | 0.906 | 0.961 |
| 2018jz11 | PS05 | 4598 | 52774 | 999 | 23827 | 2549 | 242657790 | 0.963 | 0.9 | 0.961 |
| 2018jz14 | PS05 | 3002 | 62427 | 1139 | 22931 | 3302 | 187406731 | 0.964 | 0.913 | 0.962 |
| 2018jz46 | PS06 | 1852 | 86236 | 998 | 23690 | 2905 | 159709817 | 0.961 | 0.905 | 0.959 |
| 2018jz47 | PS06 | 1766 | 88751 | 1140 | 21663 | 2989 | 156734501 | 0.962 | 0.907 | 0.96 |
| AK653 | PS07 | 1994 | 38048 | 956 | 21469 | 2176 | 75868356 | 0.97 | 0.876 | 0.974 |
| AK658 | PS07 | 4035 | 11868 | 678 | 21987 | 1420 | 47889114 | 0.971 | 0.879 | 0.975 |
| AK2834 | PS08 | 5757 | 17172 | 653 | 23758 | 1396 | 98861117 | 0.968 | 0.879 | 0.972 |
| AK831 | PS08 | 3565 | 15712 | 838 | 20955 | 1836 | 56013989 | 0.969 | 0.863 | 0.972 |
| 2018jz12 | PS09 | 5169 | 71775 | 1170 | 24699 | 2856 | 371007385 | 0.962 | 0.903 | 0.96 |
| 2018jz15 | PS09 | 4430 | 40944 | 1121 | 21881 | 2770 | 181383391 | 0.963 | 0.913 | 0.962 |
| 2018jz1 | PA01 | 1159 | 158322 | 1806 | 21917 | 6011 | 183495312 | 0.964 | 0.904 | 0.962 |
| 2018jz2 | PA01 | 2320 | 67303 | 1294 | 23394 | 4030 | 156144512 | 0.963 | 0.909 | 0.961 |
| 2018jz54 | PA02 | 5941 | 32631 | 1727 | 25784 | 5151 | 193861327 | 0.962 | 0.912 | 0.96 |
| 2018jz63 | PA02 | 2098 | 69653 | 1270 | 21861 | 3178 | 146132116 | 0.963 | 0.908 | 0.961 |
| 2018jz18 | PA03 | 5513 | 34534 | 830 | 20827 | 1932 | 190388300 | 0.963 | 0.917 | 0.962 |
| 2018jz19 | PA03 | 3512 | 49934 | 906 | 20216 | 2225 | 175368585 | 0.963 | 0.912 | 0.962 |
| 2018jz33 | PA04 | 2289 | 104129 | 1134 | 22794 | 3353 | 238352627 | 0.964 | 0.91 | 0.962 |
| 2018jz34 | PA04 | 2138 | 83286 | 1038 | 21889 | 2504 | 178065751 | 0.963 | 0.911 | 0.962 |
| 2018jz40 | PA05 | 1960 | 85119 | 300 | 18019 | 488 | 166834101 | 0.964 | 0.91 | 0.962 |
| 2018jz41 | PA05 | 3034 | 51971 | 950 | 20055 | 2400 | 157680355 | 0.963 | 0.91 | 0.961 |
| AK4436 | PA06 | 2481 | 30514 | 1012 | 22249 | 2611 | 75706521 | 0.972 | 0.885 | 0.975 |
| AK4556 | PA06 | 3174 | 19939 | 805 | 19672 | 1938 | 63289030 | 0.972 | 0.878 | 0.975 |
| AK649 | PA07 | 3599 | 9185 | 944 | 22776 | 2189 | 33056872 | 0.976 | 0.854 | 0.972 |
| AK654 | PA07 | 3360 | 16680 | 1306 | 23732 | 3547 | 56045489 | 0.976 | 0.886 | 0.966 |
| 2018jz7 | PA08 | 2254 | 75398 | 897 | 21322 | 2215 | 169948588 | 0.963 | 0.9 | 0.962 |
| 2018jz8 | PA08 | 2985 | 53868 | 739 | 21156 | 1644 | 160798561 | 0.964 | 0.902 | 0.963 |
| 2018jz21 | PA09 | 589 | 298952 | 1531 | 18064 | 4637 | 176082949 | 0.964 | 0.917 | 0.963 |
| 2018jz22 | PA09 | 335 | 503962 | 1432 | 16996 | 4374 | 168827467 | 0.962 | 0.912 | 0.96 |
| 2018jz25 | PA10 | 2227 | 206697 | 1641 | 23789 | 4998 | 460315320 | 0.962 | 0.903 | 0.96 |
| 2018jz26 | PA10 | 377 | 748788 | 1024 | 16280 | 2655 | 282293187 | 0.963 | 0.908 | 0.962 |

**Table S3. The top 20 differentially expressed genes for each cell cluster**

| **Cluster** | **Gene_name** | **Cluster B** | **Cluster CD4** | **Cluster CD8** | **Cluster DC** | **Cluster Gran** | **Cluster Mast** | **Cluster Mo** | **Cluster NK** | **Cluster Tregs** |
| --- | --- | --- | --- | --- | --- | --- | --- | --- | --- | --- |
| **B** | VPREB3 | 2.391952989 | 0.006391737 | 0.003178461 | 0.002892781 | 0.002268025 | 0.004392808 | 0.002872997 | 0.002971989 | 0.003379816 |
| **B** | IGHG2 | 39.55267955 | 0.072523231 | 0.1331265 | 0.038560924 | 0.086337715 | 0.232823996 | 0.110849402 | 0.04825291 | 0.217301444 |
| **B** | IGLC3 | 169.4177632 | 0.660909348 | 0.799808664 | 0.189772754 | 0.218433292 | 0.825130344 | 0.357795694 | 0.320934054 | 1.237426743 |
| **B** | IGHG1 | 241.7412152 | 0.637479384 | 1.022594253 | 0.36615895 | 0.675713078 | 2.966259419 | 0.80159541 | 0.458939586 | 2.001859995 |
| **B** | CD19 | 1.197717047 | 0.002716222 | 0.003864867 | 0.008461246 | 0.00589226 | 0.003702332 | 0.005981192 | 0.00280405 | 0 |
| **B** | IGLC2 | 464.5967377 | 1.81903565 | 2.284409492 | 0.628782925 | 1.1665799 | 3.681053313 | 1.428128326 | 0.921903357 | 3.470910174 |
| **B** | FCRL5 | 0.903175896 | 0.008666114 | 0.00215261 | 0 | 0.001290589 | 0.006544011 | 0.00138609 | 0.001887463 | 0.003621579 |
| **B** | IGKC | 1177.153624 | 4.71650432 | 6.087750306 | 2.410270047 | 3.85414126 | 10.44890403 | 4.588131427 | 2.834994574 | 11.24200034 |
| **B** | IGHA1 | 193.0614344 | 0.963217442 | 1.116266449 | 0.485496593 | 0.752657492 | 1.7263926 | 1.001849101 | 0.570703154 | 1.741472198 |
| **B** | FCRLA | 1.131742291 | 0.01523225 | 0.001090005 | 0.003199484 | 0.004542649 | 0.002325722 | 0.004123322 | 0.002587678 | 0.000997415 |
| **B** | IGHG3 | 195.4784625 | 0.80797475 | 1.16126076 | 0.428231894 | 0.762899436 | 3.622556821 | 0.823956502 | 0.719330454 | 2.060200252 |
| **B** | IGHM | 54.25213867 | 0.311488666 | 0.327670318 | 0.109482056 | 0.180338798 | 0.284588634 | 0.271786329 | 0.209719619 | 0.497629439 |
| **B** | IGHA2 | 40.583022 | 0.255060611 | 0.269641982 | 0.088492908 | 0.10795812 | 0.202897669 | 0.237348495 | 0.167301199 | 0.375795005 |
| **B** | CD79A | 8.398418144 | 0.041206509 | 0.029480638 | 0.018380149 | 0.018715765 | 0.062698241 | 0.013182928 | 0.033410036 | 0.280632344 |
| **B** | IGHG4 | 274.0323764 | 1.14290858 | 1.735121341 | 0.772849833 | 1.411951014 | 4.822150941 | 1.765014174 | 0.747717796 | 3.856185036 |
| **B** | MS4A1 | 6.947731568 | 0.049223802 | 0.151034154 | 0.006823126 | 0.013271014 | 0.020010588 | 0.012227771 | 0.036971463 | 0.025449743 |
| **B** | JCHAIN | 66.19668294 | 1.646406564 | 0.270045183 | 0.152105589 | 0.173620413 | 0.296552879 | 0.186580129 | 0.177997308 | 0.336923666 |
| **B** | TNFRSF17 | 0.539056913 | 0.013525226 | 0.001866427 | 0.001317035 | 0.001544979 | 0.003953162 | 0.001101179 | 0.002575173 | 0.003186971 |
| **B** | BANK1 | 2.049191537 | 0.01100588 | 0.003757986 | 0.123770598 | 0.048158245 | 0.011628855 | 0.041869229 | 0.004843394 | 0.003270846 |
| **B** | TNFRSF13C | 1.744484984 | 0.042379104 | 0.037641528 | 0.006860848 | 0.0045347 | 0.001184144 | 0.002240119 | 0.020472475 | 0.02056123 |
| **CD4** | IL7R | 0.134836961 | 6.812444447 | 2.012485538 | 1.864819954 | 0.377867638 | 0.15612186 | 1.333056503 | 1.148292865 | 3.610420427 |
| **CD4** | TRAT1 | 0.029946956 | 1.68837213 | 1.451658928 | 0.08159923 | 0.022453965 | 0.049508147 | 0.024299287 | 0.446123492 | 0.901475252 |
| **CD4** | LTB | 6.912517852 | 12.44749774 | 3.659438244 | 3.662644952 | 0.513271407 | 0.449414701 | 0.432968637 | 1.285705574 | 21.47252446 |
| **CD4** | LINC00513 | 0.38408787 | 1.498386918 | 1.252759765 | 0.384421776 | 0.163515366 | 0.194817982 | 0.10321268 | 0.570866184 | 1.549635937 |
| **CD4** | AC016831.5 | 0.880530696 | 3.316775779 | 2.794588247 | 0.992533102 | 0.460870521 | 0.371256301 | 0.348703331 | 1.322236247 | 2.920524289 |
| **CD4** | CD6 | 0.070308929 | 1.933336474 | 1.585134948 | 0.173819455 | 0.028396004 | 0.02418072 | 0.064283466 | 1.122937491 | 1.697548984 |
| **CD4** | AC058791.1 | 0.80233594 | 2.982314317 | 2.647598389 | 0.780604395 | 0.408329074 | 0.355715667 | 0.25464619 | 1.181410644 | 2.93998935 |
| **CD4** | PBXIP1 | 0.365652409 | 1.874107721 | 1.203170129 | 0.240244607 | 0.106986834 | 0.422476246 | 0.138332064 | 0.832376889 | 3.10143027 |
| **CD4** | PIK3IP1 | 0.574014504 | 2.087950056 | 1.459729694 | 0.201931403 | 0.092037789 | 0.271880581 | 0.210692511 | 1.288716001 | 2.211745148 |
| **CD4** | OCIAD2 | 0.752105815 | 1.976235126 | 1.833322202 | 0.252942997 | 0.064250816 | 0.736547812 | 0.140052295 | 0.977056206 | 2.385641006 |
| **CD4** | ICOS | 0.059192506 | 1.828022262 | 1.323268618 | 0.124207384 | 0.021384945 | 0.120091871 | 0.040961332 | 0.566822447 | 5.976980856 |
| **CD4** | NR3C1 | 0.491467135 | 2.256460536 | 1.375707377 | 0.749177473 | 0.549510618 | 0.680365431 | 0.590663968 | 1.000719907 | 2.756836656 |
| **CD4** | LEPROTL1 | 0.457897103 | 4.449545643 | 4.107358457 | 0.80699163 | 0.90335247 | 1.567214585 | 1.117003388 | 2.141854236 | 3.332655914 |
| **CD4** | LDHB | 1.52179816 | 5.064304311 | 3.128386533 | 1.662539187 | 1.109863516 | 3.412607388 | 1.398300951 | 2.432958979 | 4.384236091 |
| **CD4** | TRAC | 0.935858419 | 11.98529086 | 13.28424891 | 0.79010784 | 0.169879665 | 0.640192268 | 0.253357534 | 6.051068209 | 22.9153172 |
| **CD4** | RORA | 0.219785309 | 2.825547541 | 1.884062858 | 0.234410237 | 0.062868363 | 0.181770595 | 0.102980949 | 2.30170753 | 3.077390287 |
| **CD4** | SPOCK2 | 0.264735253 | 2.439924398 | 1.479476256 | 0.202472228 | 0.044303329 | 0.074777227 | 0.060583684 | 1.65760033 | 5.316873283 |
| **CD4** | TMEM123 | 0.977593397 | 2.536623613 | 1.061965983 | 1.665270149 | 1.438071767 | 0.637171589 | 1.507088971 | 0.944853798 | 1.895011749 |
| **CD4** | EML4 | 0.646775277 | 2.037091632 | 1.792886434 | 0.492790359 | 0.508984797 | 0.429745544 | 0.959979068 | 0.927949499 | 1.725486178 |
| **CD4** | SARAF | 3.029773731 | 10.91974753 | 8.848245158 | 4.442786841 | 3.35566844 | 4.949546818 | 3.324132534 | 5.828886211 | 8.901493465 |
| **CD8** | GZMK | 0.118589836 | 2.186451333 | 12.50983742 | 0.191951937 | 0.066709695 | 0.313878059 | 0.06612483 | 2.038756941 | 0.631501383 |
| **CD8** | CD8B | 0.063211336 | 0.438657347 | 4.505066418 | 0.10846124 | 0.03104409 | 0.083909003 | 0.027583536 | 1.656563959 | 0.210993394 |
| **CD8** | ITM2C | 1.556511093 | 0.827586205 | 3.004415667 | 0.288815223 | 0.037395513 | 1.594920009 | 0.073711219 | 0.486335891 | 0.353763705 |
| **CD8** | CD8A | 0.081232266 | 0.457020886 | 5.273387635 | 0.133774902 | 0.042104209 | 0.094212618 | 0.039113607 | 2.292772299 | 0.254362956 |
| **CD8** | CCL4L2 | 0.364377845 | 1.009709596 | 34.74084867 | 3.124638362 | 2.566317384 | 2.129293633 | 4.963487633 | 13.09593103 | 0.927910839 |
| **CD8** | IFNG | 0.155348809 | 1.320310311 | 7.337025106 | 0.184461875 | 0.082441338 | 0.191065832 | 0.074054747 | 4.330046938 | 1.146393589 |
| **CD8** | LAG3 | 0.083338906 | 0.349959008 | 2.625330919 | 0.235687394 | 0.033929365 | 0.033679268 | 0.11420107 | 1.471948803 | 1.532348374 |
| **CD8** | TRGC2 | 0.065182357 | 0.349976652 | 3.040316442 | 0.108544894 | 0.058054366 | 0.070438419 | 0.083035159 | 2.287596671 | 0.166064819 |
| **CD8** | CCL5 | 0.955353612 | 10.37690049 | 59.8615591 | 1.704534797 | 0.722021957 | 1.377771205 | 0.717746136 | 47.08257847 | 3.56571888 |
| **CD8** | CCL4 | 0.958325369 | 4.643505079 | 55.52976303 | 3.692032992 | 5.289351653 | 2.782857215 | 9.139186927 | 45.64826651 | 3.576619514 |
| **CD8** | SH2D1A | 0.042029473 | 0.893178836 | 2.185923261 | 0.088710299 | 0.02724299 | 0.065917685 | 0.035957161 | 1.348388857 | 1.713046673 |
| **CD8** | CD96 | 0.193568811 | 1.504155642 | 2.544056388 | 0.149627867 | 0.040543984 | 0.072716938 | 0.053276861 | 1.397644534 | 1.698572164 |
| **CD8** | APOBEC3G | 0.471915747 | 1.005813189 | 3.21752246 | 0.343764579 | 0.182611261 | 0.215908156 | 0.181462805 | 2.391805952 | 1.296324009 |
| **CD8** | CLDND1 | 0.422453711 | 1.085048881 | 2.087611986 | 0.514154904 | 0.443136925 | 0.59100519 | 0.363176604 | 0.858035981 | 1.248970715 |
| **CD8** | TUBA4A | 0.98346992 | 2.025113653 | 4.421722933 | 0.316148901 | 0.38019495 | 0.184135339 | 0.129951982 | 3.032313821 | 1.256415529 |
| **CD8** | GZMA | 0.273833657 | 3.663120458 | 17.14166573 | 0.547830799 | 0.192990768 | 0.697883783 | 0.191868967 | 17.37805254 | 1.795460842 |
| **CD8** | DUSP4 | 1.187631673 | 2.516685456 | 4.839767673 | 2.654797285 | 0.286782461 | 0.436050294 | 0.212736489 | 1.566035371 | 9.466645215 |
| **CD8** | CD3D | 0.322560783 | 8.755584506 | 13.09735562 | 0.686256786 | 0.182468396 | 0.610436871 | 0.254322129 | 7.31771283 | 14.85037976 |
| **CD8** | CD27 | 1.593218828 | 0.937499271 | 1.938960528 | 0.120749197 | 0.017163651 | 0.127992226 | 0.041747076 | 0.617235342 | 4.971744983 |
| **CD8** | ITM2A | 0.318337172 | 3.187193742 | 4.160767754 | 0.20974967 | 0.065981547 | 2.24869657 | 0.070889629 | 2.117867574 | 4.946153875 |
| **DC** | CCL17 | 0.020943421 | 0.011151642 | 0.013174785 | 12.25439563 | 0.218253447 | 0.005188231 | 0.198166048 | 0.00924745 | 0.002950224 |
| **DC** | CD1E | 0.011553138 | 0.006950199 | 0.006769412 | 3.374007255 | 0.065253993 | 0.011859246 | 0.108064195 | 0.009162357 | 0.008583153 |
| **DC** | WFDC21P | 0.065886834 | 0.0216148 | 0.029045848 | 6.120270251 | 0.056357583 | 0.018878138 | 0.268015418 | 0.01856355 | 0.012291255 |
| **DC** | CD1C | 0.236818383 | 0.007625092 | 0.009875359 | 3.805744446 | 0.159544674 | 0.027437773 | 0.132602244 | 0.011445149 | 0.010088657 |
| **DC** | FCER1A | 0.012301747 | 0.061720566 | 0.016065725 | 4.279004003 | 0.199480304 | 2.050911919 | 0.109641656 | 0.010497022 | 0.005016948 |
| **DC** | PPP1R14A | 0.049355274 | 0.070188434 | 0.003585183 | 1.218049487 | 0.01830173 | 0.013645384 | 0.038156244 | 0.003438094 | 0.001635011 |
| **DC** | PKIB | 0.003683388 | 0.009106275 | 0.008817096 | 2.115122446 | 0.093464706 | 0.151456584 | 0.209214574 | 0.005479089 | 0.005118518 |
| **DC** | S100B | 0.017511036 | 0.044222192 | 0.051094324 | 5.212417865 | 0.065755545 | 0.024757243 | 0.452091965 | 0.3368995 | 0.021545767 |
| **DC** | NDRG2 | 0.016416649 | 0.051612138 | 0.032755113 | 1.274271691 | 0.062929395 | 0.368438076 | 0.225047466 | 0.034046197 | 0.026822814 |
| **DC** | CLEC10A | 0.007159348 | 0.008563277 | 0.012457543 | 3.322669819 | 0.768413186 | 0.029616936 | 0.610552097 | 0.010797266 | 0.013221023 |
| **DC** | AXL | 0.01382794 | 0.019343659 | 0.016268446 | 3.384792097 | 0.177604929 | 0.019953167 | 1.227283113 | 0.007566084 | 0.005592556 |
| **DC** | NAPSB | 1.551842642 | 0.339346527 | 0.022122464 | 4.620473124 | 0.810664262 | 0.030521858 | 0.480451446 | 0.020650537 | 0.009328501 |
| **DC** | CLIC2 | 0.012280558 | 0.010083319 | 0.00325964 | 0.710366164 | 0.077874564 | 0.027316676 | 0.222055813 | 0.006803738 | 0.007943828 |
| **DC** | FILIP1L | 0.015024718 | 0.013072322 | 0.004073735 | 0.888509522 | 0.05984063 | 0.051564807 | 0.318628186 | 0.004461077 | 0.032661247 |
| **DC** | CSF2RA | 0.007069093 | 0.042287984 | 0.008849956 | 2.562599349 | 0.626459883 | 0.024862137 | 0.837000101 | 0.008328257 | 0.00528 |
| **DC** | CD80 | 0.061391656 | 0.014449997 | 0.01178872 | 0.666720447 | 0.086057399 | 0.006570905 | 0.211221502 | 0.005840718 | 0.094428628 |
| **DC** | C1orf54 | 0.011520849 | 0.02229049 | 0.013535795 | 1.928361456 | 0.251471571 | 0.030301729 | 1.011972893 | 0.013924626 | 0.011193903 |
| **DC** | LGALS2 | 0.006731436 | 0.01450632 | 0.017067784 | 4.055649656 | 2.858937444 | 0.014665633 | 0.506622225 | 0.014429312 | 0.011954675 |
| **DC** | SERPINF1 | 0.064907073 | 0.302029503 | 0.02094264 | 1.616045497 | 0.102931725 | 0.280958914 | 0.533739888 | 0.016701834 | 0.030125544 |
| **DC** | CPVL | 0.009327513 | 0.026462281 | 0.021307164 | 4.72291307 | 2.386997066 | 0.03132256 | 1.409716045 | 0.022413288 | 0.016783587 |
| **Gran** | S100A12 | 0.004970583 | 0.009895284 | 0.014610007 | 0.075098829 | 8.83299105 | 0.031759303 | 0.173309345 | 0.013311876 | 0.002353205 |
| **Gran** | APOBEC3A | 0.008646364 | 0.010644952 | 0.005435612 | 0.057110551 | 2.571079355 | 0.004743479 | 0.089968219 | 0.009153768 | 0.005193921 |
| **Gran** | AC245128.3 | 0.006318897 | 0.003808946 | 0.000869745 | 0.012008613 | 1.114377303 | 0.010362738 | 0.057337915 | 0.002593624 | 0.001890059 |
| **Gran** | FCN1 | 0.014976683 | 0.027683851 | 0.034825102 | 0.601529055 | 12.5445989 | 0.076793147 | 1.057074883 | 0.043759911 | 0.019237451 |
| **Gran** | CD300E | 0.005731477 | 0.004538908 | 0.006878371 | 0.071787221 | 1.885263956 | 0.005529132 | 0.182122452 | 0.007286876 | 0.007577852 |
| **Gran** | LILRA5 | 0.001113921 | 0.029224202 | 0.001467503 | 0.168239576 | 1.572706976 | 0.010490276 | 0.141585772 | 0.00255414 | 0.001429206 |
| **Gran** | S100A8 | 0.240176458 | 0.204865241 | 0.23253067 | 1.04101003 | 57.73972856 | 0.366657761 | 8.96848702 | 0.194469295 | 0.179718615 |
| **Gran** | VCAN | 0.009316977 | 0.019536594 | 0.020100856 | 0.257509207 | 6.608795315 | 0.074134569 | 1.025933752 | 0.019314282 | 0.01693203 |
| **Gran** | S100A9 | 0.525119085 | 0.371397096 | 0.417576539 | 1.588356733 | 66.18646849 | 0.544536587 | 10.341168 | 0.291694077 | 0.416737781 |
| **Gran** | THBS1 | 0.017862914 | 0.039666129 | 0.072438326 | 0.268596261 | 5.549304363 | 0.201084417 | 0.752729036 | 0.053232807 | 0.017523058 |
| **Gran** | CFP | 0.013272875 | 0.071579257 | 0.012381599 | 0.864278584 | 2.845953365 | 0.010215353 | 0.179270764 | 0.012406396 | 0.051371872 |
| **Gran** | C19orf38 | 0.10186157 | 0.006085858 | 0.004186844 | 0.188905444 | 0.94122437 | 0.011735873 | 0.151659506 | 0.005941715 | 0.010184433 |
| **Gran** | EREG | 0.021805903 | 0.035326075 | 0.041719561 | 1.719477411 | 11.4550927 | 0.16420697 | 2.600359966 | 0.052590461 | 0.027753729 |
| **Gran** | CLEC4E | 0.005221987 | 0.007628799 | 0.001725472 | 0.109669346 | 1.244753888 | 0.017434295 | 0.320122602 | 0.003345052 | 0.001663704 |
| **Gran** | G0S2 | 0.217989291 | 0.115548165 | 0.099173716 | 5.672231679 | 19.92882478 | 0.365188902 | 4.781635902 | 0.102960182 | 0.29803553 |
| **Gran** | IL1B | 0.076202619 | 0.081276007 | 0.082076614 | 5.386280857 | 20.30801816 | 0.261188755 | 5.226076391 | 0.093293386 | 0.073956544 |
| **Gran** | NLRP3 | 0.013752273 | 0.056469388 | 0.042971938 | 0.74898928 | 2.387498323 | 0.077735434 | 0.477275525 | 0.022799612 | 0.023323439 |
| **Gran** | TIMP1 | 0.624423045 | 1.455432486 | 0.705156832 | 9.236224145 | 38.76536169 | 8.332539952 | 6.173716593 | 0.928928147 | 1.428609901 |
| **Gran** | LILRB2 | 0.010359201 | 0.026485406 | 0.005414274 | 0.330297546 | 1.682148896 | 0.019404829 | 0.509159865 | 0.009219024 | 0.005181728 |
| **Gran** | AQP9 | 0.009627766 | 0.007328224 | 0.010752849 | 0.023006675 | 1.355834791 | 0.023218053 | 0.537782351 | 0.009224401 | 0.005434243 |
| **Mast** | TPSAB1 | 0.074867702 | 0.043870703 | 0.089212768 | 0.021576551 | 0.01487859 | 189.709327 | 0.024265043 | 0.024514858 | 0.104538376 |
| **Mast** | MS4A2 | 0.001296847 | 0.001730729 | 0.011031642 | 0.000452421 | 0.003116473 | 12.09566852 | 0.00136692 | 0.001371404 | 0.010843985 |
| **Mast** | TPSB2 | 0.259956398 | 0.149810219 | 0.275264958 | 0.076889193 | 0.099228642 | 506.8465493 | 0.154539104 | 0.093343637 | 0.500614544 |
| **Mast** | CPA3 | 0.024528258 | 0.013265901 | 0.020770351 | 0.005547064 | 0.009909979 | 40.9841747 | 0.010695757 | 0.010449501 | 0.03383785 |
| **Mast** | LINC01835 | 0.00043228 | 0.000777059 | 0.000804851 | 0.000743783 | 0 | 1.310841146 | 0.00027269 | 0.000310028 | 0.001483217 |
| **Mast** | GATA2 | 0.007074231 | 0.0023904 | 0.007156956 | 0.002524659 | 0.005386193 | 10.51335037 | 0.004601429 | 0.001463975 | 0.011280415 |
| **Mast** | AL157895.1 | 0.002060779 | 0.001929679 | 0.002452384 | 0.001004862 | 0 | 4.180758348 | 0.001980024 | 0.002403208 | 0 |
| **Mast** | GCSAML | 0.002840436 | 0.000117944 | 0 | 0 | 0.002022483 | 1.258377146 | 0.000805209 | 0.000165018 | 0.000718672 |
| **Mast** | SLC18A2 | 0.003473105 | 0.007323253 | 0.009502569 | 0.033242204 | 0.001690978 | 9.79176858 | 0.002522506 | 0.001726035 | 0.004569877 |
| **Mast** | CTSG | 0.00137193 | 0.001526527 | 0.002266085 | 0.002473814 | 0.004616533 | 6.935196326 | 0.010330702 | 0.003455481 | 0.002615569 |
| **Mast** | SVOPL | 0 | 0.001859827 | 0.001209169 | 0 | 0.000117141 | 1.277365975 | 0.000930101 | 0.001451209 | 0 |
| **Mast** | KIT | 0.001108133 | 0.009601058 | 0.005868642 | 0.0113353 | 0.001094114 | 5.745631004 | 0.001967157 | 0.003920462 | 0.006462905 |
| **Mast** | HDC | 0.001611155 | 0.003892476 | 0.006213853 | 0.004029595 | 0.001854157 | 3.998875255 | 0.002508335 | 0.003282393 | 0.013659453 |
| **Mast** | MAOB | 0.003639387 | 0.004340383 | 0.003645939 | 0.000967947 | 0.000354863 | 3.319834735 | 0.002802248 | 0.007023335 | 0.004152277 |
| **Mast** | IL1RL1 | 0.002630311 | 0.004442063 | 0.00305823 | 0.002061125 | 0.001473022 | 2.922580284 | 0.001291886 | 0.00408539 | 0.066242913 |
| **Mast** | SLC45A3 | 0.018390782 | 0.001409765 | 0.001684785 | 0.007460841 | 0.004367344 | 1.85338997 | 0.013143886 | 0.000618826 | 0.000571277 |
| **Mast** | RHEX | 0.079672436 | 0.035684152 | 0.00370368 | 0.031011924 | 0.001342869 | 3.973727771 | 0.001206562 | 0.001799497 | 0 |
| **Mast** | CAVIN2 | 0.000771247 | 0.003084623 | 0.003390306 | 0.043072702 | 0.012495064 | 1.428249433 | 0.006173165 | 0.001262649 | 0 |
| **Mast** | HPGDS | 0.005771487 | 0.011273447 | 0.007498181 | 0.310605041 | 0.013045766 | 18.0475999 | 0.408264017 | 0.004189436 | 0.009502621 |
| **Mast** | RGS13 | 0.1527272 | 0.026472712 | 0.024222689 | 0.055815387 | 0.012731804 | 6.977533588 | 0.024461724 | 0.039846092 | 0.032367155 |
| **Mφ** | CCL18 | 0.034306405 | 0.073564398 | 0.09490368 | 0.113809977 | 0.118515241 | 0.093942375 | 13.07585686 | 0.068812389 | 0.044785463 |
| **Mφ** | LPL | 0.000541052 | 0.003994888 | 0.00376379 | 0.055028245 | 0.035753375 | 0.027003842 | 1.02327985 | 0.002355295 | 0.008015256 |
| **Mφ** | APOC1 | 0.24465798 | 0.257379045 | 0.327890814 | 2.183335862 | 1.14458765 | 5.259156081 | 50.93702506 | 0.214422794 | 0.243330391 |
| **Mφ** | NUPR1 | 0.054662081 | 0.048927193 | 0.058112485 | 0.064319213 | 0.116880098 | 0.10555522 | 4.708883019 | 0.033945038 | 0.045830717 |
| **Mφ** | APOE | 0.361862376 | 0.363067686 | 0.431701314 | 1.597508675 | 0.864576943 | 8.767101394 | 50.47938563 | 0.2419484 | 0.321099218 |
| **Mφ** | GPNMB | 0.051717935 | 0.042697923 | 0.055477018 | 0.285980139 | 0.28172138 | 0.122508265 | 6.249119156 | 0.024285659 | 0.046531555 |
| **Mφ** | RNASE1 | 0.040301977 | 0.073295126 | 0.06183202 | 0.113928023 | 0.287313769 | 0.144209012 | 6.363814151 | 0.046714783 | 0.030643658 |
| **Mφ** | SLCO2B1 | 0.006675479 | 0.00507434 | 0.009470046 | 0.191338484 | 0.092490646 | 0.119115858 | 1.481681997 | 0.004957609 | 0.003818657 |
| **Mφ** | FN1 | 0.032015808 | 0.055180889 | 0.052609971 | 0.356584558 | 0.938769796 | 0.185077008 | 8.922467394 | 0.040892434 | 0.079771402 |
| **Mφ** | TREM2 | 0.013987511 | 0.018270818 | 0.018379631 | 0.749259004 | 0.194996156 | 0.042847841 | 3.668613842 | 0.013945477 | 0.020644281 |
| **Mφ** | C1QB | 0.138128874 | 0.149862809 | 0.197919762 | 6.626330092 | 1.716386787 | 0.370106822 | 32.05772408 | 0.116134299 | 0.153219271 |
| **Mφ** | SERPING1 | 0.027895582 | 0.033957301 | 0.014643376 | 0.171160346 | 0.155356657 | 0.083317969 | 2.135855093 | 0.014769364 | 0.029714343 |
| **Mφ** | FOLR2 | 0.078029492 | 0.007325481 | 0.005975327 | 0.084906573 | 0.172657297 | 0.017815516 | 1.599225845 | 0.004831512 | 0.007605287 |
| **Mφ** | PLTP | 0.06706832 | 0.013262024 | 0.014393143 | 0.08128914 | 0.058344013 | 0.053091899 | 1.198001314 | 0.02064078 | 0.005320061 |
| **Mφ** | MARCO | 0.010499474 | 0.027572912 | 0.026551251 | 0.166283779 | 0.875571565 | 0.046265085 | 5.83062657 | 0.020665507 | 0.013904104 |
| **Mφ** | CYP27A1 | 0.0068089 | 0.006879397 | 0.009714919 | 0.051071641 | 0.168504741 | 0.023776117 | 1.255833992 | 0.00569383 | 0.007963426 |
| **Mφ** | C1QA | 0.137734048 | 0.155577173 | 0.20524187 | 6.968094253 | 2.574385809 | 0.371637314 | 33.52739231 | 0.113856538 | 0.166225247 |
| **Mφ** | KCNMA1 | 0.03832638 | 0.003778844 | 0.005597938 | 0.034245643 | 0.073312622 | 0.009012034 | 0.637700289 | 0.002279811 | 0.001013365 |
| **Mφ** | C2 | 0.021332845 | 0.00737124 | 0.010408673 | 0.041607575 | 0.090767713 | 0.05165315 | 0.795881826 | 0.004466572 | 0.007455503 |
| **Mφ** | MSR1 | 0.013592745 | 0.010727845 | 0.022543789 | 0.373122854 | 0.484246563 | 0.028399889 | 3.566734993 | 0.011362802 | 0.018534014 |
| **NK** | KLRF1 | 0.048038255 | 0.030250511 | 0.056567815 | 0.051545163 | 0.054003368 | 0.059844189 | 0.03337619 | 4.642825979 | 0.010924386 |
| **NK** | FGFBP2 | 0.083401024 | 0.060608005 | 0.297641312 | 0.106789116 | 0.091486461 | 0.080773541 | 0.054384057 | 10.84205583 | 0.03083301 |
| **NK** | S1PR5 | 0.016186298 | 0.015676443 | 0.066061536 | 0.01736776 | 0.01751253 | 0.01201848 | 0.007679319 | 1.97696376 | 0.00461191 |
| **NK** | SPON2 | 0.066948377 | 0.224722441 | 0.368679203 | 0.095489298 | 0.083338431 | 0.042685315 | 0.045170412 | 5.804382341 | 0.107329272 |
| **NK** | ADGRG1 | 0.011707992 | 0.026642412 | 0.105962173 | 0.048772805 | 0.028400735 | 0.023102266 | 0.024642702 | 1.594615315 | 0.087757549 |
| **NK** | GNLY | 0.513129383 | 0.809629501 | 9.047407738 | 0.656861188 | 0.615956466 | 0.583498382 | 0.329010151 | 55.57806629 | 1.193088205 |
| **NK** | FCRL6 | 0.015112639 | 0.03933326 | 0.306794357 | 0.045252283 | 0.011156139 | 0 | 0.006307693 | 1.398273728 | 0.015444356 |
| **NK** | KLRD1 | 0.09112424 | 0.101735797 | 2.311429236 | 0.135574127 | 0.117901616 | 0.121379336 | 0.066056968 | 9.25381207 | 0.077703365 |
| **NK** | TRDC | 0.062818149 | 0.156989308 | 1.029214659 | 0.037353698 | 0.041297015 | 0.060363236 | 0.019371129 | 4.121601107 | 0.133737015 |
| **NK** | PRF1 | 0.111968201 | 0.463907858 | 3.10807676 | 0.169487371 | 0.09344428 | 0.133922089 | 0.055599359 | 12.44509028 | 0.856749643 |
| **NK** | NKG7 | 0.607569198 | 1.27482577 | 18.31217392 | 0.994794281 | 0.82377331 | 0.795089991 | 0.441795317 | 51.35416945 | 0.691212369 |
| **NK** | CLIC3 | 0.110099967 | 0.491799369 | 0.821192585 | 0.182393546 | 0.048227026 | 0.090333601 | 0.027776755 | 3.087878797 | 0.142826636 |
| **NK** | CTSW | 0.204772026 | 0.659817072 | 4.613938326 | 0.237180044 | 0.098599572 | 1.425006146 | 0.127119844 | 9.503421242 | 0.24849147 |
| **NK** | GZMH | 0.160547903 | 0.77880897 | 8.897109328 | 0.31860957 | 0.149054909 | 0.247359813 | 0.102816785 | 14.08535598 | 0.355059807 |
| **NK** | CMC1 | 0.203562245 | 0.501205237 | 3.46284066 | 0.378577927 | 0.285737828 | 0.52404194 | 0.474587803 | 6.506211844 | 0.463710433 |
| **NK** | GZMB | 0.491030037 | 3.854107707 | 12.89740935 | 0.900735373 | 0.236732552 | 0.261211385 | 0.207323631 | 22.52005571 | 1.267498919 |
| **NK** | LAIR2 | 0.021862973 | 0.192998963 | 0.157925989 | 0.054258188 | 0.015458876 | 0.033883123 | 0.020743624 | 1.802628791 | 2.723053042 |
| **NK** | CD247 | 0.114588129 | 1.376335961 | 1.750020909 | 0.126888447 | 0.062905526 | 0.088856289 | 0.047661054 | 5.086321263 | 2.596883493 |
| **NK** | CST7 | 0.228051322 | 1.993029637 | 8.711073687 | 3.5971556 | 0.257042826 | 1.261658788 | 0.294816739 | 13.97457649 | 3.370482721 |
| **NK** | MATK | 0.042507028 | 0.284438485 | 1.151467969 | 0.143294723 | 0.064191649 | 0.344337863 | 0.215035428 | 1.806450479 | 0.057935948 |
| **Tregs** | FOXP3 | 0.006137476 | 0.075526045 | 0.012138702 | 0.030065076 | 0.003515208 | 0.034407881 | 0.009351103 | 0.023179421 | 1.643748922 |
| **Tregs** | TNFRSF4 | 0.096909723 | 0.780851218 | 0.131439131 | 0.306154725 | 0.052145096 | 0.97290855 | 0.105043111 | 0.184137751 | 10.25657282 |
| **Tregs** | IL2RA | 0.081269574 | 0.248426692 | 0.048935532 | 0.108483554 | 0.026974605 | 0.068857359 | 0.186446462 | 0.091437667 | 3.783521903 |
| **Tregs** | ICA1 | 0.018143213 | 0.112400365 | 0.035183713 | 0.074551128 | 0.023935438 | 0.159490889 | 0.078744021 | 0.053930781 | 1.739543378 |
| **Tregs** | MAGEH1 | 0.123129808 | 0.203683924 | 0.140916921 | 0.072376082 | 0.033744178 | 0.212336411 | 0.051136297 | 0.186170934 | 2.869419256 |
| **Tregs** | TNFRSF18 | 0.149164964 | 0.806937564 | 0.447031921 | 0.290329603 | 0.046006877 | 0.81649392 | 0.084113753 | 0.719464002 | 7.743102627 |
| **Tregs** | CTLA4 | 0.015416809 | 0.383460363 | 0.229019014 | 0.09232248 | 0.008868093 | 0.039702423 | 0.029133997 | 0.187895488 | 2.375102062 |
| **Tregs** | TBC1D4 | 0.025141127 | 0.355319442 | 0.119694087 | 0.362919354 | 0.034898432 | 0.148374952 | 0.184118161 | 0.126978152 | 2.208177363 |
| **Tregs** | LINC01943 | 0.045995734 | 0.854412162 | 0.445703606 | 0.126972802 | 0.096564757 | 0.03319556 | 0.072699457 | 0.283202602 | 3.749519567 |
| **Tregs** | TNFRSF9 | 0.011136961 | 0.076460917 | 0.307814349 | 0.131785246 | 0.020206793 | 0.765385432 | 0.037855468 | 0.364561205 | 2.093164354 |
| **Tregs** | BATF | 0.14472685 | 1.037349058 | 0.725297868 | 0.221337517 | 0.215192073 | 0.775097192 | 0.187842564 | 0.671877155 | 5.286561492 |
| **Tregs** | ICOS | 0.059192506 | 1.828022262 | 1.323268618 | 0.124207384 | 0.021384945 | 0.120091871 | 0.040961332 | 0.566822447 | 5.976980856 |
| **Tregs** | TIGIT | 0.084543661 | 0.603227964 | 1.417867245 | 0.102619241 | 0.023238822 | 0.183420617 | 0.038420932 | 1.491423465 | 6.215026169 |
| **Tregs** | CD27 | 1.593218828 | 0.937499271 | 1.938960528 | 0.120749197 | 0.017163651 | 0.127992226 | 0.041747076 | 0.617235342 | 4.971744983 |
| **Tregs** | SIRPG | 0.020294427 | 0.266990887 | 0.500761896 | 0.030657544 | 0.006605264 | 0.029722272 | 0.008239169 | 0.322081245 | 1.51198928 |
| **Tregs** | DNPH1 | 0.483469611 | 0.717530704 | 0.644022582 | 0.49033439 | 0.323702504 | 0.571077797 | 0.736420507 | 0.350647303 | 3.029356417 |
| **Tregs** | LTB | 6.912517852 | 12.44749774 | 3.659438244 | 3.662644952 | 0.513271407 | 0.449414701 | 0.432968637 | 1.285705574 | 21.47252446 |
| **Tregs** | GADD45A | 0.624155848 | 0.83371456 | 0.669788802 | 0.811205818 | 0.277748417 | 0.923805111 | 0.240604906 | 0.683884121 | 3.292335653 |
| **Tregs** | TNFRSF25 | 0.040503507 | 1.191599326 | 0.328636509 | 0.112947958 | 0.024395794 | 0.091521723 | 0.031638711 | 0.277963629 | 1.972261301 |
| **Tregs** | DUSP4 | 1.187631673 | 2.516685456 | 4.839767673 | 2.654797285 | 0.286782461 | 0.436050294 | 0.212736489 | 1.566035371 | 9.466645215 |

**Table S4. The average expression matrix of marker genes in CD4+ T cell subtypes**

|  |  | **GeneName** | **Cluster 0** | **Cluster 5** | **Cluster 7** | **Cluster 9** | **Cluster 1** | **Cluster 4** | **Cluster 6** | **Cluster 2** | **Cluster 3** | **Cluster 8** | **Cluster 10** | **Cluster 11** | **Cluster 12** |
| --- | --- | --- | --- | --- | --- | --- | --- | --- | --- | --- | --- | --- | --- | --- | --- |
|  |  |  | **Na**ї**ve T cell** | **Na**ї**ve T cell** | **Na**ї**ve T cell** | **Na**ї**ve T cell** | **Effector** | **Effector** | **Effector** | **Tregs** | **Tregs** | **exhausted** | **memory T cell** | **activated** | **γδT** |
|  |  | PTPRC | 6.8907559 | 5.3269303 | 6.1269921 | 6.6012266 | 8.2596834 | 7.348678773 | 9.2357856 | 7.6255377 | 7.301387768 | 1.4740689 | 6.847823346 | 6.6268958 | 3.8137807 |
|  | CD3E+ | CD3E | 5.6014369 | 6.1305860 | 5.6065755 | 6.6411912 | 6.5623125 | 6.196839143 | 7.9801210 | 6.8196870 | 5.73016986 | 0.2693334 | 5.17075686 | 6.8601808 | 0.8736744 |
|  |  | CD3D | 8.3740612 | 9.5900426 | 6.9907741 | 8.8065324 | 10.904517 | 11.59045534 | 14.704356 | 13.043872 | 15.8556641 | 0.1896499 | 6.147821161 | 9.6358016 | 0.3104679 |
|  |  | CD3G | 2.9616236 | 2.4975328 | 2.5129583 | 3.1574288 | 3.1809931 | 3.691928276 | 3.5768544 | 3.1697304 | 3.382031536 | 0.0563528 | 2.472850208 | 3.0951497 | 0.1841549 |
|  | CD4+ | CD4 | 0.6745009 | 0.7288364 | 0.380203 | 0.7744559 | 1.11699392 | 0.998706654 | 1.5584297 | 1.4234413 | 1.06157231 | 2.1410940 | 0.455593832 | 0.451870 | 0 |
|  | CD8A+ | CD8A | 0.1256019 | 0.5132227 | 0.7593965 | 0.5488960 | 0.0970343 | 0.229441889 | 0.2025171 | 0.1895874 | 0.103571525 | 0.0332500 | 0.586658358 | 0.3755483 | 0 |
| naїve T cell | CCR7+ | CCR7 | 2.7713978 | 1.5168921 | 2.4678707 | 1.3936907 | 0.2612329 | 1.390502098 | 0.9966204 | 0.4889463 | 0.923473357 | 0.7930387 | 1.023871886 | 1.1913744 | 0.4143137 |
|  | SELL+ | SELL | 2.7172481 | 1.7827060 | 1.8807026 | 1.02489753 | 0.1342057 | 1.135877721 | 0.740148 | 2.6301018 | 1.393320964 | 4.1045651 | 0.584338157 | 0.8741776 | 1.5413107 |
|  | CD5 | CD5 | 0.8737197 | 0.7558089 | 0.8633605 | 0.6765405 | 1.3823332 | 1.293823194 | 1.2711661 | 1.0170637 | 0.985723518 | 0.0479992 | 0.590355283 | 0.919617 | 0.0519008 |
|  |  | IL7R | 8.77459776 | 10.748687 | 6.7029821 | 12.1009727 | 3.9762266 | 6.029532185 | 3.7804820 | 3.1160104 | 4.628238514 | 0.2137897 | 7.793276063 | 5.708879 | 13.987940 |
|  |  | LEF1 | 1.0301812 | 0.8118300 | 0.8309910 | 0.59170766 | 0.2615737 | 0.68302869 | 0.5433539 | 0.5291838 | 0.402135258 | 0 | 0.531820292 | 0.3889932 | 0 |
|  |  | TCF7 | 1.3500024 | 1.6282955 | 1.235916 | 0.7933115 | 0.5491275 | 0.987110353 | 1.0882281 | 0.5376217 | 1.000520582 | 0.2319182 | 1.02664888 | 0.4714386 | 2.2720429 |
| memory |  | EOMES | 0.0297674 | 0.02118204 | 0.0323794 | 0.0234567 | 0.0233397 | 0.088407908 | 0.0650442 | 0.0131591 | 0.050089975 | 0.0064520 | 0.020584459 | 0.1393782 | 0 |
|  |  | GZMK | 1.56666996 | 3.7859816 | 2.217063 | 1.5309890 | 0.6031974 | 1.825110239 | 0.4163382 | 0.4514978 | 0.84553539 | 0.0719562 | 1.747139351 | 3.9154122 | 0 |
|  |  | CXCR3 | 0.4435628 | 0.4902326 | 0.642003 | 0.4234359 | 1.6838888 | 0.577584157 | 1.2356912 | 1.1917185 | 1.090532754 | 2.2929321 | 0.365668317 | 0.7108942 | 0.19171361 |
| memory T cell | CD44 | CD44 | 5.6967935 | 4.8604045 | 6.0047350 | 5.6269919 | 8.9088142 | 4.304681492 | 4.2937452 | 5.7232046 | 4.502197271 | 1.3261640 | 6.093347762 | 5.4041090 | 7.1960798 |
|  | IFNG | IFNG | 0.3662446 | 1.6419712 | 0.5052018 | 0.4591648 | 3.5804523 | 2.02539403 | 2.5726823 | 0.2446624 | 0.576278463 | 0.0513032 | 1.367756487 | 1.1311043 | 0 |
|  | S100A4 | S100A4 | 18.187261 | 15.656819 | 15.831824 | 19.581297 | 55.0542225 | 21.59468951 | 23.060957 | 38.9787039 | 30.90130588 | 4.4438859 | 25.58119284 | 27.403250 | 14.459892 |
| effector | CD44 | CD44 | 5.6967935 | 4.8604045 | 6.0047350 | 5.6269919 | 8.9088142 | 4.304681492 | 4.2937452 | 5.7232046 | 4.502197271 | 1.3261640 | 6.093347762 | 5.4041090 | 7.1960798 |
|  | FAS | FAS | 0.4359174 | 0.48459758 | 0.5505730 | 0.6184435 | 0.7290670 | 0.326726731 | 0.4211240 | 1.0361104 | 0.784765629 | 0.0485326 | 0.407443082 | 0.3225754 | 0.0549710 |
|  | IFNG | IFNG | 0.3662446 | 1.6419712 | 0.5052018 | 0.4591648 | 3.5804523 | 2.02539403 | 2.5726823 | 0.2446624 | 0.576278463 | 0.0513032 | 1.367756487 | 1.1311043 | 0 |
|  | CD44hi | CD44 | 5.6967935 | 4.8604045 | 6.0047350 | 5.6269919 | 8.9088142 | 4.304681492 | 4.2937452 | 5.7232046 | 4.502197271 | 1.3261640 | 6.093347762 | 5.4041090 | 7.1960798 |
| effector memory |  | FGFBP2 | 0.0678557 | 0.0059864 | 0.1204008 | 0.0217277 | 0.0565465 | 0.034368168 | 0.0149718 | 0.0668333 | 0.007246003 | 0.0600640 | 0.034268066 | 0 | 0.1433998 |
|  |  | KLRD1 | 0.0694597 | 0.1161074 | 0.2030099 | 0.0966641 | 0.0513290 | 0.046121459 | 0.0473441 | 0.09558035 | 0.015122595 | 0.0637575 | 0.252755628 | 0.0329293 | 0.4168851 |
|  | effector memory | S100A4 | 18.187261 | 15.656819 | 15.831824 | 19.581297 | 55.0542225 | 21.59468951 | 23.060957 | 38.9787039 | 30.90130588 | 4.4438859 | 25.58119284 | 27.403250 | 14.459892 |
|  |  | ANXA1 | 10.806259 | 9.2493421 | 13.83156 | 10.269136 | 21.379070 | 10.84286138 | 3.3384253 | 1.0246031 | 2.665062594 | 0.7769560 | 19.37708665 | 8.5547972 | 13.261841 |
|  |  | CD40LG | 1.0970187 | 0.7191653 | 0.5184434 | 1.8709727 | 1.3700288 | 1.458503399 | 1.5512070 | 0.1395498 | 0.487878785 | 0.0210205 | 0.677287729 | 1.3264463 | 0.4812160 |
|  |  | CXCR6 | 0.2687522 | 0.5312893 | 0.6205779 | 2.1343801 | 2.6621233 | 1.20835543 | 2.0927597 | 1.8944116 | 1.783810392 | 0.0076659 | 0.252901381 | 0.5462483 | 0.3764993 |
|  |  | CXCR3 | 0.4435628 | 0.4902326 | 0.642003 | 0.4234359 | 1.6838888 | 0.577584157 | 1.2356912 | 1.1917185 | 1.090532754 | 2.2929321 | 0.365668317 | 0.7108942 | 0.19171361 |
| treg |  | IL2RA | 0.2638472 | 0.2572764 | 0.2178870 | 0.1696723 | 0.2670200 | 0.098317272 | 0.4369348 | 5.9796598 | 1.396967739 | 0.0185824 | 0.181231427 | 0.3026547 | 0.4057534 |
|  |  | FOXP3 | 0.0705484 | 0.0875113 | 0.0710763 | 0.0628714 | 0.02625733 | 0.051055299 | 0.0581074 | 2.2886596 | 1.260066727 | 0.0115688 | 0.021281356 | 0.0925351 | 0 |
|  |  | IKZF2 | 0.0709386 | 0.0428953 | 0.0276915 | 0.0552687 | 0.0125976 | 0.06294893 | 0.0601528 | 0.6854224 | 0.325223121 | 0.0506574 | 0.006722924 | 0.0283380 | 0.15031265 |
|  |  | CTLA4 | 0.3931480 | 0.2068663 | 0.3413997 | 0.5449937 | 0.6684993 | 0.444880124 | 1.4528413 | 3.04618947 | 0.80011604 | 0.0061254 | 0.41086908 | 0.8276045 | 0 |
| th17 |  | CTSH | 0.2835783 | 0.4939914 | 0.1657683 | 0.5967847 | 0.6213228 | 1.142345865 | 1.3827075 | 0.1723218 | 0.168510514 | 0.65959521 | 0.422874532 | 1.0661575 | 0.90278595 |
| tfh |  | CD200 | 0.0656933 | 0.1005229 | 0.0412372 | 0 | 0.0214302 | 0.155731156 | 0.8575308 | 0.1041409 | 0.138876436 | 0.0021749 | 0.006164635 | 0 | 0.0632403 |
|  |  | PTPN13 | 0.0626711 | 0.0789027 | 0.0105230 | 0.1349375 | 0.0432535 | 0.06772205 | 0.265101 | 0.0188724 | 0.060579358 | 0.0072161 | 0.03904836 | 0.0536802 | 0 |
|  |  | BTLA | 0.12989537 | 0.2528388 | 0.0598026 | 0.0652081 | 0.0692065 | 0.17397593 | 0.6658419 | 0.2571503 | 0.241766754 | 0.1066602 | 0.046323645 | 0.1382864 | 0 |
| activated |  | TNF | 0.2903434 | 0.2699594 | 0.2032615 | 0.3621180 | 0.8110219 | 0.702920225 | 0.3335265 | 0.37068277 | 0.40167419 | 0.1084277 | 0.607211732 | 0.6611090 | 0.4313545 |
|  |  | IFNG | 0.3662446 | 1.6419712 | 0.5052018 | 0.4591648 | 3.5804523 | 2.02539403 | 2.5726823 | 0.2446624 | 0.576278463 | 0.0513032 | 1.367756487 | 1.1311043 | 0 |
|  |  | FOS | 18.441738 | 15.872270 | 11.41145 | 25.623185 | 10.008231 | 5.377841993 | 9.8241051 | 7.4085122 | 10.99658536 | 13.756446 | 8.165944511 | 19.888514 | 36.209794 |
|  |  | JUN | 14.432210 | 8.4817923 | 13.637137 | 25.176299 | 17.546542 | 19.34216966 | 16.082480 | 13.975847 | 24.36908474 | 3.3410371 | 8.876479831 | 27.263890 | 11.000868 |
| exhausted/activated |  | LAG3 | 0.2111946 | 0.51266301 | 0.2998111 | 0.5613682 | 0.4825310 | 0.323613447 | 1.5207244 | 1.8178374 | 0.377664421 | 0.0900033 | 0.454920597 | 0.2893416 | 0 |
|  |  | HAVC | 0.0553805 | 0.13313511 | 0.0537093 | 0.2108132 | 0.1252293 | 0.223725848 | 0.6093606 | 0.6140274 | 0.302098376 | 0.1174203 | 0.060557102 | 0.0733008 | 0.11821585 |
|  |  | PDCD1 | 0.2247869 | 0.2208344 | 0.4020229 | 0.7554177 | 0.4268227 | 0.631703088 | 1.590793 | 0.4620380 | 0.697547629 | 0.0461469 | 0.456800714 | 0.2169356 | 0.0202850 |
| exhausted |  | GZMB | 0.3399286 | 1.22587886 | 0.6364054 | 0.3619023 | 4.5426735 | 0.382991867 | 1.7699288 | 0.86385892 | 0.622291729 | 50.982269 | 0.812614966 | 0.0297666 | 0.28933157 |
|  |  | ENTPD1 | 0.0621103 | 0.0468075 | 0.0839683 | 0.0053073 | 0.29673942 | 0.046030826 | 0.3527491 | 1.3821487 | 1.027360043 | 0.1646973 | 0.007373892 | 0.0192775 | 0.1580921 |
|  |  | ITGAE | 0.30227076 | 0.5453431 | 0.3576059 | 0.7683669 | 0.8922849 | 0.788438779 | 1.0185448 | 0.60166013 | 0.592559117 | 0.8380842 | 0.446293934 | 0.325291 | 0.4327020 |
| γδT |  | TRDC | 0.11084241 | 0.0852994 | 0.0680136 | 0.04431155 | 0.1066206 | 0.344208711 | 0.0270127 | 0.1701089 | 0.222144498 | 0.02237508 | 0.036033787 | 0 | 5.1641208 |
|  |  | TRGC1 | 0.1234095 | 0.1012921 | 0.1141080 | 0.1207053 | 0.12383608 | 0.148796579 | 0.080243 | 0.0367047 | 0.10009449 | 0 | 0.157738298 | 0.1966087 | 1.1166746 |
|  |  | TRGC2 | 0.2686184 | 0.67001342 | 0.1694247 | 0.3072064 | 0.2618506 | 0.427888085 | 0.3034818 | 0.0678530 | 0.18821963 | 0.02331947 | 0.477065086 | 0.4472447 | 0.5352021 |
|  | cytotoxic | GNLY | 0.6802616 | 1.390066 | 0.7498857 | 0.4896724 | 0.7352220 | 0.984615485 | 0.5406618 | 1.6716046 | 0.384622917 | 0.3538522 | 0.885892243 | 0.1641045 | 1.6536798 |
|  |  | IFNG | 0.3662446 | 1.6419712 | 0.505201 | 0.4591648 | 3.5804523 | 2.02539403 | 2.5726823 | 0.2446624 | 0.576278463 | 0.0513032 | 1.367756487 | 1.1311043 | 0 |
|  |  | NKG7 | 0.6442154 | 1.4818170 | 1.5695229 | 0.5538727 | 1.0298200 | 1.056669377 | 0.658129 | 0.6199345 | 0.450304563 | 1.0825206 | 2.001220642 | 1.1403985 | 0.0763007 |
|  |  | PRF1 | 0.29365377 | 0.2153472 | 0.4376148 | 0.7799206 | 0.7015961 | 0.305228265 | 0.6909179 | 0.9390548 | 0.77735105 | 0.0400210 | 0.353914708 | 0.4436147 | 0.2397588 |
|  |  | GZMA | 1.4554193 | 4.0936010 | 2.5476497 | 3.4124547 | 7.1057577 | 4.919905321 | 2.615820 | 0.7603251 | 2.338680201 | 0.1131281 | 2.726255729 | 4.2958096 | 0.1755888 |
|  |  | GZMB | 0.3399286 | 1.2258788 | 0.6364054 | 0.3619023 | 4.542673 | 0.382991867 | 1.7699288 | 0.86385892 | 0.622291729 | 50.982269 | 0.812614966 | 0.0297666 | 0.28933157 |
|  |  | GZMK | 1.56666996 | 3.7859816 | 2.217063 | 1.5309890 | 0.6031974 | 1.825110239 | 0.4163382 | 0.4514978 | 0.84553539 | 0.0719562 | 1.747139351 | 3.9154122 | 0 |
|  | inhibitory | HAVCR2 | 0.0553805 | 0.13313511 | 0.0537093 | 0.2108132 | 0.1252293 | 0.223725848 | 0.6093606 | 0.6140274 | 0.302098376 | 0.1174203 | 0.060557102 | 0.0733008 | 0.11821585 |
|  |  | PDCD1 | 0.2247869 | 0.2208344 | 0.4020229 | 0.7554177 | 0.4268227 | 0.631703088 | 1.590793 | 0.4620380 | 0.697547629 | 0.0461469 | 0.456800714 | 0.2169356 | 0.0202850 |
|  |  | LAG3 | 0.2111946 | 0.51266301 | 0.2998111 | 0.5613682 | 0.4825310 | 0.323613447 | 1.5207244 | 1.8178374 | 0.377664421 | 0.0900033 | 0.454920597 | 0.2893416 | 0 |
|  |  | TIGIT | 0.72754719 | 0.7785882 | 0.5975552 | 0.5280065 | 0.2268517 | 0.590570363 | 3.867458 | 7.3808562 | 4.529947723 | 0.0655676 | 0.255488036 | 0.5790920 | 0.0831188 |
|  |  | CTLA4 | 0.3931480 | 0.2068663 | 0.3413997 | 0.5449937 | 0.6684993 | 0.444880124 | 1.4528413 | 3.04618947 | 0.80011604 | 0.0061254 | 0.41086908 | 0.8276045 | 0 |
|  |  | BTLA | 0.12989537 | 0.2528388 | 0.0598026 | 0.0652081 | 0.0692065 | 0.17397593 | 0.6658419 | 0.2571503 | 0.241766754 | 0.1066602 | 0.046323645 | 0.1382864 | 0 |
|  | Transcript factors | ZNF683 | 0.0252310 | 0.0661061 | 0.0203662 | 0.0103855 | 0.1716883 | 0.045948714 | 0.0168988 | 0.04827107 | 0.016841489 | 0.0080760 | 0.237802065 | 0.0889500 | 0 |
|  |  | HOPX | 0.5284029 | 1.0159156 | 0.5340212 | 0.73974995 | 2.7360941 | 0.993218381 | 0.5761886 | 0.1252767 | 0.109743092 | 0.0395441 | 1.425533145 | 1.4346087 | 1.5094773 |
|  |  | TOX | 0.1585891 | 0.2312898 | 0.1461340 | 0.2314077 | 0.19531196 | 0.287146622 | 0.9245396 | 0.4553533 | 0.417261845 | 0.0073963 | 0.252661345 | 0.3754779 | 0.5053016 |
|  |  | HIF1A | 0.6943405 | 0.75532348 | 0.7630414 | 0.78758355 | 0.7589947 | 0.619710293 | 1.1722535 | 0.7276239 | 0.81127651 | 0.3854983 | 0.646630281 | 0.4386910 | 1.0203951 |
|  |  | TBX21 | 0.1248769 | 0.1393586 | 0.117564 | 0.2718458 | 0.2310524 | 0.213072302 | 0.1728401 | 0.1935912 | 0.143064776 | 0.0058988 | 0.219349651 | 0.3000081 | 0 |
|  |  | EOMES | 0.0297674 | 0.02118204 | 0.0323794 | 0.0234567 | 0.0233397 | 0.088407908 | 0.0650442 | 0.0131591 | 0.050089975 | 0.0064520 | 0.020584459 | 0.1393782 | 0 |
|  | Proliferation | MKI67 | 0.00684857 | 0.0416038 | 0 | 0.0075936 | 0.0353260 | 0.046256122 | 0.0435881 | 0.0195444 | 0 | 0 | 0 | 0 | 0 |
|  |  | CDK1 | 0.0234904 | 0.0683252 | 0.0221235 | 0.0332551 | 0.1317472 | 0.10028714 | 0.0787870 | 0.0305698 | 0.049806162 | 0.06916656 | 0.085284965 | 0.0250432 | 0 |
|  |  | STMN1 | 0.3033075 | 0.5078279 | 0.3014483 | 0.3251541 | 0.3452639 | 0.447168867 | 0.4039367 | 0.5797253 | 0.341773278 | 4.9096379 | 1.84197557 | 0.2681753 | 0.2609393 |

**Table S5. The average expression matrix of marker genes in CD8+ T cell subtypes**

|  |  | **GeneName** | **Cluster 0** | **Cluster 6** | **Cluster 1** | **Cluster 9** | **Cluster 13** | **Cluster 2** | **Cluster 3** | **Cluster 4** | **Cluster 5** | **Cluster 10** | **Cluster 7** | **Cluster 8** | **Cluster 11** | **Cluster 12** | **Cluster 14** |
| --- | --- | --- | --- | --- | --- | --- | --- | --- | --- | --- | --- | --- | --- | --- | --- | --- | --- |
|  |  |  | **Effector memory** | **Effector memory** | **naïve T cell** | **naïve T cell** | **naïve T cell** | **GZMK+Effector** | **Proliferation** | **cytotoxic** | **cytotoxic** | **cytotoxic** | **KLRC1+Effector** | **exhausted** | **γδT** | **memory** | **Effector** |
|  |  | PTPRC | 8.577855848 | 12.10630734 | 9.372825647 | 5.006916762 | 7.435598683 | 10.41434569 | 8.81515882 | 7.188203072 | 5.805792933 | 8.667290599 | 8.579269425 | 8.859294254 | 8.997099306 | 6.765129343 | 9.815845353 |
|  | CD3E+ | CD3E | 6.614662291 | 5.750406803 | 7.614765265 | 5.920717074 | 6.340562653 | 8.240789161 | 6.72713346 | 6.262708065 | 7.063251829 | 7.09427507 | 9.077381141 | 9.184401321 | 7.609841439 | 6.822895091 | 7.195394248 |
|  |  | CD3D | 12.70499938 | 10.20852545 | 13.02425675 | 7.121792758 | 11.30704002 | 13.24972241 | 9.755940049 | 14.7347112 | 13.63571582 | 13.34092601 | 14.68112343 | 18.7475908 | 9.745307089 | 10.32938797 | 13.48233968 |
|  |  | CD3G | 3.596812806 | 3.611895074 | 4.063764241 | 2.513491669 | 4.142687446 | 4.20564451 | 3.374566886 | 3.491616329 | 3.223585991 | 3.481902534 | 5.165805372 | 5.154260975 | 3.414148904 | 3.029837526 | 3.973036655 |
|  | CD4+ | CD4 | 0.245540138 | 0.056837009 | 0.115416533 | 0.014214976 | 0.172871166 | 0.222541996 | 0.095860587 | 0.171655273 | 0.108505576 | 0.018797876 | 0.049566968 | 0.223675752 | 0 | 0.054894555 | 0.431848085 |
|  | CD8A+ | CD8A | 5.067740204 | 6.355104906 | 5.70688341 | 3.59001291 | 4.34564133 | 5.461289668 | 4.300921605 | 4.319154659 | 3.704394831 | 6.458363093 | 7.09943312 | 5.842878276 | 1.802678984 | 5.335029632 | 5.208133515 |
| naïve T cell | CCR7+ | CCR7 | 0.281215317 | 0.091003952 | 0.610882849 | 4.82958786 | 0.411316308 | 0.166504526 | 0.142761987 | 0.502160518 | 0.658734116 | 0.017310322 | 0.198156048 | 0.062525643 | 0 | 0.196283041 | 0.113812255 |
|  | SELL+ | SELL | 0.188112475 | 0.099100281 | 0.604951463 | 5.297348362 | 1.127473953 | 0.107775642 | 0.341573191 | 0.201236984 | 0.595877646 | 0.014492124 | 0.13562641 | 0.113093575 | 0.07283118 | 0.114228806 | 0.290262919 |
|  | CD5 | CD5 | 0.546679975 | 0.47518976 | 0.711764991 | 0.603737712 | 0.394064137 | 0.847041176 | 0.74734851 | 0.609156491 | 0.545921461 | 0.423073793 | 0.329464019 | 0.35235886 | 0.226231592 | 0.482890783 | 0.465349666 |
|  |  | IL7R | 2.086271391 | 2.567115907 | 3.511178688 | 7.241725848 | 2.790414595 | 0.696271327 | 2.160123588 | 1.064753603 | 2.3581012 | 0.174331873 | 7.253857809 | 0.782705972 | 1.325449491 | 9.694022696 | 1.576183826 |
|  |  | LEF1 | 0.108901299 | 0.041589248 | 0.191241816 | 1.683617147 | 0.174787254 | 0.038563071 | 0.100081758 | 0.045696469 | 0.105534215 | 0.012806639 | 0.096502217 | 0.221338718 | 0.277222135 | 0.02554931 | 0 |
|  |  | TCF7 | 0.685146 | 0.698182411 | 0.82817937 | 1.71365272 | 0.584530638 | 0.444736524 | 0.743068805 | 0.889651233 | 1.124404482 | 0.331517183 | 0.433531962 | 0.511209331 | 1.003137052 | 1.066645534 | 0.180744267 |
| memory T cell | CD44 | CD44 | 9.154284933 | 13.56890781 | 6.50684313 | 5.024655706 | 6.58012214 | 7.68030409 | 5.329599898 | 6.144635417 | 5.482479811 | 3.300028616 | 6.04552421 | 5.305595507 | 8.663138482 | 6.228623233 | 3.704766211 |
|  | IFNG | IFNG | 7.759260775 | 4.006695455 | 3.208444821 | 0.029893595 | 6.645226937 | 7.305947838 | 7.884933545 | 14.01782563 | 8.188334884 | 8.742687689 | 2.055487997 | 6.874787174 | 5.107893675 | 1.472560133 | 4.179281068 |
|  | S100A4+ | S100A4 | 47.34794049 | 20.50193648 | 14.78394245 | 8.221156975 | 16.47341885 | 7.50247498 | 18.05810561 | 11.8873829 | 10.32667736 | 18.7676757 | 19.2563937 | 23.59261523 | 33.363635 | 26.15584204 | 26.51461494 |
| effector | CD44 | CD44 | 9.154284933 | 13.56890781 | 6.50684313 | 5.024655706 | 6.58012214 | 7.68030409 | 5.329599898 | 6.144635417 | 5.482479811 | 3.300028616 | 6.04552421 | 5.305595507 | 8.663138482 | 6.228623233 | 3.704766211 |
|  | FAS | FAS | 0.540668146 | 0.5139162 | 0.388523347 | 0.169571099 | 0.681883262 | 0.427523167 | 0.339520232 | 0.502801961 | 0.578125367 | 0.348573124 | 0.380666706 | 0.220170924 | 0.149383753 | 0.386609189 | 0.230153641 |
|  | IFNG | IFNG | 7.759260775 | 4.006695455 | 3.208444821 | 0.029893595 | 6.645226937 | 7.305947838 | 7.884933545 | 14.01782563 | 8.188334884 | 8.742687689 | 2.055487997 | 6.874787174 | 5.107893675 | 1.472560133 | 4.179281068 |
|  | CD44hi | CD44 | 9.154284933 | 13.56890781 | 6.50684313 | 5.024655706 | 6.58012214 | 7.68030409 | 5.329599898 | 6.144635417 | 5.482479811 | 3.300028616 | 6.04552421 | 5.305595507 | 8.663138482 | 6.228623233 | 3.704766211 |
| effector memory |  | FGFBP2 | 0.137360751 | 0.065416515 | 0.664157332 | 0.111742698 | 0.165976904 | 0.111311957 | 0.617801478 | 0.121319279 | 0.158880048 | 0.344992832 | 0.052100229 | 0.193424672 | 0.145150589 | 0.037936267 | 0 |
|  |  | KLRD1 | 2.211384251 | 2.962105336 | 1.605161794 | 0.260485895 | 1.097161347 | 1.427097854 | 2.309153287 | 0.791306698 | 2.92408245 | 2.278796607 | 5.355576226 | 4.316050315 | 5.405687798 | 0.864401595 | 2.47269855 |
|  | effector memory | S100A4 | 47.34794049 | 20.50193648 | 14.78394245 | 8.221156975 | 16.47341885 | 7.50247498 | 18.05810561 | 11.8873829 | 10.32667736 | 18.7676757 | 19.2563937 | 23.59261523 | 33.363635 | 26.15584204 | 26.51461494 |
|  |  | ANXA1 | 19.45265882 | 43.07206005 | 10.55924633 | 11.13237239 | 12.51975056 | 6.043647015 | 13.49447292 | 9.703719374 | 8.417938368 | 5.46176532 | 10.79324521 | 6.025464972 | 18.33084469 | 9.345423605 | 9.264570631 |
|  |  | CD40LG | 0.192032498 | 0.040430742 | 0.089662201 | 0.088657483 | 0.562200143 | 0.099677759 | 0.103753197 | 0.066996877 | 0.140842121 | 0.020176774 | 0.075951499 | 0.131681929 | 0 | 0.705877568 | 0.19222972 |
|  |  | CXCR6 | 1.677713038 | 1.190077673 | 0.756901354 | 0.00897737 | 0.936173238 | 2.208748579 | 0.791356113 | 0.742143563 | 0.963403349 | 0.775996067 | 1.870617127 | 2.54015587 | 0.523613728 | 2.448807846 | 1.48862536 |
|  |  | CXCR3 | 1.669572216 | 1.411723937 | 1.051813782 | 0.367053819 | 0.908459398 | 0.928235009 | 0.602753303 | 1.186656232 | 0.771860252 | 0.940557757 | 0.95670331 | 0.860028386 | 0.841702064 | 0.212333315 | 1.764140678 |
| treg |  | IL2RA | 0.057045657 | 0.10737281 | 0.051646517 | 0.023846628 | 0.065731526 | 0.015585493 | 0.04900765 | 0.014603179 | 0.158369034 | 0 | 0.021308691 | 0.046429039 | 0.045872497 | 0.033568312 | 0 |
|  |  | FOXP3 | 0.008768805 | 0.006901902 | 0.022069201 | 0 | 0 | 0.012763638 | 0.009692205 | 0.02009506 | 0.035498515 | 0 | 0.006143955 | 0.026827501 | 0 | 0 | 0 |
|  |  | IKZF2 | 0.037888773 | 0.098753797 | 0.070697458 | 0.045401785 | 0.038963569 | 0.028247561 | 0.088056399 | 0.0943336 | 0.213416717 | 0 | 0.013832942 | 0.02128511 | 0.285323845 | 0.538142307 | 0 |
|  |  | CTLA4 | 0.31794797 | 0.278986204 | 0.226405062 | 0.071392954 | 0.614969774 | 0.254800268 | 0.144298314 | 0.039738927 | 0.183851903 | 0.105747222 | 0.215598141 | 0.735404165 | 0.092269162 | 0.120905381 | 0.387074105 |
|  |  |  | #N/A | #N/A | #N/A | #N/A | #N/A | #N/A | #N/A | #N/A | #N/A | #N/A | #N/A | #N/A | #N/A | #N/A | #N/A |
| th17 |  | CTSH | 0.344990938 | 0.118254546 | 0.106802717 | 0.081190926 | 0.247220216 | 0.141040367 | 0.306628118 | 0.121122001 | 0.245087336 | 0.054823014 | 0.338327757 | 0.569384499 | 0.131505133 | 0.880698888 | 0.471987906 |
| tfh |  | CD200 | 0 | 0 | 0.012149838 | 0 | 0 | 0.004526984 | 0.002316165 | 0.052664222 | 0.03448593 | 0 | 0 | 0.128260006 | 0.019231879 | 0 | 0 |
|  |  | PTPN13 | 0.000739508 | 0 | 0.003942291 | 0 | 0 | 0.002176138 | 0.009454226 | 0 | 0.006729702 | 0 | 0 | 0.006484958 | 0 | 0.022217285 | 0 |
|  |  | BTLA | 0.045996125 | 0.012069536 | 0.02893171 | 0.038491487 | 0.059990881 | 0.009653764 | 0.025940806 | 0.030432804 | 0.063884063 | 0.052267992 | 0.086413015 | 0.153479855 | 0 | 0.064884533 | 0 |
| activated |  | TNF | 1.041974773 | 0.512124896 | 0.412493219 | 0.14009726 | 0.59010861 | 0.703686244 | 0.781944244 | 0.911343236 | 0.682587815 | 0.516087809 | 0.580508798 | 0.491347537 | 0.583370523 | 0.601854223 | 0.267673024 |
|  |  | IFNG | 7.759260775 | 4.006695455 | 3.208444821 | 0.029893595 | 6.645226937 | 7.305947838 | 7.884933545 | 14.01782563 | 8.188334884 | 8.742687689 | 2.055487997 | 6.874787174 | 5.107893675 | 1.472560133 | 4.179281068 |
|  |  | FOS | 12.75692414 | 11.08256441 | 13.74509853 | 16.86664699 | 16.87044956 | 11.5367793 | 4.232100904 | 8.615391671 | 10.74412935 | 10.58121083 | 16.77452022 | 11.44503569 | 19.82801644 | 25.36729447 | 14.37445581 |
|  |  | JUN | 19.2036961 | 22.05837098 | 19.08329131 | 11.39949626 | 18.90956245 | 16.07016946 | 11.90177091 | 27.22135801 | 9.952531661 | 15.91019182 | 22.61220947 | 25.51874023 | 20.24213254 | 27.46914462 | 28.01347463 |
| exhausted/activated |  | LAG3 | 3.315597264 | 2.948558104 | 2.223359058 | 0.647544395 | 5.184584526 | 1.505594513 | 2.075046396 | 2.32907838 | 2.555856693 | 5.278212503 | 1.723627244 | 2.494073524 | 2.66211061 | 2.197307214 | 1.856409446 |
|  |  | HAVCR2 | 0.191296208 | 0.249795294 | 0.318992215 | 0.18990863 | 0 | 0.479634015 | 0.109594626 | 0.348165802 | 1.011572996 | 0 | 0.424052697 | 1.647751553 | 0.479106817 | 0.251283151 | 0 |
|  |  | PDCD1 | 0.541520788 | 1.361267814 | 0.62711062 | 0.118222187 | 0.413038879 | 0.869941307 | 0.541342373 | 0.560122618 | 0.49020913 | 0.041695668 | 0.656556474 | 1.254261286 | 0.257182028 | 0.274685489 | 0.589410386 |
| exhausted |  | GZMB | 23.42849028 | 19.43883643 | 6.40021583 | 0.441349445 | 7.797325724 | 4.56133153 | 5.275744238 | 6.152572832 | 12.63608582 | 47.13470281 | 13.26265256 | 9.988347933 | 14.12598409 | 1.416326311 | 3.206561065 |
|  |  | ENTPD1 | 0.23669823 | 0.124881592 | 0.064546199 | 0.132967985 | 0.170397168 | 0.018198785 | 0.086556384 | 0.324790631 | 0.444661853 | 0 | 0.134133303 | 1.904418176 | 0.070819396 | 0.042808219 | 0 |
|  |  | ITGAE | 1.64057587 | 1.340409807 | 0.801520351 | 0.31966695 | 0.850497031 | 0.98566674 | 0.652136229 | 1.042713979 | 1.082471891 | 1.053365646 | 2.043719314 | 2.47894381 | 1.020495788 | 1.043153402 | 1.21300744 |
|  |  |  | #N/A | #N/A | #N/A | #N/A | #N/A | #N/A | #N/A | #N/A | #N/A | #N/A | #N/A | #N/A | #N/A | #N/A | #N/A |
| γδT |  | TRDC | 0.521638116 | 0.373842904 | 0.677451796 | 0.144100864 | 0.349866841 | 0.245201253 | 0.873833844 | 0.735759945 | 2.555697388 | 0.732884898 | 0.297878884 | 0.12472787 | 15.44716664 | 0.793252287 | 0.706219542 |
|  |  | TRGC1 | 0.393125606 | 0.274745167 | 0.664517539 | 0.21038713 | 0.672459103 | 0.331911182 | 0.552677383 | 0.542587746 | 0.614149965 | 0.278320333 | 0.328186583 | 0.453958161 | 1.71383471 | 1.904999529 | 0.889366317 |
|  |  | TRGC2 | 2.095676916 | 1.854337774 | 2.855435936 | 0.510211111 | 2.146814882 | 2.057502702 | 3.840343873 | 2.145908582 | 4.305320939 | 8.744685434 | 2.690085206 | 2.557465475 | 8.582552284 | 3.416526209 | 3.732603489 |
|  |  |  | #N/A | #N/A | #N/A | #N/A | #N/A | #N/A | #N/A | #N/A | #N/A | #N/A | #N/A | #N/A | #N/A | #N/A | #N/A |
|  | cytotoxic | GNLY | 12.03242693 | 6.087959672 | 7.03290297 | 1.462574436 | 5.351991253 | 1.223399076 | 5.365886402 | 9.104422824 | 18.3360851 | 0.46628456 | 8.464069716 | 18.6714151 | 22.01142003 | 1.468566674 | 9.062420185 |
|  |  | IFNG | 7.759260775 | 4.006695455 | 3.208444821 | 0.029893595 | 6.645226937 | 7.305947838 | 7.884933545 | 14.01782563 | 8.188334884 | 8.742687689 | 2.055487997 | 6.874787174 | 5.107893675 | 1.472560133 | 4.179281068 |
|  |  | NKG7 | 17.32385877 | 12.82891896 | 19.62781388 | 2.161474148 | 12.45558667 | 16.17611986 | 17.55888486 | 17.93270923 | 20.28597785 | 40.037622 | 8.069116011 | 10.34919851 | 9.857222474 | 9.01250768 | 17.45170366 |
|  |  | PRF1 | 3.263312124 | 5.755314748 | 2.828288938 | 0.198428732 | 2.910410426 | 2.003879068 | 2.346364073 | 2.063094147 | 2.21421365 | 6.76606573 | 3.566201671 | 3.75210496 | 3.554148761 | 4.123839295 | 2.37171066 |
|  |  | GZMA | 16.66721176 | 10.7852408 | 14.26897776 | 0.907081405 | 14.45233225 | 20.45998637 | 12.07298186 | 19.63415503 | 24.02090956 | 18.35228659 | 12.09337215 | 19.21715499 | 1.950870798 | 9.680779939 | 20.05076766 |
|  |  | GZMB | 23.42849028 | 19.43883643 | 6.40021583 | 0.441349445 | 7.797325724 | 4.56133153 | 5.275744238 | 6.152572832 | 12.63608582 | 47.13470281 | 13.26265256 | 9.988347933 | 14.12598409 | 1.416326311 | 3.206561065 |
|  |  | GZMK | 6.10063428 | 7.223270611 | 15.09266737 | 3.624999512 | 13.88999475 | 24.29560084 | 8.993803982 | 19.3522786 | 14.9647428 | 16.33193419 | 2.589232478 | 0.53911109 | 0.593999824 | 4.826706261 | 7.589224654 |
|  | inhibitory | HAVCR2 | 0.191296208 | 0.249795294 | 0.318992215 | 0.18990863 | 0 | 0.479634015 | 0.109594626 | 0.348165802 | 1.011572996 | 0 | 0.424052697 | 1.647751553 | 0.479106817 | 0.251283151 | 0 |
|  |  | PDCD1 | 0.541520788 | 1.361267814 | 0.62711062 | 0.118222187 | 0.413038879 | 0.869941307 | 0.541342373 | 0.560122618 | 0.49020913 | 0.041695668 | 0.656556474 | 1.254261286 | 0.257182028 | 0.274685489 | 0.589410386 |
|  |  | LAG3 | 3.315597264 | 2.948558104 | 2.223359058 | 0.647544395 | 5.184584526 | 1.505594513 | 2.075046396 | 2.32907838 | 2.555856693 | 5.278212503 | 1.723627244 | 2.494073524 | 2.66211061 | 2.197307214 | 1.856409446 |
|  |  | TIGIT | 0.966303501 | 0.969602843 | 1.661256893 | 0.532888755 | 1.168130238 | 1.318771673 | 1.018005618 | 1.619078878 | 2.323886509 | 2.010307726 | 0.980155105 | 2.973184558 | 1.129352557 | 0.152415672 | 1.111731648 |
|  |  | CTLA4 | 0.31794797 | 0.278986204 | 0.226405062 | 0.071392954 | 0.614969774 | 0.254800268 | 0.144298314 | 0.039738927 | 0.183851903 | 0.105747222 | 0.215598141 | 0.735404165 | 0.092269162 | 0.120905381 | 0.387074105 |
|  |  | BTLA | 0.045996125 | 0.012069536 | 0.02893171 | 0.038491487 | 0.059990881 | 0.009653764 | 0.025940806 | 0.030432804 | 0.063884063 | 0.052267992 | 0.086413015 | 0.153479855 | 0 | 0.064884533 | 0 |
|  | Transcript factors | ZNF683 | 3.129705525 | 0.614846743 | 0.613756947 | 0.233062192 | 0.565625729 | 0.35116268 | 0.709048079 | 0.669395191 | 0.766945371 | 0.184890004 | 1.551239991 | 3.311924641 | 6.521719411 | 0.053304904 | 2.078306467 |
|  |  | HOPX | 5.49052823 | 1.679748743 | 1.320338687 | 0.284025712 | 1.022628829 | 1.029866939 | 2.940691778 | 1.052797451 | 2.486768685 | 2.772835933 | 3.88702227 | 3.569126684 | 7.097416783 | 2.38297703 | 5.127984543 |
|  |  | TOX | 0.322302403 | 0.422311084 | 0.441911264 | 0.048295767 | 0.441469952 | 0.736362152 | 0.506039621 | 0.373803473 | 0.629089441 | 0.040742011 | 0.248144387 | 0.846857685 | 0.20209062 | 0.219431142 | 0.434820853 |
|  |  | HIF1A | 0.748920742 | 1.072608948 | 0.677230833 | 0.588165862 | 0.628562862 | 0.680956491 | 0.668535027 | 0.766576931 | 0.82138787 | 0.518556654 | 0.792715857 | 1.00606546 | 0.664535456 | 0.487972184 | 0.41489751 |
|  |  | TBX21 | 0.386696786 | 0.457056329 | 0.373577428 | 0.142634365 | 0.441441652 | 0.421032677 | 0.55999282 | 0.260059577 | 0.19734701 | 0.222628535 | 0.521808577 | 0.26813136 | 0.290633378 | 0.497788592 | 0.338090382 |
|  |  | EOMES | 0.125814949 | 0.120381394 | 0.519552617 | 0.113987163 | 0.454842147 | 0.886052194 | 0.69438702 | 1.08375182 | 0.493227646 | 0.611523586 | 0.337944601 | 0.069991179 | 0.133569911 | 0.534270678 | 0.26858417 |
|  | Proliferation | MKI67 | 0.054539951 | 0.010327527 | 0.013175878 | 0 | 0.077454534 | 0.0207142 | 0.093824767 | 0.020552655 | 0.03517749 | 0.016168541 | 0 | 0.060016693 | 0.069360874 | 0 | 0 |
|  |  | CDK1 | 0.080435145 | 0.054683514 | 0.037293788 | 0 | 0.048932537 | 0.142829656 | 0.140984862 | 0.076586497 | 0.239771344 | 0.030435334 | 0.097690315 | 0.018405631 | 0 | 0.054112554 | 0 |
|  |  | STMN1 | 0.822045891 | 0.726297433 | 0.510604288 | 0.433964254 | 0.385598914 | 0.926820833 | 1.620293314 | 0.682585269 | 0.933961402 | 0.43976878 | 0.587195674 | 0.284549192 | 0.401338602 | 0.470821916 | 1.167380647 |

**Table S6. The various expression genes of macrophages in Branch 1 and 2 by pseudotime analyses**

| **GeneID** | **Cluster** | **P_value** | **FDR** | **Symbol** | **Description** |
| --- | --- | --- | --- | --- | --- |
| ENSG00000130203 | 1 | 0 | 0 | APOE | apolipoprotein E [Source:HGNC Symbol;Acc:HGNC:613] |
| ENSG00000275385 | 2 | 0 | 0 | CCL18 | C-C motif chemokine ligand 18 [Source:HGNC Symbol;Acc:HGNC:10616] |
| ENSG00000143546 | 3 | 0 | 0 | S100A8 | S100 calcium binding protein A8 [Source:HGNC Symbol;Acc:HGNC:10498] |
| ENSG00000130208 | 2 | 3.12E-211 | 1.31E-207 | APOC1 | apolipoprotein C1 [Source:HGNC Symbol;Acc:HGNC:607] |
| ENSG00000250722 | 4 | 4.03E-164 | 1.36E-160 | SELENOP | selenoprotein P [Source:HGNC Symbol;Acc:HGNC:10751] |
| ENSG00000163220 | 3 | 3.82E-147 | 1.07E-143 | S100A9 | S100 calcium binding protein A9 [Source:HGNC Symbol;Acc:HGNC:10499] |
| ENSG00000104918 | 2 | 3.37E-128 | 8.11E-125 | RETN | resistin [Source:HGNC Symbol;Acc:HGNC:20389] |
| ENSG00000165949 | 5 | 3.14E-125 | 6.62E-122 | IFI27 | interferon alpha inducible protein 27 [Source:HGNC Symbol;Acc:HGNC:5397] |
| ENSG00000085265 | 3 | 1.14E-122 | 2.13E-119 | FCN1 | ficolin 1 [Source:HGNC Symbol;Acc:HGNC:3623] |
| ENSG00000173369 | 5 | 4.04E-116 | 6.81E-113 | C1QB | complement C1q B chain [Source:HGNC Symbol;Acc:HGNC:1242] |
| ENSG00000173372 | 5 | 2.98E-114 | 4.57E-111 | C1QA | complement C1q A chain [Source:HGNC Symbol;Acc:HGNC:1241] |
| ENSG00000136235 | 1 | 8.96E-106 | 1.26E-102 | GPNMB | glycoprotein nmb [Source:HGNC Symbol;Acc:HGNC:4462] |
| ENSG00000164265 | 1 | 2.52E-96 | 3.27E-93 | SCGB3A2 | secretoglobin family 3A member 2 [Source:HGNC Symbol;Acc:HGNC:18391] |
| ENSG00000169442 | 2 | 6.14E-96 | 7.39E-93 | CD52 | CD52 molecule [Source:HGNC Symbol;Acc:HGNC:1804] |
| ENSG00000275302 | 4 | 2.25E-94 | 2.53E-91 | CCL4 | C-C motif chemokine ligand 4 [Source:HGNC Symbol;Acc:HGNC:10630] |
| ENSG00000117984 | 2 | 2.33E-93 | 2.45E-90 | CTSD | cathepsin D [Source:HGNC Symbol;Acc:HGNC:2529] |
| ENSG00000105223 | 5 | 6.07E-92 | 6.02E-89 | PLD3 | phospholipase D family member 3 [Source:HGNC Symbol;Acc:HGNC:17158] |
| ENSG00000129538 | 4 | 2.07E-91 | 1.94E-88 | RNASE1 | ribonuclease A family member 1, pancreatic [Source:HGNC Symbol;Acc:HGNC:10044] |
| ENSG00000100600 | 4 | 6.66E-89 | 5.91E-86 | LGMN | legumain [Source:HGNC Symbol;Acc:HGNC:9472] |
| ENSG00000087086 | 2 | 6.26E-88 | 5.28E-85 | FTL | ferritin light chain [Source:HGNC Symbol;Acc:HGNC:3999] |
| ENSG00000176046 | 2 | 8.04E-87 | 6.45E-84 | NUPR1 | nuclear protein 1, transcriptional regulator [Source:HGNC Symbol;Acc:HGNC:29990] |
| ENSG00000277632 | 4 | 2.51E-79 | 1.92E-76 | CCL3 | C-C motif chemokine ligand 3 [Source:HGNC Symbol;Acc:HGNC:10627] |
| ENSG00000181374 | 4 | 2.18E-78 | 1.60E-75 | CCL13 | C-C motif chemokine ligand 13 [Source:HGNC Symbol;Acc:HGNC:10611] |
| ENSG00000159189 | 4 | 3.06E-78 | 2.15E-75 | C1QC | complement C1q C chain [Source:HGNC Symbol;Acc:HGNC:1245] |
| ENSG00000138449 | 4 | 2.74E-76 | 1.85E-73 | SLC40A1 | solute carrier family 40 member 1 [Source:HGNC Symbol;Acc:HGNC:10909] |
| ENSG00000196154 | 2 | 1.25E-75 | 8.13E-73 | S100A4 | S100 calcium binding protein A4 [Source:HGNC Symbol;Acc:HGNC:10494] |
| ENSG00000107798 | 1 | 2.50E-73 | 1.56E-70 | LIPA | lipase A, lysosomal acid type [Source:HGNC Symbol;Acc:HGNC:6617] |
| ENSG00000262406 | 4 | 2.25E-70 | 1.35E-67 | MMP12 | matrix metallopeptidase 12 [Source:HGNC Symbol;Acc:HGNC:7158] |
| ENSG00000101160 | 5 | 2.92E-69 | 1.70E-66 | CTSZ | cathepsin Z [Source:HGNC Symbol;Acc:HGNC:2547] |
| ENSG00000197746 | 1 | 1.15E-63 | 6.47E-61 | PSAP | prosaposin [Source:HGNC Symbol;Acc:HGNC:9498] |
| ENSG00000187193 | 1 | 8.03E-58 | 4.37E-55 | MT1X | metallothionein 1X [Source:HGNC Symbol;Acc:HGNC:7405] |
| ENSG00000196735 | 5 | 5.30E-57 | 2.79E-54 | HLA-DQA1 | major histocompatibility complex, class II, DQ alpha 1 [Source:HGNC Symbol;Acc:HGNC:4942] |
| ENSG00000102265 | 3 | 3.22E-56 | 1.64E-53 | TIMP1 | TIMP metallopeptidase inhibitor 1 [Source:HGNC Symbol;Acc:HGNC:11820] |
| ENSG00000142669 | 3 | 1.98E-55 | 9.81E-53 | SH3BGRL3 | SH3 domain binding glutamate rich protein like 3 [Source:HGNC Symbol;Acc:HGNC:15568] |
| ENSG00000164104 | 3 | 2.22E-54 | 1.07E-51 | HMGB2 | high mobility group box 2 [Source:HGNC Symbol;Acc:HGNC:5000] |
| ENSG00000090382 | 3 | 9.56E-54 | 4.48E-51 | LYZ | lysozyme [Source:HGNC Symbol;Acc:HGNC:6740] |
| ENSG00000197061 | 3 | 4.40E-52 | 2.01E-49 | HIST1H4C | histone cluster 1 H4 family member c [Source:HGNC Symbol;Acc:HGNC:4787] |
| ENSG00000038427 | 3 | 4.13E-51 | 1.83E-48 | VCAN | versican [Source:HGNC Symbol;Acc:HGNC:2464] |
| ENSG00000165140 | 2 | 2.43E-50 | 1.02E-47 | FBP1 | fructose-bisphosphatase 1 [Source:HGNC Symbol;Acc:HGNC:3606] |
| ENSG00000134333 | 3 | 2.41E-50 | 1.02E-47 | LDHA | lactate dehydrogenase A [Source:HGNC Symbol;Acc:HGNC:6535] |
| ENSG00000165457 | 4 | 6.84E-49 | 2.81E-46 | FOLR2 | folate receptor beta [Source:HGNC Symbol;Acc:HGNC:3793] |
| ENSG00000197766 | 2 | 8.47E-49 | 3.40E-46 | CFD | complement factor D [Source:HGNC Symbol;Acc:HGNC:2771] |
| ENSG00000163736 | 2 | 1.93E-48 | 7.57E-46 | PPBP | pro-platelet basic protein [Source:HGNC Symbol;Acc:HGNC:9240] |
| ENSG00000164032 | 3 | 9.32E-48 | 3.57E-45 | H2AFZ | H2A histone family member Z [Source:HGNC Symbol;Acc:HGNC:4741] |
| ENSG00000120738 | 5 | 9.07E-47 | 3.40E-44 | EGR1 | early growth response 1 [Source:HGNC Symbol;Acc:HGNC:3238] |
| ENSG00000169429 | 4 | 2.28E-46 | 8.37E-44 | CXCL8 | C-X-C motif chemokine ligand 8 [Source:HGNC Symbol;Acc:HGNC:6025] |
| ENSG00000102575 | 2 | 1.49E-45 | 5.36E-43 | ACP5 | acid phosphatase 5, tartrate resistant [Source:HGNC Symbol;Acc:HGNC:124] |
| ENSG00000276070 | 4 | 2.70E-45 | 9.47E-43 | CCL4L2 | C-C motif chemokine ligand 4 like 2 [Source:HGNC Symbol;Acc:HGNC:24066] |
| ENSG00000198502 | 5 | 1.06E-43 | 3.66E-41 | HLA-DRB5 | major histocompatibility complex, class II, DR beta 5 [Source:HGNC Symbol;Acc:HGNC:4953] |
| ENSG00000081041 | 5 | 2.47E-43 | 8.32E-41 | CXCL2 | C-X-C motif chemokine ligand 2 [Source:HGNC Symbol;Acc:HGNC:4603] |
| ENSG00000197747 | 2 | 8.41E-43 | 2.78E-40 | S100A10 | S100 calcium binding protein A10 [Source:HGNC Symbol;Acc:HGNC:10487] |
| ENSG00000137331 | 4 | 1.06E-42 | 3.43E-40 | IER3 | immediate early response 3 [Source:HGNC Symbol;Acc:HGNC:5392] |
| ENSG00000197956 | 2 | 2.41E-42 | 7.65E-40 | S100A6 | S100 calcium binding protein A6 [Source:HGNC Symbol;Acc:HGNC:10496] |
| ENSG00000198830 | 3 | 1.14E-41 | 3.56E-39 | HMGN2 | high mobility group nucleosomal binding domain 2 [Source:HGNC Symbol;Acc:HGNC:4986] |
| ENSG00000123416 | 3 | 7.52E-41 | 2.31E-38 | TUBA1B | tubulin alpha 1b [Source:HGNC Symbol;Acc:HGNC:18809] |
| ENSG00000183019 | 2 | 4.52E-40 | 1.36E-37 | MCEMP1 | mast cell expressed membrane protein 1 [Source:HGNC Symbol;Acc:HGNC:27291] |
| ENSG00000138755 | 3 | 5.14E-40 | 1.52E-37 | CXCL9 | C-X-C motif chemokine ligand 9 [Source:HGNC Symbol;Acc:HGNC:7098] |
| ENSG00000163734 | 2 | 9.63E-40 | 2.80E-37 | CXCL3 | C-X-C motif chemokine ligand 3 [Source:HGNC Symbol;Acc:HGNC:4604] |
| ENSG00000175899 | 4 | 9.81E-40 | 2.80E-37 | A2M | alpha-2-macroglobulin [Source:HGNC Symbol;Acc:HGNC:7] |
| ENSG00000166803 | 3 | 2.33E-38 | 6.53E-36 | PCLAF | PCNA clamp associated factor [Source:HGNC Symbol;Acc:HGNC:28961] |
| ENSG00000100979 | 4 | 1.93E-37 | 5.32E-35 | PLTP | phospholipid transfer protein [Source:HGNC Symbol;Acc:HGNC:9093] |
| ENSG00000111640 | 3 | 3.47E-37 | 9.43E-35 | GAPDH | glyceraldehyde-3-phosphate dehydrogenase [Source:HGNC Symbol;Acc:HGNC:4141] |
| ENSG00000169715 | 1 | 8.15E-37 | 2.15E-34 | MT1E | metallothionein 1E [Source:HGNC Symbol;Acc:HGNC:7397] |
| ENSG00000241343 | 3 | 8.09E-37 | 2.15E-34 | RPL36A | ribosomal protein L36a [Source:HGNC Symbol;Acc:HGNC:10359] |
| ENSG00000187514 | 3 | 1.64E-36 | 4.25E-34 | PTMA | prothymosin alpha [Source:HGNC Symbol;Acc:HGNC:9623] |
| ENSG00000182774 | 3 | 3.33E-36 | 8.50E-34 | RPS17 | ribosomal protein S17 [Source:HGNC Symbol;Acc:HGNC:10397] |
| ENSG00000019169 | 2 | 2.08E-35 | 5.24E-33 | MARCO | macrophage receptor with collagenous structure [Source:HGNC Symbol;Acc:HGNC:6895] |
| ENSG00000169245 | 3 | 4.43E-35 | 1.10E-32 | CXCL10 | C-X-C motif chemokine ligand 10 [Source:HGNC Symbol;Acc:HGNC:10637] |
| ENSG00000103187 | 3 | 1.75E-34 | 4.29E-32 | COTL1 | coactosin like F-actin binding protein 1 [Source:HGNC Symbol;Acc:HGNC:18304] |
| ENSG00000145287 | 3 | 4.70E-34 | 1.13E-31 | PLAC8 | placenta specific 8 [Source:HGNC Symbol;Acc:HGNC:19254] |
| ENSG00000109321 | 3 | 9.07E-34 | 2.15E-31 | AREG | amphiregulin [Source:HGNC Symbol;Acc:HGNC:651] |
| ENSG00000277443 | 4 | 1.17E-33 | 2.74E-31 | MARCKS | myristoylated alanine rich protein kinase C substrate [Source:HGNC Symbol;Acc:HGNC:6759] |
| ENSG00000136156 | 4 | 1.28E-33 | 2.95E-31 | ITM2B | integral membrane protein 2B [Source:HGNC Symbol;Acc:HGNC:6174] |
| ENSG00000140988 | 3 | 1.69E-33 | 3.85E-31 | RPS2 | ribosomal protein S2 [Source:HGNC Symbol;Acc:HGNC:10404] |
| ENSG00000147604 | 3 | 2.98E-33 | 6.70E-31 | RPL7 | ribosomal protein L7 [Source:HGNC Symbol;Acc:HGNC:10363] |
| ENSG00000075624 | 3 | 7.15E-33 | 1.59E-30 | ACTB | actin beta [Source:HGNC Symbol;Acc:HGNC:132] |
| ENSG00000196230 | 3 | 8.44E-33 | 1.85E-30 | TUBB | tubulin beta class I [Source:HGNC Symbol;Acc:HGNC:20778] |
| ENSG00000153071 | 5 | 5.30E-32 | 1.15E-29 | DAB2 | DAB2, clathrin adaptor protein [Source:HGNC Symbol;Acc:HGNC:2662] |
| ENSG00000125144 | 1 | 5.46E-32 | 1.17E-29 | MT1G | metallothionein 1G [Source:HGNC Symbol;Acc:HGNC:7399] |
| ENSG00000276085 | 4 | 1.18E-31 | 2.48E-29 | CCL3L1 | C-C motif chemokine ligand 3 like 1 [Source:HGNC Symbol;Acc:HGNC:10628] |
| ENSG00000245532 | 1 | 2.75E-31 | 5.73E-29 | NEAT1 | nuclear paraspeckle assembly transcript 1 (non-protein coding) [Source:HGNC Symbol;Acc:HGNC:30815] |
| ENSG00000121552 | 3 | 4.57E-31 | 9.39E-29 | CSTA | cystatin A [Source:HGNC Symbol;Acc:HGNC:2481] |
| ENSG00000133063 | 1 | 5.23E-31 | 1.06E-28 | CHIT1 | chitinase 1 [Source:HGNC Symbol;Acc:HGNC:1936] |
| ENSG00000231500 | 3 | 8.14E-31 | 1.63E-28 | RPS18 | ribosomal protein S18 [Source:HGNC Symbol;Acc:HGNC:10401] |
| ENSG00000211592 | 4 | 1.14E-30 | 2.26E-28 | IGKC | immunoglobulin kappa constant [Source:HGNC Symbol;Acc:HGNC:5716] |
| MT-RNR1 | 4 | 3.04E-30 | 5.97E-28 | MT-RNR1 | - |
| ENSG00000072274 | 2 | 5.29E-30 | 1.03E-27 | TFRC | transferrin receptor [Source:HGNC Symbol;Acc:HGNC:11763] |
| ENSG00000196743 | 1 | 1.66E-29 | 3.18E-27 | GM2A | GM2 ganglioside activator [Source:HGNC Symbol;Acc:HGNC:4367] |
| ENSG00000105640 | 3 | 3.01E-29 | 5.69E-27 | RPL18A | ribosomal protein L18a [Source:HGNC Symbol;Acc:HGNC:10311] |
| ENSG00000161016 | 3 | 3.86E-29 | 7.22E-27 | RPL8 | ribosomal protein L8 [Source:HGNC Symbol;Acc:HGNC:10368] |
| ENSG00000074800 | 3 | 8.03E-29 | 1.49E-26 | ENO1 | enolase 1 [Source:HGNC Symbol;Acc:HGNC:3350] |
| ENSG00000111669 | 3 | 9.69E-29 | 1.78E-26 | TPI1 | triosephosphate isomerase 1 [Source:HGNC Symbol;Acc:HGNC:12009] |
| ENSG00000179163 | 5 | 5.57E-28 | 9.99E-26 | FUCA1 | alpha-L-fucosidase 1 [Source:HGNC Symbol;Acc:HGNC:4006] |
| ENSG00000240972 | 3 | 5.52E-28 | 9.99E-26 | MIF | macrophage migration inhibitory factor [Source:HGNC Symbol;Acc:HGNC:7097] |
| ENSG00000171858 | 3 | 5.97E-28 | 1.06E-25 | RPS21 | ribosomal protein S21 [Source:HGNC Symbol;Acc:HGNC:10409] |
| ENSG00000019582 | 4 | 6.61E-28 | 1.16E-25 | CD74 | CD74 molecule [Source:HGNC Symbol;Acc:HGNC:1697] |
| ENSG00000175063 | 3 | 3.77E-27 | 6.55E-25 | UBE2C | ubiquitin conjugating enzyme E2 C [Source:HGNC Symbol;Acc:HGNC:15937] |
| ENSG00000117632 | 3 | 4.10E-27 | 7.05E-25 | STMN1 | stathmin 1 [Source:HGNC Symbol;Acc:HGNC:6510] |
| ENSG00000118849 | 1 | 5.39E-27 | 9.18E-25 | RARRES1 | retinoic acid receptor responder 1 [Source:HGNC Symbol;Acc:HGNC:9867] |
| ENSG00000108518 | 3 | 5.94E-27 | 1.00E-24 | PFN1 | profilin 1 [Source:HGNC Symbol;Acc:HGNC:8881] |
| ENSG00000135047 | 1 | 1.53E-26 | 2.56E-24 | CTSL | cathepsin L [Source:HGNC Symbol;Acc:HGNC:2537] |
| ENSG00000136942 | 3 | 1.77E-26 | 2.93E-24 | RPL35 | ribosomal protein L35 [Source:HGNC Symbol;Acc:HGNC:10344] |
| ENSG00000166920 | 3 | 1.86E-26 | 3.04E-24 | C15orf48 | chromosome 15 open reading frame 48 [Source:HGNC Symbol;Acc:HGNC:29898] |
| ENSG00000067082 | 4 | 2.11E-26 | 3.41E-24 | KLF6 | Kruppel like factor 6 [Source:HGNC Symbol;Acc:HGNC:2235] |
| ENSG00000189403 | 3 | 2.67E-26 | 4.29E-24 | HMGB1 | high mobility group box 1 [Source:HGNC Symbol;Acc:HGNC:4983] |
| ENSG00000142937 | 3 | 5.75E-26 | 9.15E-24 | RPS8 | ribosomal protein S8 [Source:HGNC Symbol;Acc:HGNC:10441] |
| MT-RNR2 | 4 | 1.19E-25 | 1.87E-23 | MT-RNR2 | - |
| ENSG00000109861 | 5 | 2.33E-25 | 3.63E-23 | CTSC | cathepsin C [Source:HGNC Symbol;Acc:HGNC:2528] |
| ENSG00000156113 | 2 | 3.28E-25 | 5.07E-23 | KCNMA1 | potassium calcium-activated channel subfamily M alpha 1 [Source:HGNC Symbol;Acc:HGNC:6284] |
| ENSG00000205362 | 1 | 3.47E-25 | 5.31E-23 | MT1A | metallothionein 1A [Source:HGNC Symbol;Acc:HGNC:7393] |
| ENSG00000164611 | 3 | 7.38E-25 | 1.12E-22 | PTTG1 | pituitary tumor-transforming 1 [Source:HGNC Symbol;Acc:HGNC:9690] |
| ENSG00000118785 | 4 | 1.24E-24 | 1.86E-22 | SPP1 | secreted phosphoprotein 1 [Source:HGNC Symbol;Acc:HGNC:11255] |
| ENSG00000168028 | 3 | 1.31E-24 | 1.96E-22 | RPSA | ribosomal protein SA [Source:HGNC Symbol;Acc:HGNC:6502] |
| ENSG00000100911 | 3 | 1.50E-24 | 2.21E-22 | PSME2 | proteasome activator subunit 2 [Source:HGNC Symbol;Acc:HGNC:9569] |
| ENSG00000089157 | 3 | 2.20E-24 | 3.23E-22 | RPLP0 | ribosomal protein lateral stalk subunit P0 [Source:HGNC Symbol;Acc:HGNC:10371] |
| ENSG00000176890 | 3 | 3.77E-24 | 5.47E-22 | TYMS | thymidylate synthetase [Source:HGNC Symbol;Acc:HGNC:12441] |
| ENSG00000173207 | 3 | 4.39E-24 | 6.33E-22 | CKS1B | CDC28 protein kinase regulatory subunit 1B [Source:HGNC Symbol;Acc:HGNC:19083] |
| ENSG00000198804 | 5 | 5.00E-24 | 7.15E-22 | MT-CO1 | mitochondrially encoded cytochrome c oxidase I [Source:HGNC Symbol;Acc:HGNC:7419] |
| ENSG00000198763 | 2 | 5.41E-24 | 7.67E-22 | MT-ND2 | mitochondrially encoded NADH:ubiquinone oxidoreductase core subunit 2 [Source:HGNC Symbol;Acc:HGNC:7456] |
| ENSG00000137491 | 4 | 6.42E-24 | 9.02E-22 | SLCO2B1 | solute carrier organic anion transporter family member 2B1 [Source:HGNC Symbol;Acc:HGNC:10962] |
| ENSG00000198727 | 2 | 6.73E-24 | 9.37E-22 | MT-CYB | mitochondrially encoded cytochrome b [Source:HGNC Symbol;Acc:HGNC:7427] |
| ENSG00000265681 | 3 | 2.60E-23 | 3.59E-21 | RPL17 | ribosomal protein L17 [Source:HGNC Symbol;Acc:HGNC:10307] |
| ENSG00000229117 | 3 | 3.37E-23 | 4.62E-21 | RPL41 | ribosomal protein L41 [Source:HGNC Symbol;Acc:HGNC:10354] |
| ENSG00000125148 | 1 | 3.70E-23 | 5.03E-21 | MT2A | metallothionein 2A [Source:HGNC Symbol;Acc:HGNC:7406] |
| ENSG00000137154 | 3 | 4.36E-23 | 5.88E-21 | RPS6 | ribosomal protein S6 [Source:HGNC Symbol;Acc:HGNC:10429] |
| ENSG00000034510 | 3 | 5.30E-23 | 7.10E-21 | TMSB10 | thymosin beta 10 [Source:HGNC Symbol;Acc:HGNC:11879] |
| ENSG00000205364 | 1 | 9.92E-23 | 1.32E-20 | MT1M | metallothionein 1M [Source:HGNC Symbol;Acc:HGNC:14296] |
| ENSG00000038945 | 5 | 1.03E-22 | 1.35E-20 | MSR1 | macrophage scavenger receptor 1 [Source:HGNC Symbol;Acc:HGNC:7376] |
| ENSG00000136810 | 2 | 1.04E-22 | 1.35E-20 | TXN | thioredoxin [Source:HGNC Symbol;Acc:HGNC:12435] |
| ENSG00000185215 | 2 | 1.15E-22 | 1.49E-20 | TNFAIP2 | TNF alpha induced protein 2 [Source:HGNC Symbol;Acc:HGNC:11895] |
| ENSG00000112149 | 4 | 1.49E-22 | 1.92E-20 | CD83 | CD83 molecule [Source:HGNC Symbol;Acc:HGNC:1703] |
| ENSG00000198938 | 5 | 2.25E-22 | 2.87E-20 | MT-CO3 | mitochondrially encoded cytochrome c oxidase III [Source:HGNC Symbol;Acc:HGNC:7422] |
| ENSG00000102007 | 3 | 3.09E-22 | 3.92E-20 | PLP2 | proteolipid protein 2 [Source:HGNC Symbol;Acc:HGNC:9087] |
| ENSG00000198755 | 3 | 4.04E-22 | 5.05E-20 | RPL10A | ribosomal protein L10a [Source:HGNC Symbol;Acc:HGNC:10299] |
| ENSG00000144713 | 3 | 4.03E-22 | 5.05E-20 | RPL32 | ribosomal protein L32 [Source:HGNC Symbol;Acc:HGNC:10336] |
| ENSG00000133048 | 1 | 5.56E-22 | 6.89E-20 | CHI3L1 | chitinase 3 like 1 [Source:HGNC Symbol;Acc:HGNC:1932] |
| ENSG00000138326 | 3 | 6.12E-22 | 7.53E-20 | RPS24 | ribosomal protein S24 [Source:HGNC Symbol;Acc:HGNC:10411] |
| ENSG00000166278 | 5 | 9.37E-22 | 1.14E-19 | C2 | complement C2 [Source:HGNC Symbol;Acc:HGNC:1248] |
| ENSG00000131747 | 3 | 1.14E-21 | 1.38E-19 | TOP2A | DNA topoisomerase II alpha [Source:HGNC Symbol;Acc:HGNC:11989] |
| ENSG00000217555 | 4 | 1.36E-21 | 1.64E-19 | CKLF | chemokine like factor [Source:HGNC Symbol;Acc:HGNC:13253] |
| ENSG00000197728 | 3 | 2.03E-21 | 2.43E-19 | RPS26 | ribosomal protein S26 [Source:HGNC Symbol;Acc:HGNC:10414] |
| HLA-DRB6 | 3 | 2.20E-21 | 2.62E-19 | HLA-DRB6 | - |
| ENSG00000125740 | 4 | 2.64E-21 | 3.12E-19 | FOSB | FosB proto-oncogene, AP-1 transcription factor subunit [Source:HGNC Symbol;Acc:HGNC:3797] |
| ENSG00000115414 | 2 | 2.87E-21 | 3.36E-19 | FN1 | fibronectin 1 [Source:HGNC Symbol;Acc:HGNC:3778] |
| ENSG00000122026 | 3 | 3.68E-21 | 4.28E-19 | RPL21 | ribosomal protein L21 [Source:HGNC Symbol;Acc:HGNC:10313] |
| ENSG00000110203 | 2 | 4.52E-21 | 5.22E-19 | FOLR3 | folate receptor 3 [Source:HGNC Symbol;Acc:HGNC:3795] |
| ENSG00000165092 | 2 | 5.97E-21 | 6.85E-19 | ALDH1A1 | aldehyde dehydrogenase 1 family member A1 [Source:HGNC Symbol;Acc:HGNC:402] |
| ENSG00000197756 | 3 | 6.27E-21 | 7.14E-19 | RPL37A | ribosomal protein L37a [Source:HGNC Symbol;Acc:HGNC:10348] |
| ENSG00000135486 | 3 | 8.46E-21 | 9.57E-19 | HNRNPA1 | heterogeneous nuclear ribonucleoprotein A1 [Source:HGNC Symbol;Acc:HGNC:5031] |
| ENSG00000171848 | 3 | 1.06E-20 | 1.20E-18 | RRM2 | ribonucleotide reductase regulatory subunit M2 [Source:HGNC Symbol;Acc:HGNC:10452] |
| ENSG00000115828 | 3 | 1.24E-20 | 1.38E-18 | QPCT | glutaminyl-peptide cyclotransferase [Source:HGNC Symbol;Acc:HGNC:9753] |
| ENSG00000146070 | 1 | 1.39E-20 | 1.54E-18 | PLA2G7 | phospholipase A2 group VII [Source:HGNC Symbol;Acc:HGNC:9040] |
| ENSG00000100097 | 3 | 1.64E-20 | 1.80E-18 | LGALS1 | galectin 1 [Source:HGNC Symbol;Acc:HGNC:6561] |
| ENSG00000161970 | 3 | 1.64E-20 | 1.80E-18 | RPL26 | ribosomal protein L26 [Source:HGNC Symbol;Acc:HGNC:10327] |
| ENSG00000198888 | 2 | 1.82E-20 | 1.98E-18 | MT-ND1 | mitochondrially encoded NADH:ubiquinone oxidoreductase core subunit 1 [Source:HGNC Symbol;Acc:HGNC:7455] |
| ENSG00000132965 | 3 | 2.03E-20 | 2.20E-18 | ALOX5AP | arachidonate 5-lipoxygenase activating protein [Source:HGNC Symbol;Acc:HGNC:436] |
| ENSG00000100292 | 4 | 4.97E-20 | 5.33E-18 | HMOX1 | heme oxygenase 1 [Source:HGNC Symbol;Acc:HGNC:5013] |
| ENSG00000177606 | 5 | 5.81E-20 | 6.20E-18 | JUN | Jun proto-oncogene, AP-1 transcription factor subunit [Source:HGNC Symbol;Acc:HGNC:6204] |
| ENSG00000115268 | 3 | 9.31E-20 | 9.87E-18 | RPS15 | ribosomal protein S15 [Source:HGNC Symbol;Acc:HGNC:10388] |
| ENSG00000198242 | 3 | 1.13E-19 | 1.19E-17 | RPL23A | ribosomal protein L23a [Source:HGNC Symbol;Acc:HGNC:10317] |
| ENSG00000114391 | 3 | 1.21E-19 | 1.27E-17 | RPL24 | ribosomal protein L24 [Source:HGNC Symbol;Acc:HGNC:10325] |
| ENSG00000132341 | 3 | 1.78E-19 | 1.86E-17 | RAN | RAN, member RAS oncogene family [Source:HGNC Symbol;Acc:HGNC:9846] |
| ENSG00000167552 | 3 | 1.82E-19 | 1.88E-17 | TUBA1A | tubulin alpha 1a [Source:HGNC Symbol;Acc:HGNC:20766] |
| ENSG00000102879 | 3 | 2.02E-19 | 2.08E-17 | CORO1A | coronin 1A [Source:HGNC Symbol;Acc:HGNC:2252] |
| ENSG00000158481 | 3 | 2.16E-19 | 2.21E-17 | CD1C | CD1c molecule [Source:HGNC Symbol;Acc:HGNC:1636] |
| ENSG00000198712 | 5 | 2.54E-19 | 2.56E-17 | MT-CO2 | mitochondrially encoded cytochrome c oxidase II [Source:HGNC Symbol;Acc:HGNC:7421] |
| ENSG00000149131 | 2 | 2.54E-19 | 2.56E-17 | SERPING1 | serpin family G member 1 [Source:HGNC Symbol;Acc:HGNC:1228] |
| ENSG00000105372 | 3 | 5.14E-19 | 5.16E-17 | RPS19 | ribosomal protein S19 [Source:HGNC Symbol;Acc:HGNC:10402] |
| ENSG00000135678 | 1 | 5.68E-19 | 5.67E-17 | CPM | carboxypeptidase M [Source:HGNC Symbol;Acc:HGNC:2311] |
| ENSG00000104870 | 5 | 7.56E-19 | 7.50E-17 | FCGRT | Fc fragment of IgG receptor and transporter [Source:HGNC Symbol;Acc:HGNC:3621] |
| ENSG00000169230 | 3 | 7.93E-19 | 7.82E-17 | PRELID1 | PRELI domain containing 1 [Source:HGNC Symbol;Acc:HGNC:30255] |
| ENSG00000182899 | 3 | 1.09E-18 | 1.07E-16 | RPL35A | ribosomal protein L35a [Source:HGNC Symbol;Acc:HGNC:10345] |
| ENSG00000185339 | 5 | 1.32E-18 | 1.29E-16 | TCN2 | transcobalamin 2 [Source:HGNC Symbol;Acc:HGNC:11653] |
| ENSG00000108700 | 4 | 1.60E-18 | 1.55E-16 | CCL8 | C-C motif chemokine ligand 8 [Source:HGNC Symbol;Acc:HGNC:10635] |
| ENSG00000164733 | 1 | 1.72E-18 | 1.66E-16 | CTSB | cathepsin B [Source:HGNC Symbol;Acc:HGNC:2527] |
| ENSG00000197249 | 2 | 3.82E-18 | 3.66E-16 | SERPINA1 | serpin family A member 1 [Source:HGNC Symbol;Acc:HGNC:8941] |
| ENSG00000143162 | 4 | 4.09E-18 | 3.89E-16 | CREG1 | cellular repressor of E1A stimulated genes 1 [Source:HGNC Symbol;Acc:HGNC:2351] |
| ENSG00000167526 | 3 | 4.57E-18 | 4.33E-16 | RPL13 | ribosomal protein L13 [Source:HGNC Symbol;Acc:HGNC:10303] |
| ENSG00000131469 | 3 | 5.05E-18 | 4.75E-16 | RPL27 | ribosomal protein L27 [Source:HGNC Symbol;Acc:HGNC:10328] |
| ENSG00000100316 | 3 | 5.21E-18 | 4.88E-16 | RPL3 | ribosomal protein L3 [Source:HGNC Symbol;Acc:HGNC:10332] |
| ENSG00000102144 | 3 | 6.76E-18 | 6.29E-16 | PGK1 | phosphoglycerate kinase 1 [Source:HGNC Symbol;Acc:HGNC:8896] |
| ENSG00000108828 | 2 | 6.87E-18 | 6.36E-16 | VAT1 | vesicle amine transport 1 [Source:HGNC Symbol;Acc:HGNC:16919] |
| ENSG00000177954 | 3 | 7.48E-18 | 6.89E-16 | RPS27 | ribosomal protein S27 [Source:HGNC Symbol;Acc:HGNC:10416] |
| ENSG00000198918 | 3 | 7.59E-18 | 6.95E-16 | RPL39 | ribosomal protein L39 [Source:HGNC Symbol;Acc:HGNC:10350] |
| ENSG00000124614 | 3 | 7.90E-18 | 7.20E-16 | RPS10 | ribosomal protein S10 [Source:HGNC Symbol;Acc:HGNC:10383] |
| ENSG00000112306 | 3 | 8.89E-18 | 8.06E-16 | RPS12 | ribosomal protein S12 [Source:HGNC Symbol;Acc:HGNC:10385] |
| ENSG00000099860 | 4 | 1.05E-17 | 9.48E-16 | GADD45B | growth arrest and DNA damage inducible beta [Source:HGNC Symbol;Acc:HGNC:4096] |
| ENSG00000188042 | 4 | 1.19E-17 | 1.06E-15 | ARL4C | ADP ribosylation factor like GTPase 4C [Source:HGNC Symbol;Acc:HGNC:698] |
| ENSG00000168484 | 4 | 1.36E-17 | 1.22E-15 | SFTPC | surfactant protein C [Source:HGNC Symbol;Acc:HGNC:10802] |
| ENSG00000089009 | 3 | 1.42E-17 | 1.26E-15 | RPL6 | ribosomal protein L6 [Source:HGNC Symbol;Acc:HGNC:10362] |
| ENSG00000083845 | 3 | 1.84E-17 | 1.63E-15 | RPS5 | ribosomal protein S5 [Source:HGNC Symbol;Acc:HGNC:10426] |
| ENSG00000110651 | 5 | 1.85E-17 | 1.63E-15 | CD81 | CD81 molecule [Source:HGNC Symbol;Acc:HGNC:1701] |
| ENSG00000163131 | 1 | 2.03E-17 | 1.77E-15 | CTSS | cathepsin S [Source:HGNC Symbol;Acc:HGNC:2545] |
| ENSG00000175315 | 3 | 2.63E-17 | 2.28E-15 | CST6 | cystatin E/M [Source:HGNC Symbol;Acc:HGNC:2478] |
| ENSG00000198840 | 5 | 3.34E-17 | 2.88E-15 | MT-ND3 | mitochondrially encoded NADH:ubiquinone oxidoreductase core subunit 3 [Source:HGNC Symbol;Acc:HGNC:7458] |
| ENSG00000130255 | 3 | 4.44E-17 | 3.82E-15 | RPL36 | ribosomal protein L36 [Source:HGNC Symbol;Acc:HGNC:13631] |
| ENSG00000198899 | 5 | 5.56E-17 | 4.76E-15 | MT-ATP6 | mitochondrially encoded ATP synthase membrane subunit 6 [Source:HGNC Symbol;Acc:HGNC:7414] |
| ENSG00000204482 | 3 | 5.81E-17 | 4.95E-15 | LST1 | leukocyte specific transcript 1 [Source:HGNC Symbol;Acc:HGNC:14189] |
| ENSG00000177600 | 3 | 6.26E-17 | 5.27E-15 | RPLP2 | ribosomal protein lateral stalk subunit P2 [Source:HGNC Symbol;Acc:HGNC:10377] |
| ENSG00000285441 | 4 | 6.23E-17 | 5.27E-15 | SOD2 | superoxide dismutase 2 [Source:NCBI gene;Acc:6648] |
| ENSG00000231389 | 4 | 7.00E-17 | 5.84E-15 | HLA-DPA1 | major histocompatibility complex, class II, DP alpha 1 [Source:HGNC Symbol;Acc:HGNC:4938] |
| ENSG00000180817 | 3 | 6.99E-17 | 5.84E-15 | PPA1 | pyrophosphatase (inorganic) 1 [Source:HGNC Symbol;Acc:HGNC:9226] |
| ENSG00000160789 | 2 | 7.85E-17 | 6.52E-15 | LMNA | lamin A/C [Source:HGNC Symbol;Acc:HGNC:6636] |
| ENSG00000076944 | 2 | 9.77E-17 | 8.07E-15 | STXBP2 | syntaxin binding protein 2 [Source:HGNC Symbol;Acc:HGNC:11445] |
| ENSG00000099901 | 3 | 1.20E-16 | 9.84E-15 | RANBP1 | RAN binding protein 1 [Source:HGNC Symbol;Acc:HGNC:9847] |
| ENSG00000163735 | 2 | 1.21E-16 | 9.92E-15 | CXCL5 | C-X-C motif chemokine ligand 5 [Source:HGNC Symbol;Acc:HGNC:10642] |
| ENSG00000182853 | 5 | 1.59E-16 | 1.29E-14 | VMO1 | vitelline membrane outer layer 1 homolog [Source:HGNC Symbol;Acc:HGNC:30387] |
| ENSG00000173915 | 3 | 2.25E-16 | 1.82E-14 | ATP5MD | ATP synthase membrane subunit DAPIT [Source:HGNC Symbol;Acc:HGNC:30889] |
| ENSG00000134419 | 3 | 2.45E-16 | 1.98E-14 | RPS15A | ribosomal protein S15a [Source:HGNC Symbol;Acc:HGNC:10389] |
| ENSG00000166927 | 2 | 2.68E-16 | 2.15E-14 | MS4A7 | membrane spanning 4-domains A7 [Source:HGNC Symbol;Acc:HGNC:13378] |
| ENSG00000179344 | 4 | 3.27E-16 | 2.61E-14 | HLA-DQB1 | major histocompatibility complex, class II, DQ beta 1 [Source:HGNC Symbol;Acc:HGNC:4944] |
| ENSG00000160888 | 4 | 3.41E-16 | 2.71E-14 | IER2 | immediate early response 2 [Source:HGNC Symbol;Acc:HGNC:28871] |
| ENSG00000111796 | 3 | 3.48E-16 | 2.75E-14 | KLRB1 | killer cell lectin like receptor B1 [Source:HGNC Symbol;Acc:HGNC:6373] |
| ENSG00000130522 | 5 | 3.63E-16 | 2.86E-14 | JUND | JunD proto-oncogene, AP-1 transcription factor subunit [Source:HGNC Symbol;Acc:HGNC:6206] |
| ENSG00000135929 | 2 | 4.27E-16 | 3.35E-14 | CYP27A1 | cytochrome P450 family 27 subfamily A member 1 [Source:HGNC Symbol;Acc:HGNC:2605] |
| ENSG00000149273 | 3 | 5.13E-16 | 4.00E-14 | RPS3 | ribosomal protein S3 [Source:HGNC Symbol;Acc:HGNC:10420] |
| ENSG00000176340 | 3 | 6.35E-16 | 4.93E-14 | COX8A | cytochrome c oxidase subunit 8A [Source:HGNC Symbol;Acc:HGNC:2294] |
| ENSG00000131174 | 3 | 6.55E-16 | 5.07E-14 | COX7B | cytochrome c oxidase subunit 7B [Source:HGNC Symbol;Acc:HGNC:2291] |
| ENSG00000239672 | 3 | 6.68E-16 | 5.14E-14 | NME1 | NME/NM23 nucleoside diphosphate kinase 1 [Source:HGNC Symbol;Acc:HGNC:7849] |
| ENSG00000100453 | 3 | 6.95E-16 | 5.33E-14 | GZMB | granzyme B [Source:HGNC Symbol;Acc:HGNC:4709] |
| ENSG00000196126 | 4 | 8.86E-16 | 6.76E-14 | HLA-DRB1 | major histocompatibility complex, class II, DR beta 1 [Source:HGNC Symbol;Acc:HGNC:4948] |
| ENSG00000118503 | 5 | 9.50E-16 | 7.22E-14 | TNFAIP3 | TNF alpha induced protein 3 [Source:HGNC Symbol;Acc:HGNC:11896] |
| ENSG00000108679 | 5 | 1.15E-15 | 8.67E-14 | LGALS3BP | galectin 3 binding protein [Source:HGNC Symbol;Acc:HGNC:6564] |
| ENSG00000113140 | 5 | 1.50E-15 | 1.13E-13 | SPARC | secreted protein acidic and cysteine rich [Source:HGNC Symbol;Acc:HGNC:11219] |
| ENSG00000146278 | 5 | 1.67E-15 | 1.25E-13 | PNRC1 | proline rich nuclear receptor coactivator 1 [Source:HGNC Symbol;Acc:HGNC:17278] |
| ENSG00000026025 | 2 | 1.68E-15 | 1.26E-13 | VIM | vimentin [Source:HGNC Symbol;Acc:HGNC:12692] |
| ENSG00000110700 | 3 | 1.84E-15 | 1.37E-13 | RPS13 | ribosomal protein S13 [Source:HGNC Symbol;Acc:HGNC:10386] |
| ENSG00000102970 | 3 | 1.87E-15 | 1.38E-13 | CCL17 | C-C motif chemokine ligand 17 [Source:HGNC Symbol;Acc:HGNC:10615] |
| ENSG00000142227 | 2 | 1.89E-15 | 1.39E-13 | EMP3 | epithelial membrane protein 3 [Source:HGNC Symbol;Acc:HGNC:3335] |
| ENSG00000162512 | 4 | 2.20E-15 | 1.61E-13 | SDC3 | syndecan 3 [Source:HGNC Symbol;Acc:HGNC:10660] |
| ENSG00000118181 | 3 | 2.69E-15 | 1.96E-13 | RPS25 | ribosomal protein S25 [Source:HGNC Symbol;Acc:HGNC:10413] |
| ENSG00000030582 | 2 | 3.62E-15 | 2.62E-13 | GRN | granulin precursor [Source:HGNC Symbol;Acc:HGNC:4601] |
| ENSG00000162244 | 3 | 3.61E-15 | 2.62E-13 | RPL29 | ribosomal protein L29 [Source:HGNC Symbol;Acc:HGNC:10331] |
| ENSG00000171659 | 4 | 4.64E-15 | 3.34E-13 | GPR34 | G protein-coupled receptor 34 [Source:HGNC Symbol;Acc:HGNC:4490] |
| ENSG00000137880 | 2 | 4.82E-15 | 3.46E-13 | GCHFR | GTP cyclohydrolase I feedback regulator [Source:HGNC Symbol;Acc:HGNC:4194] |
| ENSG00000132507 | 3 | 5.00E-15 | 3.57E-13 | EIF5A | eukaryotic translation initiation factor 5A [Source:HGNC Symbol;Acc:HGNC:3300] |
| ENSG00000186468 | 3 | 5.60E-15 | 3.99E-13 | RPS23 | ribosomal protein S23 [Source:HGNC Symbol;Acc:HGNC:10410] |
| ENSG00000163466 | 2 | 5.91E-15 | 4.18E-13 | ARPC2 | actin related protein 2/3 complex subunit 2 [Source:HGNC Symbol;Acc:HGNC:705] |
| ENSG00000109475 | 3 | 6.23E-15 | 4.40E-13 | RPL34 | ribosomal protein L34 [Source:HGNC Symbol;Acc:HGNC:10340] |
| ENSG00000025708 | 3 | 6.90E-15 | 4.85E-13 | TYMP | thymidine phosphorylase [Source:HGNC Symbol;Acc:HGNC:3148] |
| ENSG00000072571 | 3 | 6.94E-15 | 4.86E-13 | HMMR | hyaluronan mediated motility receptor [Source:HGNC Symbol;Acc:HGNC:5012] |
| ENSG00000233927 | 3 | 7.04E-15 | 4.91E-13 | RPS28 | ribosomal protein S28 [Source:HGNC Symbol;Acc:HGNC:10418] |
| ENSG00000170889 | 3 | 9.09E-15 | 6.31E-13 | RPS9 | ribosomal protein S9 [Source:HGNC Symbol;Acc:HGNC:10442] |
| ENSG00000145592 | 3 | 9.99E-15 | 6.91E-13 | RPL37 | ribosomal protein L37 [Source:HGNC Symbol;Acc:HGNC:10347] |
| ENSG00000205358 | 3 | 1.01E-14 | 6.97E-13 | MT1H | metallothionein 1H [Source:HGNC Symbol;Acc:HGNC:7400] |
| ENSG00000222041 | 3 | 1.03E-14 | 7.06E-13 | CYTOR | cytoskeleton regulator RNA [Source:HGNC Symbol;Acc:HGNC:28717] |
| ENSG00000095970 | 1 | 1.41E-14 | 9.65E-13 | TREM2 | triggering receptor expressed on myeloid cells 2 [Source:HGNC Symbol;Acc:HGNC:17761] |
| ENSG00000089685 | 3 | 1.61E-14 | 1.09E-12 | BIRC5 | baculoviral IAP repeat containing 5 [Source:HGNC Symbol;Acc:HGNC:593] |
| ENSG00000121769 | 2 | 1.87E-14 | 1.26E-12 | FABP3 | fatty acid binding protein 3 [Source:HGNC Symbol;Acc:HGNC:3557] |
| ENSG00000074416 | 2 | 1.97E-14 | 1.33E-12 | MGLL | monoglyceride lipase [Source:HGNC Symbol;Acc:HGNC:17038] |
| ENSG00000171314 | 3 | 2.03E-14 | 1.37E-12 | PGAM1 | phosphoglycerate mutase 1 [Source:HGNC Symbol;Acc:HGNC:8888] |
| ENSG00000221983 | 3 | 2.58E-14 | 1.73E-12 | UBA52 | ubiquitin A-52 residue ribosomal protein fusion product 1 [Source:HGNC Symbol;Acc:HGNC:12458] |
| ENSG00000213741 | 3 | 2.72E-14 | 1.81E-12 | RPS29 | ribosomal protein S29 [Source:HGNC Symbol;Acc:HGNC:10419] |
| ENSG00000145425 | 3 | 2.93E-14 | 1.94E-12 | RPS3A | ribosomal protein S3A [Source:HGNC Symbol;Acc:HGNC:10421] |
| ENSG00000172590 | 3 | 3.25E-14 | 2.15E-12 | MRPL52 | mitochondrial ribosomal protein L52 [Source:HGNC Symbol;Acc:HGNC:16655] |
| ENSG00000115008 | 2 | 3.42E-14 | 2.25E-12 | IL1A | interleukin 1 alpha [Source:HGNC Symbol;Acc:HGNC:5991] |
| ENSG00000162772 | 4 | 4.23E-14 | 2.78E-12 | ATF3 | activating transcription factor 3 [Source:HGNC Symbol;Acc:HGNC:785] |
| ENSG00000204257 | 4 | 5.73E-14 | 3.75E-12 | HLA-DMA | major histocompatibility complex, class II, DM alpha [Source:HGNC Symbol;Acc:HGNC:4934] |
| ENSG00000182287 | 3 | 6.26E-14 | 4.08E-12 | AP1S2 | adaptor related protein complex 1 subunit sigma 2 [Source:HGNC Symbol;Acc:HGNC:560] |
| ENSG00000067225 | 3 | 6.85E-14 | 4.44E-12 | PKM | pyruvate kinase M1/2 [Source:HGNC Symbol;Acc:HGNC:9021] |
| ENSG00000134028 | 4 | 7.19E-14 | 4.64E-12 | ADAMDEC1 | ADAM like decysin 1 [Source:HGNC Symbol;Acc:HGNC:16299] |
| ENSG00000090339 | 4 | 7.65E-14 | 4.92E-12 | ICAM1 | intercellular adhesion molecule 1 [Source:HGNC Symbol;Acc:HGNC:5344] |
| ENSG00000277734 | 3 | 7.67E-14 | 4.92E-12 | TRAC | T cell receptor alpha constant [Source:HGNC Symbol;Acc:HGNC:12029] |
| ENSG00000143226 | 4 | 1.16E-13 | 7.39E-12 | FCGR2A | Fc fragment of IgG receptor IIa [Source:HGNC Symbol;Acc:HGNC:3616] |
| ENSG00000105609 | 4 | 1.30E-13 | 8.30E-12 | LILRB5 | leukocyte immunoglobulin like receptor B5 [Source:HGNC Symbol;Acc:HGNC:6609] |
| ENSG00000204103 | 4 | 1.98E-13 | 1.26E-11 | MAFB | MAF bZIP transcription factor B [Source:HGNC Symbol;Acc:HGNC:6408] |
| ENSG00000104763 | 2 | 2.04E-13 | 1.29E-11 | ASAH1 | N-acylsphingosine amidohydrolase 1 [Source:HGNC Symbol;Acc:HGNC:735] |
| ENSG00000151474 | 5 | 2.51E-13 | 1.58E-11 | FRMD4A | FERM domain containing 4A [Source:HGNC Symbol;Acc:HGNC:25491] |
| ENSG00000137804 | 3 | 3.10E-13 | 1.94E-11 | NUSAP1 | nucleolar and spindle associated protein 1 [Source:HGNC Symbol;Acc:HGNC:18538] |
| ENSG00000171863 | 3 | 3.35E-13 | 2.09E-11 | RPS7 | ribosomal protein S7 [Source:HGNC Symbol;Acc:HGNC:10440] |
| ENSG00000093072 | 1 | 3.37E-13 | 2.10E-11 | ADA2 | adenosine deaminase 2 [Source:HGNC Symbol;Acc:HGNC:1839] |
| ENSG00000107317 | 1 | 3.51E-13 | 2.18E-11 | PTGDS | prostaglandin D2 synthase [Source:HGNC Symbol;Acc:HGNC:9592] |
| ENSG00000163041 | 3 | 3.70E-13 | 2.27E-11 | H3F3A | H3 histone family member 3A [Source:HGNC Symbol;Acc:HGNC:4764] |
| ENSG00000164405 | 3 | 3.69E-13 | 2.27E-11 | UQCRQ | ubiquinol-cytochrome c reductase complex III subunit VII [Source:HGNC Symbol;Acc:HGNC:29594] |
| ENSG00000035862 | 5 | 5.12E-13 | 3.14E-11 | TIMP2 | TIMP metallopeptidase inhibitor 2 [Source:HGNC Symbol;Acc:HGNC:11821] |
| ENSG00000140105 | 5 | 5.19E-13 | 3.17E-11 | WARS | tryptophanyl-tRNA synthetase [Source:HGNC Symbol;Acc:HGNC:12729] |
| ENSG00000123342 | 2 | 5.29E-13 | 3.22E-11 | MMP19 | matrix metallopeptidase 19 [Source:HGNC Symbol;Acc:HGNC:7165] |
| ENSG00000169248 | 3 | 5.53E-13 | 3.35E-11 | CXCL11 | C-X-C motif chemokine ligand 11 [Source:HGNC Symbol;Acc:HGNC:10638] |
| ENSG00000005893 | 5 | 5.58E-13 | 3.37E-11 | LAMP2 | lysosomal associated membrane protein 2 [Source:HGNC Symbol;Acc:HGNC:6501] |
| ENSG00000161921 | 2 | 6.10E-13 | 3.67E-11 | CXCL16 | C-X-C motif chemokine ligand 16 [Source:HGNC Symbol;Acc:HGNC:16642] |
| ENSG00000160213 | 2 | 8.53E-13 | 5.12E-11 | CSTB | cystatin B [Source:HGNC Symbol;Acc:HGNC:2482] |
| ENSG00000185896 | 2 | 8.67E-13 | 5.19E-11 | LAMP1 | lysosomal associated membrane protein 1 [Source:HGNC Symbol;Acc:HGNC:6499] |
| ENSG00000211445 | 5 | 8.76E-13 | 5.22E-11 | GPX3 | glutathione peroxidase 3 [Source:HGNC Symbol;Acc:HGNC:4555] |
| ENSG00000206503 | 1 | 1.00E-12 | 5.93E-11 | HLA-A | major histocompatibility complex, class I, A [Source:HGNC Symbol;Acc:HGNC:4931] |
| ENSG00000106565 | 4 | 1.00E-12 | 5.93E-11 | TMEM176B | transmembrane protein 176B [Source:HGNC Symbol;Acc:HGNC:29596] |
| ENSG00000173110 | 4 | 1.07E-12 | 6.28E-11 | HSPA6 | heat shock protein family A (Hsp70) member 6 [Source:HGNC Symbol;Acc:HGNC:5239] |
| ENSG00000115523 | 3 | 1.26E-12 | 7.42E-11 | GNLY | granulysin [Source:HGNC Symbol;Acc:HGNC:4414] |
| ENSG00000213614 | 5 | 1.31E-12 | 7.63E-11 | HEXA | hexosaminidase subunit alpha [Source:HGNC Symbol;Acc:HGNC:4878] |
| ENSG00000092068 | 5 | 1.31E-12 | 7.63E-11 | SLC7A8 | solute carrier family 7 member 8 [Source:HGNC Symbol;Acc:HGNC:11066] |
| ENSG00000132475 | 4 | 1.44E-12 | 8.36E-11 | H3F3B | H3 histone family member 3B [Source:HGNC Symbol;Acc:HGNC:4765] |
| ENSG00000223865 | 4 | 1.45E-12 | 8.39E-11 | HLA-DPB1 | major histocompatibility complex, class II, DP beta 1 [Source:HGNC Symbol;Acc:HGNC:4940] |
| ENSG00000172594 | 1 | 1.49E-12 | 8.61E-11 | SMPDL3A | sphingomyelin phosphodiesterase acid like 3A [Source:HGNC Symbol;Acc:HGNC:17389] |
| ENSG00000182004 | 3 | 1.62E-12 | 9.31E-11 | SNRPE | small nuclear ribonucleoprotein polypeptide E [Source:HGNC Symbol;Acc:HGNC:11161] |
| ENSG00000188846 | 3 | 1.62E-12 | 9.31E-11 | RPL14 | ribosomal protein L14 [Source:HGNC Symbol;Acc:HGNC:10305] |
| ENSG00000156411 | 3 | 1.68E-12 | 9.58E-11 | ATP5MPL | ATP synthase membrane subunit 6.8PL [Source:HGNC Symbol;Acc:HGNC:1188] |
| ENSG00000204287 | 4 | 1.68E-12 | 9.58E-11 | HLA-DRA | major histocompatibility complex, class II, DR alpha [Source:HGNC Symbol;Acc:HGNC:4947] |
| SNHG5 | 3 | 1.72E-12 | 9.76E-11 | SNHG5 | - |
| ENSG00000167900 | 3 | 1.87E-12 | 1.06E-10 | TK1 | thymidine kinase 1 [Source:HGNC Symbol;Acc:HGNC:11830] |
| ENSG00000115963 | 2 | 1.89E-12 | 1.06E-10 | RND3 | Rho family GTPase 3 [Source:HGNC Symbol;Acc:HGNC:671] |
| ENSG00000167513 | 3 | 2.14E-12 | 1.20E-10 | CDT1 | chromatin licensing and DNA replication factor 1 [Source:HGNC Symbol;Acc:HGNC:24576] |
| ENSG00000163682 | 3 | 2.25E-12 | 1.26E-10 | RPL9 | ribosomal protein L9 [Source:HGNC Symbol;Acc:HGNC:10369] |
| ENSG00000105205 | 2 | 2.83E-12 | 1.58E-10 | CLC | Charcot-Leyden crystal galectin [Source:HGNC Symbol;Acc:HGNC:2014] |
| ENSG00000135838 | 1 | 2.98E-12 | 1.66E-10 | NPL | N-acetylneuraminate pyruvate lyase [Source:HGNC Symbol;Acc:HGNC:16781] |
| ENSG00000232112 | 3 | 3.50E-12 | 1.94E-10 | TMA7 | translation machinery associated 7 homolog [Source:HGNC Symbol;Acc:HGNC:26932] |
| ENSG00000211897 | 4 | 3.67E-12 | 2.03E-10 | IGHG3 | immunoglobulin heavy constant gamma 3 (G3m marker) [Source:HGNC Symbol;Acc:HGNC:5527] |
| ENSG00000170515 | 3 | 4.34E-12 | 2.39E-10 | PA2G4 | proliferation-associated 2G4 [Source:HGNC Symbol;Acc:HGNC:8550] |
| ENSG00000258227 | 3 | 4.86E-12 | 2.67E-10 | CLEC5A | C-type lectin domain containing 5A [Source:HGNC Symbol;Acc:HGNC:2054] |
| ENSG00000116251 | 3 | 4.97E-12 | 2.72E-10 | RPL22 | ribosomal protein L22 [Source:HGNC Symbol;Acc:HGNC:10315] |
| ENSG00000130332 | 3 | 5.14E-12 | 2.80E-10 | LSM7 | LSM7 homolog, U6 small nuclear RNA and mRNA degradation associated [Source:HGNC Symbol;Acc:HGNC:20470] |
| ENSG00000147614 | 1 | 5.43E-12 | 2.95E-10 | ATP6V0D2 | ATPase H+ transporting V0 subunit d2 [Source:HGNC Symbol;Acc:HGNC:18266] |
| ENSG00000163191 | 2 | 5.45E-12 | 2.96E-10 | S100A11 | S100 calcium binding protein A11 [Source:HGNC Symbol;Acc:HGNC:10488] |
| ENSG00000105697 | 3 | 6.28E-12 | 3.40E-10 | HAMP | hepcidin antimicrobial peptide [Source:HGNC Symbol;Acc:HGNC:15598] |
| ENSG00000143322 | 2 | 6.48E-12 | 3.49E-10 | ABL2 | ABL proto-oncogene 2, non-receptor tyrosine kinase [Source:HGNC Symbol;Acc:HGNC:77] |
| ENSG00000198886 | 5 | 7.33E-12 | 3.94E-10 | MT-ND4 | mitochondrially encoded NADH:ubiquinone oxidoreductase core subunit 4 [Source:HGNC Symbol;Acc:HGNC:7459] |
| ENSG00000130592 | 3 | 8.00E-12 | 4.28E-10 | LSP1 | lymphocyte-specific protein 1 [Source:HGNC Symbol;Acc:HGNC:6707] |
| ENSG00000124491 | 4 | 1.16E-11 | 6.17E-10 | F13A1 | coagulation factor XIII A chain [Source:HGNC Symbol;Acc:HGNC:3531] |
| ENSG00000158270 | 5 | 1.29E-11 | 6.87E-10 | COLEC12 | collectin subfamily member 12 [Source:HGNC Symbol;Acc:HGNC:16016] |
| ENSG00000125835 | 3 | 1.65E-11 | 8.73E-10 | SNRPB | small nuclear ribonucleoprotein polypeptides B and B1 [Source:HGNC Symbol;Acc:HGNC:11153] |
| ENSG00000170296 | 3 | 1.74E-11 | 9.17E-10 | GABARAP | GABA type A receptor-associated protein [Source:HGNC Symbol;Acc:HGNC:4067] |
| ENSG00000187474 | 4 | 1.78E-11 | 9.37E-10 | FPR3 | formyl peptide receptor 3 [Source:HGNC Symbol;Acc:HGNC:3828] |
| ENSG00000163739 | 4 | 1.91E-11 | 1.00E-09 | CXCL1 | C-X-C motif chemokine ligand 1 [Source:HGNC Symbol;Acc:HGNC:4602] |
| ENSG00000125743 | 3 | 2.02E-11 | 1.06E-09 | SNRPD2 | small nuclear ribonucleoprotein D2 polypeptide [Source:HGNC Symbol;Acc:HGNC:11159] |
| ENSG00000166710 | 5 | 2.11E-11 | 1.10E-09 | B2M | beta-2-microglobulin [Source:HGNC Symbol;Acc:HGNC:914] |
| ENSG00000175445 | 2 | 2.40E-11 | 1.25E-09 | LPL | lipoprotein lipase [Source:HGNC Symbol;Acc:HGNC:6677] |
| ENSG00000065978 | 2 | 2.43E-11 | 1.26E-09 | YBX1 | Y-box binding protein 1 [Source:HGNC Symbol;Acc:HGNC:8014] |
| ENSG00000142676 | 3 | 2.78E-11 | 1.44E-09 | RPL11 | ribosomal protein L11 [Source:HGNC Symbol;Acc:HGNC:10301] |
| ENSG00000090104 | 4 | 3.03E-11 | 1.56E-09 | RGS1 | regulator of G protein signaling 1 [Source:HGNC Symbol;Acc:HGNC:9991] |
| GGTA1P | 4 | 3.05E-11 | 1.57E-09 | GGTA1P | - |
| ENSG00000198931 | 3 | 3.12E-11 | 1.60E-09 | APRT | adenine phosphoribosyltransferase [Source:HGNC Symbol;Acc:HGNC:626] |
| ENSG00000135390 | 3 | 3.59E-11 | 1.83E-09 | ATP5MC2 | ATP synthase membrane subunit c locus 2 [Source:HGNC Symbol;Acc:HGNC:842] |
| ENSG00000164109 | 3 | 3.60E-11 | 1.83E-09 | MAD2L1 | mitotic arrest deficient 2 like 1 [Source:HGNC Symbol;Acc:HGNC:6763] |
| ENSG00000105669 | 3 | 3.69E-11 | 1.87E-09 | COPE | coatomer protein complex subunit epsilon [Source:HGNC Symbol;Acc:HGNC:2234] |
| ENSG00000164587 | 3 | 3.98E-11 | 2.01E-09 | RPS14 | ribosomal protein S14 [Source:HGNC Symbol;Acc:HGNC:10387] |
| ENSG00000163221 | 3 | 4.16E-11 | 2.10E-09 | S100A12 | S100 calcium binding protein A12 [Source:HGNC Symbol;Acc:HGNC:10489] |
| ENSG00000156482 | 3 | 4.54E-11 | 2.29E-09 | RPL30 | ribosomal protein L30 [Source:HGNC Symbol;Acc:HGNC:10333] |
| ENSG00000137818 | 3 | 4.80E-11 | 2.41E-09 | RPLP1 | ribosomal protein lateral stalk subunit P1 [Source:HGNC Symbol;Acc:HGNC:10372] |
| ENSG00000100234 | 1 | 4.83E-11 | 2.41E-09 | TIMP3 | TIMP metallopeptidase inhibitor 3 [Source:HGNC Symbol;Acc:HGNC:11822] |
| ENSG00000109320 | 4 | 4.96E-11 | 2.48E-09 | NFKB1 | nuclear factor kappa B subunit 1 [Source:HGNC Symbol;Acc:HGNC:7794] |
| ENSG00000138135 | 5 | 5.59E-11 | 2.78E-09 | CH25H | cholesterol 25-hydroxylase [Source:HGNC Symbol;Acc:HGNC:1907] |
| ENSG00000114942 | 3 | 6.11E-11 | 3.03E-09 | EEF1B2 | eukaryotic translation elongation factor 1 beta 2 [Source:HGNC Symbol;Acc:HGNC:3208] |
| ENSG00000102317 | 3 | 6.49E-11 | 3.21E-09 | RBM3 | RNA binding motif protein 3 [Source:HGNC Symbol;Acc:HGNC:9900] |
| ENSG00000204472 | 4 | 7.40E-11 | 3.65E-09 | AIF1 | allograft inflammatory factor 1 [Source:HGNC Symbol;Acc:HGNC:352] |
| ENSG00000135074 | 3 | 9.23E-11 | 4.53E-09 | ADAM19 | ADAM metallopeptidase domain 19 [Source:HGNC Symbol;Acc:HGNC:197] |
| ENSG00000088827 | 5 | 9.51E-11 | 4.66E-09 | SIGLEC1 | sialic acid binding Ig like lectin 1 [Source:HGNC Symbol;Acc:HGNC:11127] |
| ENSG00000100526 | 3 | 9.72E-11 | 4.75E-09 | CDKN3 | cyclin dependent kinase inhibitor 3 [Source:HGNC Symbol;Acc:HGNC:1791] |
| ENSG00000132432 | 3 | 9.78E-11 | 4.77E-09 | SEC61G | Sec61 translocon gamma subunit [Source:HGNC Symbol;Acc:HGNC:18277] |
| ENSG00000198786 | 2 | 1.06E-10 | 5.13E-09 | MT-ND5 | mitochondrially encoded NADH:ubiquinone oxidoreductase core subunit 5 [Source:HGNC Symbol;Acc:HGNC:7461] |
| ENSG00000198034 | 3 | 1.25E-10 | 6.04E-09 | RPS4X | ribosomal protein S4 X-linked [Source:HGNC Symbol;Acc:HGNC:10424] |
| ENSG00000110079 | 4 | 1.25E-10 | 6.06E-09 | MS4A4A | membrane spanning 4-domains A4A [Source:HGNC Symbol;Acc:HGNC:13371] |
| ENSG00000106588 | 3 | 1.32E-10 | 6.34E-09 | PSMA2 | proteasome subunit alpha 2 [Source:HGNC Symbol;Acc:HGNC:9531] |
| ENSG00000211751 | 3 | 1.40E-10 | 6.73E-09 | TRBC1 | T cell receptor beta constant 1 [Source:HGNC Symbol;Acc:HGNC:12156] |
| ENSG00000257764 | 3 | 1.43E-10 | 6.85E-09 | AC020656.1 | - |
| ENSG00000165029 | 4 | 1.47E-10 | 7.00E-09 | ABCA1 | ATP binding cassette subfamily A member 1 [Source:HGNC Symbol;Acc:HGNC:29] |
| ENSG00000071082 | 3 | 1.51E-10 | 7.17E-09 | RPL31 | ribosomal protein L31 [Source:HGNC Symbol;Acc:HGNC:10334] |
| ENSG00000117724 | 3 | 1.53E-10 | 7.27E-09 | CENPF | centromere protein F [Source:HGNC Symbol;Acc:HGNC:1857] |
| ENSG00000149806 | 3 | 1.57E-10 | 7.46E-09 | FAU | FAU, ubiquitin like and ribosomal protein S30 fusion [Source:HGNC Symbol;Acc:HGNC:3597] |
| ENSG00000173391 | 2 | 1.58E-10 | 7.48E-09 | OLR1 | oxidized low density lipoprotein receptor 1 [Source:HGNC Symbol;Acc:HGNC:8133] |
| ENSG00000108107 | 3 | 1.66E-10 | 7.81E-09 | RPL28 | ribosomal protein L28 [Source:HGNC Symbol;Acc:HGNC:10330] |
| ENSG00000174695 | 3 | 2.06E-10 | 9.68E-09 | TMEM167A | transmembrane protein 167A [Source:HGNC Symbol;Acc:HGNC:28330] |
| ENSG00000214063 | 1 | 2.22E-10 | 1.04E-08 | TSPAN4 | tetraspanin 4 [Source:HGNC Symbol;Acc:HGNC:11859] |
| ENSG00000136938 | 3 | 2.50E-10 | 1.17E-08 | ANP32B | acidic nuclear phosphoprotein 32 family member B [Source:HGNC Symbol;Acc:HGNC:16677] |
| ENSG00000175130 | 3 | 2.53E-10 | 1.18E-08 | MARCKSL1 | MARCKS like 1 [Source:HGNC Symbol;Acc:HGNC:7142] |
| ENSG00000010278 | 2 | 2.95E-10 | 1.37E-08 | CD9 | CD9 molecule [Source:HGNC Symbol;Acc:HGNC:1709] |
| ENSG00000241468 | 3 | 2.96E-10 | 1.37E-08 | ATP5MF | ATP synthase membrane subunit f [Source:HGNC Symbol;Acc:HGNC:848] |
| ENSG00000157168 | 3 | 3.45E-10 | 1.59E-08 | NRG1 | neuregulin 1 [Source:HGNC Symbol;Acc:HGNC:7997] |
| ENSG00000107372 | 5 | 3.70E-10 | 1.70E-08 | ZFAND5 | zinc finger AN1-type containing 5 [Source:HGNC Symbol;Acc:HGNC:13008] |
| ENSG00000106211 | 4 | 3.95E-10 | 1.81E-08 | HSPB1 | heat shock protein family B (small) member 1 [Source:HGNC Symbol;Acc:HGNC:5246] |
| ENSG00000041357 | 3 | 3.94E-10 | 1.81E-08 | PSMA4 | proteasome subunit alpha 4 [Source:HGNC Symbol;Acc:HGNC:9533] |
| ENSG00000148303 | 3 | 4.09E-10 | 1.87E-08 | RPL7A | ribosomal protein L7a [Source:HGNC Symbol;Acc:HGNC:10364] |
| ENSG00000119705 | 3 | 4.60E-10 | 2.09E-08 | SLIRP | SRA stem-loop interacting RNA binding protein [Source:HGNC Symbol;Acc:HGNC:20495] |
| ENSG00000115009 | 2 | 4.83E-10 | 2.19E-08 | CCL20 | C-C motif chemokine ligand 20 [Source:HGNC Symbol;Acc:HGNC:10619] |
| ENSG00000143387 | 5 | 4.86E-10 | 2.20E-08 | CTSK | cathepsin K [Source:HGNC Symbol;Acc:HGNC:2536] |
| ENSG00000167283 | 3 | 5.32E-10 | 2.41E-08 | ATP5MG | ATP synthase membrane subunit g [Source:HGNC Symbol;Acc:HGNC:14247] |
| ENSG00000100387 | 3 | 5.48E-10 | 2.47E-08 | RBX1 | ring-box 1 [Source:HGNC Symbol;Acc:HGNC:9928] |
| ENSG00000164096 | 3 | 5.54E-10 | 2.49E-08 | C4orf3 | chromosome 4 open reading frame 3 [Source:HGNC Symbol;Acc:HGNC:19225] |
| ENSG00000132646 | 3 | 5.58E-10 | 2.50E-08 | PCNA | proliferating cell nuclear antigen [Source:HGNC Symbol;Acc:HGNC:8729] |
| ENSG00000204252 | 4 | 7.36E-10 | 3.29E-08 | HLA-DOA | major histocompatibility complex, class II, DO alpha [Source:HGNC Symbol;Acc:HGNC:4936] |
| ENSG00000181163 | 3 | 8.05E-10 | 3.59E-08 | NPM1 | nucleophosmin 1 [Source:HGNC Symbol;Acc:HGNC:7910] |
| ENSG00000237541 | 4 | 8.51E-10 | 3.79E-08 | HLA-DQA2 | major histocompatibility complex, class II, DQ alpha 2 [Source:HGNC Symbol;Acc:HGNC:4943] |
| ENSG00000170860 | 3 | 8.84E-10 | 3.92E-08 | LSM3 | LSM3 homolog, U6 small nuclear RNA and mRNA degradation associated [Source:HGNC Symbol;Acc:HGNC:17874] |
| ENSG00000090659 | 4 | 1.01E-09 | 4.46E-08 | CD209 | CD209 molecule [Source:HGNC Symbol;Acc:HGNC:1641] |
| ENSG00000126267 | 3 | 1.02E-09 | 4.46E-08 | COX6B1 | cytochrome c oxidase subunit 6B1 [Source:HGNC Symbol;Acc:HGNC:2280] |
| ENSG00000118855 | 4 | 1.01E-09 | 4.46E-08 | MFSD1 | major facilitator superfamily domain containing 1 [Source:HGNC Symbol;Acc:HGNC:25874] |
| ENSG00000253522 | 2 | 1.02E-09 | 4.46E-08 | MIR3142HG | MIR3142 host gene [Source:HGNC Symbol;Acc:HGNC:51944] |
| ENSG00000147123 | 3 | 1.28E-09 | 5.59E-08 | NDUFB11 | NADH:ubiquinone oxidoreductase subunit B11 [Source:HGNC Symbol;Acc:HGNC:20372] |
| ENSG00000106153 | 3 | 1.31E-09 | 5.73E-08 | CHCHD2 | coiled-coil-helix-coiled-coil-helix domain containing 2 [Source:HGNC Symbol;Acc:HGNC:21645] |
| ENSG00000170312 | 3 | 1.34E-09 | 5.82E-08 | CDK1 | cyclin dependent kinase 1 [Source:HGNC Symbol;Acc:HGNC:1722] |
| ENSG00000116288 | 3 | 1.34E-09 | 5.82E-08 | PARK7 | Parkinsonism associated deglycase [Source:HGNC Symbol;Acc:HGNC:16369] |
| ENSG00000124882 | 3 | 1.37E-09 | 5.95E-08 | EREG | epiregulin [Source:HGNC Symbol;Acc:HGNC:3443] |
| ENSG00000213719 | 3 | 1.48E-09 | 6.41E-08 | CLIC1 | chloride intracellular channel 1 [Source:HGNC Symbol;Acc:HGNC:2062] |
| ENSG00000163106 | 4 | 1.53E-09 | 6.60E-08 | HPGDS | hematopoietic prostaglandin D synthase [Source:HGNC Symbol;Acc:HGNC:17890] |
| ENSG00000117399 | 3 | 1.59E-09 | 6.85E-08 | CDC20 | cell division cycle 20 [Source:HGNC Symbol;Acc:HGNC:1723] |
| ENSG00000197958 | 3 | 1.63E-09 | 6.99E-08 | RPL12 | ribosomal protein L12 [Source:HGNC Symbol;Acc:HGNC:10302] |
| ENSG00000164825 | 2 | 1.68E-09 | 7.18E-08 | DEFB1 | defensin beta 1 [Source:HGNC Symbol;Acc:HGNC:2766] |
| ENSG00000134057 | 3 | 1.70E-09 | 7.24E-08 | CCNB1 | cyclin B1 [Source:HGNC Symbol;Acc:HGNC:1579] |
| ENSG00000122406 | 3 | 1.98E-09 | 8.44E-08 | RPL5 | ribosomal protein L5 [Source:HGNC Symbol;Acc:HGNC:10360] |
| ENSG00000124795 | 3 | 2.08E-09 | 8.85E-08 | DEK | DEK proto-oncogene [Source:HGNC Symbol;Acc:HGNC:2768] |
| ENSG00000111786 | 3 | 2.22E-09 | 9.40E-08 | SRSF9 | serine and arginine rich splicing factor 9 [Source:HGNC Symbol;Acc:HGNC:10791] |
| ENSG00000085063 | 5 | 2.32E-09 | 9.78E-08 | CD59 | CD59 molecule (CD59 blood group) [Source:HGNC Symbol;Acc:HGNC:1689] |

**Table S7. The TFs and numbers of their regulatory genes in Branch 1 and 2**

| **Branch 2** | **Number of regulatory genes** | **Branch 1** | **Number of regulatory genes** |
| --- | --- | --- | --- |
| ATF3 | 15 | ATF3 | 5 |
| BCLAF1 | 3 | BCLAF1 | 4 |
| BHLHE40 | 1 | BHLHE40 | 0 |
| CEBPB | 2 | CEBPB | 3 |
| CHD1 | 1 | CHD1 | 1 |
| CREM | 8 | CREM | 3 |
| EGR1 | 5 | EGR1 | 4 |
| ELF1 | 12 | ELF1 | 6 |
| ETS2 | 8 | ETS2 | 3 |
| FOS | 1 | FOS | 2 |
| FOSL2 | 7 | FOSL2 | 1 |
| FOXN3 | 1 | FOXN3 | 0 |
| HDAC2 | 6 | HDAC2 | 3 |
| HIF1A | 3 | HIF1A | 2 |
| IRF1 | 4 | IRF1 | 3 |
| IRF7 | 1 | IRF7 | 0 |
| IRF8 | 4 | IRF8 | 1 |
| JUN | 0 | JUN | 1 |
| JUNB | 4 | JUNB | 2 |
| JUND | 10 | JUND | 3 |
| KLF4 | 2 | KLF4 | 0 |
| MAF | 1 | MAF | 2 |
| MAFF | 1 | MAFF | 2 |
| MAX | 2 | MAX | 0 |
| MXD1 | 2 | MXD1 | 2 |
| NFKB1 | 4 | NFKB1 | 3 |
| NR3C1 | 2 | NR3C1 | 0 |
| PRDM1 | 9 | PRDM1 | 8 |
| RAD21 | 9 | RAD21 | 3 |
| REL | 10 | REL | 4 |
| RUNX1 | 6 | RUNX1 | 2 |
| SPI1 | 14 | SPI1 | 10 |
| STAT1 | 5 | STAT1 | 3 |
| TAF7 | 5 | TAF7 | 4 |
| TFEC | 1 | TFEC | 0 |
| TGIF1 | 2 | TGIF1 | 0 |
| USF2 | 2 | USF2 | 1 |
| XBP1 | 5 | XBP1 | 4 |
| YY1 | 2 | YY1 | 1 |
